# Synthesis of New Hydrazone Derivatives for MAO Enzymes Inhibitory Activity

**DOI:** 10.3390/molecules22081381

**Published:** 2017-08-20

**Authors:** Nafiz Öncü Can, Derya Osmaniye, Serkan Levent, Begüm Nurpelin Sağlık, Beril İnci, Sinem Ilgın, Yusuf Özkay, Zafer Asım Kaplancıklı

**Affiliations:** 1 Department of Analytical Chemistry, Faculty of Pharmacy, Anadolu Universty, 26470 Eskişehir, Turkey; nafizoc@anadolu.edu.tr; 2 Department of Pharmaceutical Chemistry, Faculty of Pharmacy, Anadolu Universty, 26470 Eskişehir, Turkey; dosmaniye@anadolu.edu.tr (D.O.); serkanlevent@anadolu.edu.tr (S.L.); bnsaglik@anadolu.edu.tr (B.N.S.); zakaplan@anadolu.edu.tr (Z.A.K.); 3 Doping and Narcotic Compounds Analysis Laboratory, Faculty of Pharmacy, Anadolu Universty, 26470 Eskişehir, Turkey; 4 Department of Pharmaceutical Toxicology, Faculty of Pharmacy, Anadolu Universty, 26470 Eskişehir, Turkey; inci57@anadolu.edu.tr (B.İ.); silgin@anadolu.edu.tr (S.I.)

**Keywords:** hydrazone, MAO enzymes inhibition, docking studies, genotoxicity, cytotoxicity

## Abstract

In the present work, 14 new 1-substituted-2-phenylhydrazone derivatives were synthesized to evaluate their inhibitory activity against *h*MAO enzymes. The structures of the newly synthesized hydrazones **2a**–**2n** were characterized by IR, ^1^H-NMR, ^13^C-NMR, HR-MS spectroscopic methods. The inhibitory activity of compounds **2a**–**2n** against *h*MAO-A and *h*MAO-B enzymes was elucidated by using an in-vitro Amplex Red^®^ reagent assay based on fluorometric methods. According to the activity studies, **2a** and **2b** were found to be the most active compounds against *h*MAO-A enzyme, with IC_50_ values of 0.342 µM and 0.028 µM, respectively. The most active compounds **2a**–**2b** were evaluated by means of enzyme kinetics and docking studies. Moreover, these compounds were subjected to cytotoxicity and genotoxicity tests to establish their preliminary toxicological profiles and were found to be non-cytotoxic and non-genotoxic. Consequently, the findings of this study display the biological importance of compounds **2a**, **2b** as selective, irreversible and competitive inhibitors of *h*MAO-A. Docking studies revealed that there is a strong interaction between *h*MAO-A and the most active compound **2b**.

## 1. Introduction

Monoamine oxidase (MAO), including flavin adenine dinucleotide as a cofactor (FAD-AOs), is a mitochondrial enzyme that participates in the oxidative deamination of various monoamines such as dopamine, serotonin, adrenaline and noradrenaline. This enzyme consists of two isoforms which are encoded by two different genes and identified as MAO-A and MAO-B [1,2]. MAO-A preferably deaminates serotonin and noradrenaline, while MAO-B gives preference to benzylamine and phenyl ethylamine as a substrate. In addition, dopamine and tyramine are substrates of both isoenzymes regardless of their concentration [3,4].

Selective MAO-A inhibitors are preferred as powerful antidepressant agents, whereas selective MAO-B inhibitors are used as effective agents against Parkinson’s disease [5,6]. Rapid consumption of brain monoamines is one of the causes of depression Positron emission tomography studies indicate that there is a substantial increment of MAO-A in the brain of patients with depression in contrast to healthy people [7,8].

**Figure 1:**
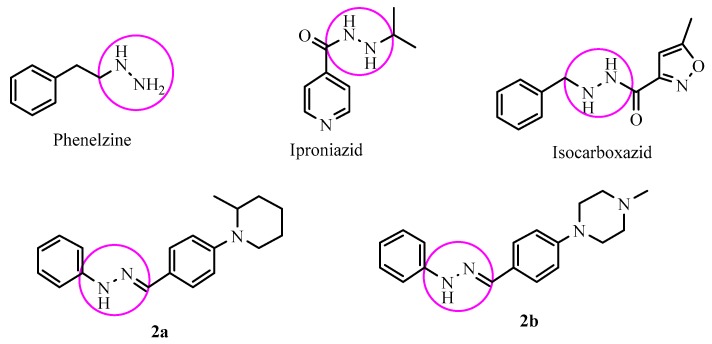
Structures of some MAO inhibitors and the synthesized compounds **2a**, **2b**.

Iproniazid, phenelzine and isocarboxazide (Figure 1) are some of the first improved agents known as the hydrazide/hydrazine class of MAO enzyme inhibitors. These drugs cause an irreversible inhibition owing to the formation of a covalent bond with flavin coenzyme in both isoforms [9,10,11,12]. Due to the irreversible inhibition, there are several reported side effects as hypotension, increased bodyweight, sleeplessness, hypertension, hyperpyrexia and hepatotoxicity [13,14,15]. Thus, for the depression therapy, there is a need to develop a selective and reversible MAO-A inhibitor with a reduced side effect profile.

The potential of hydrazine-type inhibitors can be explained by their structural similarity to MAO substrates, which usually carry an amino or imino group. These inhibitors play a fundamental role in the orientation and complex formation at the active site of the enzyme. Hydrazones are a class of hydrazine analogues, which bear an azomethine -NHN=CH- group. The C=N double bond of hydrazone and terminal nitrogen atom significantly influence the physical and chemical properties. The C-atom in hydrazone has both electrophilic and nucleophilic properties. Both of nitrogen atoms of the hydrazone group have nucleophilic character, whereas the amino type nitrogen is more reactive [16,17,18]. Due to the described chemical properties of hydrazones, recent studies set light to several substituted hydrazones as MAO inhibitors [19,20,21,22]. Prompted by the MAO inhibitory potency of hydrazones, in this work a series of 1-substituted-2-phenylhydrazone were synthesized and evaluated for their MAO inhibitor activities.

## 2. Results and Discussion

### 2.1. Chemistry

**Scheme 1:**
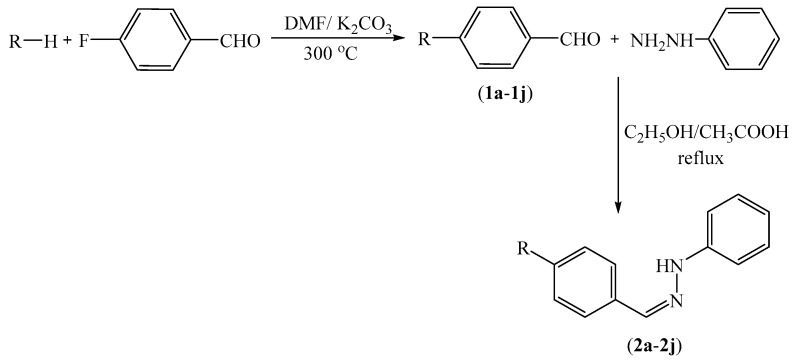
Synthesis of target compounds **2a**–**2n**.

The compounds **2a**–**2n** were synthesized as summarized in Scheme 1. 4-Substituted benzaldehyde derivatives **1a**–**1n** were synthesized by the reactions of 4-fluorobenzaldehyde and appropriate proton donating compounds under reflux. Phenylhydrazine and the 4-substituted benzaldehydes **1a**–**1n** were then reacted in order to obtain the target compounds **2a**–**2n**. Structure elucidations of the final compounds were performed by IR, ^1^H-NMR, ^13^C-NMR, and HRMS spectroscopic methods (see Supplementary Materials). In the IR spectra, stretching absorptions at 3269–3350 cm^−1^ indicated the N-H bonds of the hydrazone groups. The stretching absorption at about 1228–1273 cm^−1^ were attributed to C-N single bonds. The out of plane bending bands of the 1,4-disubstituted benzene were observed at 744–831 cm^−1^. In the ^1^H-NMR spectra, aromatic protons of benzene, imidazole and triazole rings were recorded between 6.70 ppm and 9.33 ppm. The *N*-benzylidene substructure had characteristic two triplet and one doublet peaks. However, in some cases, they overlapped with other aromatic peaks. Besides, the 1,4-disubstituted phenyl rings have two typical doublet peaks. The protons on the hydrazide carbon were recorded as singlets between 7.80 ppm and 8.25 ppm. The signal of the N-H proton on the hydrazide moiety appeared above 10.02 ppm. In the ^13^C-NMR spectra, all aromatic carbons gave peaks from 112 ppm to 163 ppm. In fluorinated derivatives, (compounds **2g** and **2h**) carbon-fluorine coupling was observed. In the HRMS spectra, all masses matched well with the expected M + H values.

**Compounds****R****2a**2-Methylpiperidinyl**2b**4-Methylpiperazinyl**2c**4-Phenylpiperazinyl**2d**1-(4-Methoxyphenyl)piperazinyl**2e**4-Methoxyphenoxy**2f**4-Methoxyphenylthio**2g**4-Fluorophenoxy**2h**4-Fluorophenylthio**2i**imidazolyl**2j**triazolyl**2k**4-Chlorophenylthio**2l**4-Benzylpiperidinyl**2m**4-(2-Dimethylaminoethyl)piperazinyl
**2n**4-(3-Dimethylaminopropyl)piperazinyl

### 2.2. Enzymatic Studies

#### 2.2.1. MAO-A and MAO-B Inhibition Assay

The synthesized compounds **2a**–**2n** were investigated for their *h*MAO-A and *h*MAO-B inhibitory activity by an in vitro fluorometric method, which allows one to sensitively detect monoamine oxidase (MAO) activity. The assay is based on the detection of H_2_O_2_ in a horseradish peroxidase- coupled reaction using 10-acetyl-3,7-dihydroxyphenoxazine (Amplex Red) reagent. The assay was performed in two steps. First, compounds **2a**–**2n** were tested at 10^−3^ and 10^−4^ M concentrations. The second step was performed by using 10^−5^–10^−9^ M concentrations of selected compounds that indicated more than 50% inhibitory activity at the initial concentrations. Table 1 presents the *h*MAO-A and *h*MAO-B inhibitory activity of compounds **2a**–**2n**.

None of the synthesized compounds showed high inhibitory potency against *h*MAO-B. Thus, they did not pass the first step test. On the other hand, in the initial assay, compounds **2a** and **2b** displayed more than 50% inhibition against *h*MAO-A and thus were evaluated in the second step assay, in which IC_50_ values of 0.342 and 0.028 μM were recorded. Moclobemide, a standard drug against *h*MAO-A, had an IC_50_ of 6.061 μM, whereas an IC_50_ of 0.040 μM was found for the reference drug selegiline against *h*MAO-B (Table 2). These findings revealed that compounds **2a** and **2b** had a significant potency to inhibit *h*MAO-A compared to the reference agent moclobemide. The most active compound **2b** (IC_50_ = 0.028 μM) was found to be 216-fold more active than moclobemide (IC_50_ = 6.061 μM) against *h*MAO-A. Besides, it was observed that the compounds **2a** and **2b** have selective inhibition potency towards *h*MAO-A.

#### 2.2.2. Enzyme Kinetics

**Figure 2:**
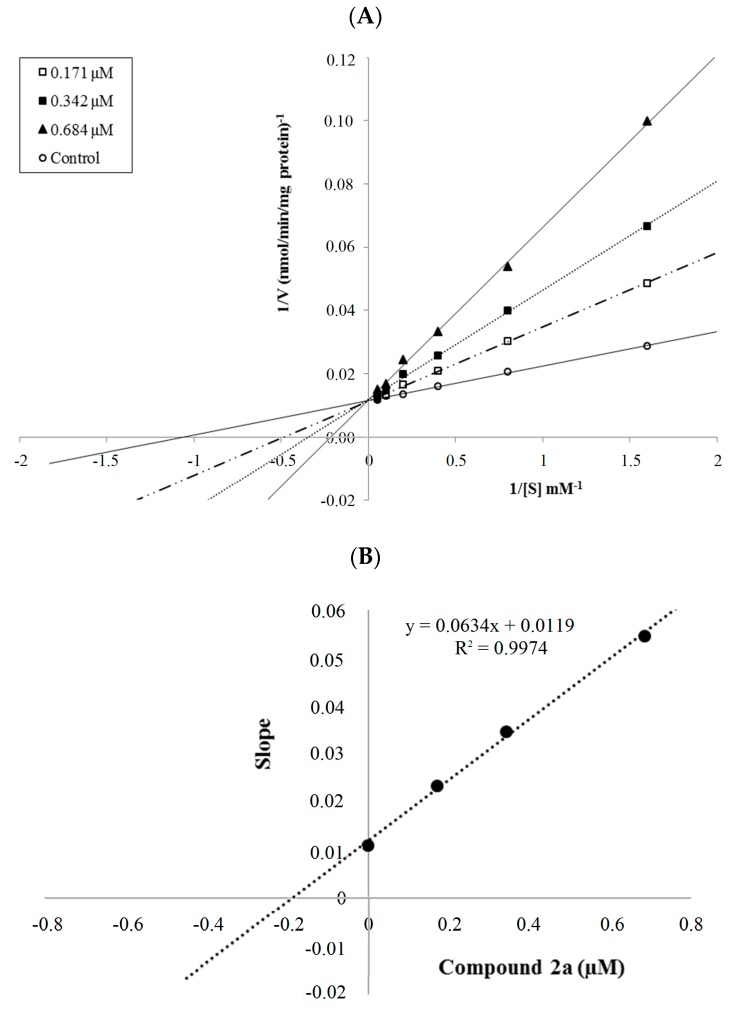
(**A**) Lineweaver-Burk plots for the inhibition of *h*MAO-A by compound **2a**. [S], substrate concentration (μM); V, reaction velocity (nmol/min/mg protein). Inhibitor concentrations (IC_50_/2, IC_50_, and 2 × IC_50_) are shown at the left along with negative control. K_m_ values from IC_50_/2 to Control; 2.071, 2.899, 4.550 and 0.940 (μM). V_max_ value of the competitive inhibition; 85.517 ± 2.332 (nmol/min/mg protein). (**B**) Secondary plot for calculation of steady-state inhibition constant (*K_i_*) of compound **2a**. Equation corresponding to the line at this graph is y = 0.0634x + 0.0119, R^2^ = 0.9974. *K_i_* was calculated as 0.188 μM.

**Figure 3:**
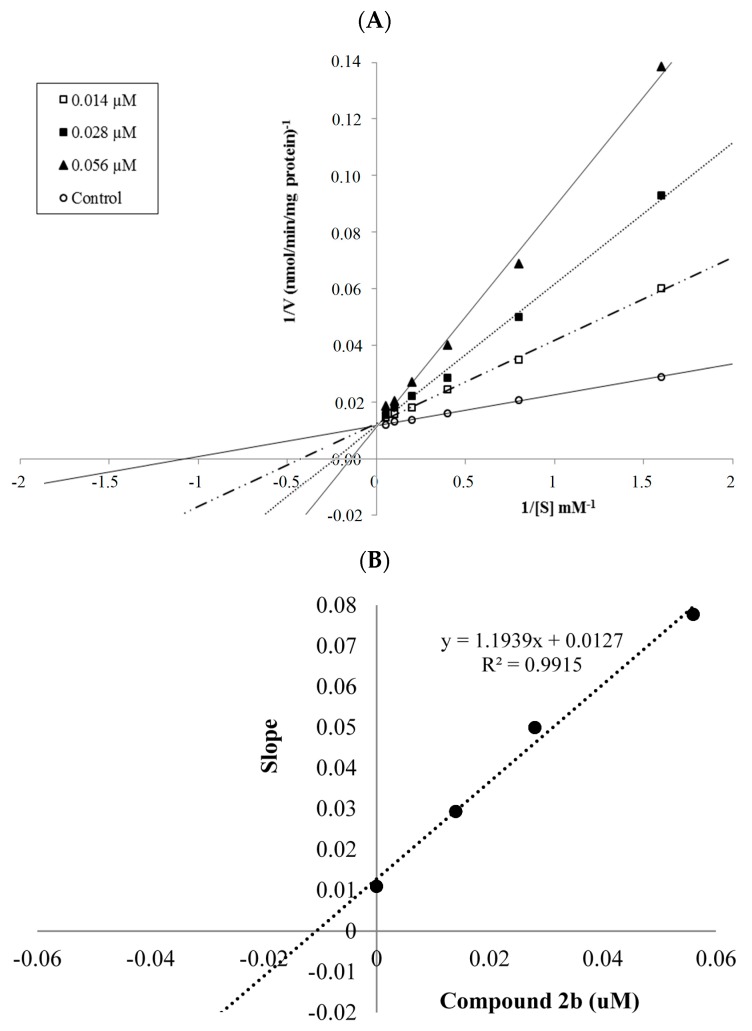
(**A**) Lineweaver-Burk plots for the inhibition of *h*MAO-A by compound **2b**. [S], substrate concentration (μM); V, reaction velocity (nmol/min/mg protein). Inhibitor concentrations (IC_50_/2, IC_50_, and 2 × IC_50_) are shown at the left along with negative control. K_m_ values from IC_50_/2 to control; 2.831, 3.398, 5,014 and 0.940 (μM). V_max_ value of the competitive inhibition; 79.360 ± 5.704 (nmol/min/mg protein). (**B**) Secondary plot for calculation of steady-state inhibition constant (*K_i_*) of compound **2b**. Equation corresponding to the line at this graph is y = 1.1939x + 0.0127, R^2^ = 0.9915. *K_i_* was calculated as 0.011 μM.

The mechanism of *h*MAO-A inhibition was investigated by enzyme kinetics, following a similar procedure to the MAO inhibition assay. The linear Lineweaver-Burk graphics were used to estimate the type of inhibition. Enzyme kinetics were analyzed by recording substrate velocity curves in the absence and presence of the most potent compounds **2a** and **2b**, which were prepared at concentrations of IC_50_/2, IC_50_ and 2 × IC_50_. In each case, the initial velocity measurements were gained at different substrate (tyramine) concentrations ranging from 20 μM to 0.625 μM. The *K_i_* (intercept on the x-axis) values of compounds **2a** and **2b** were determined from the secondary plot of the K_m_/V_max_ (slope) versus varying concentrations. The graphical analysis of steady-state inhibition data for compounds **2a** and **2b** is shown in Figure 2 and Figure 3.

Based on the type of interaction with the enzyme, inhibitor binding can be classified as either reversible or irreversible. The type of inhibition can be determined by the Lineweaver-Burk plot as mixed-type, uncompetitive, competitive, or noncompetitive, which are the indicators of a reversible inhibitor [23]. It is known that in the uncompetitive type inhibition a graphic, including the parallel lines without any cross, is observed. If the lines cross neither the x- nor the y-axis at the same point the inhibition type is called mixed-type. Noncompetitive inhibition is seen if the plots intersect on the x-axis. There are different slopes and intercepts on the y-axis. On the other hand, competitive inhibition gives the opposite situation. This type of inhibition has plots with the same intercept on the y-axis but there are diverse slopes and intercepts on the x-axis, which are observed in Figure 2 and Figure 3. Therefore, this pattern indicates that the compounds **2a** and **2b** are reversible and competitive inhibitors, namely they have similar inhibition features as the substrate. The *K_i_* values for compounds **2a** and **2b** were calculated as 0.188 and 0.016 μM, respectively, for the inhibition of *h*MAO-A.

Reversible inhibitors bind to enzymes by non-covalent interactions such as hydrophobic interactions, ionic bonds, and hydrogen bonds without forming any chemical bonds or reactions with the enzyme. These interactions are formed rapidly and can be easily removed; hence the enzyme and inhibitor complex is quickly dissociated contrary to irreversible inhibition. Due to the reversible binding ability to biomolecules such inhibitors carry a lower risk of side effects compared to irreversible inhibitors. As a result, the reversible-competitive inhibition potency of compounds **2a** and **2b** has enhanced their biological importance in contrast to irreversible hydrazine type MAO inhibitors.

### 2.3. Toxicological Studies

#### 2.3.1. Cytotoxicity Test

The cytotoxicity of compounds **2a** and **2b** was evaluated against a healthy NIH/3T3 mouse embryonic fibroblast cell line (ATCC CRL1658), which is suggested for preliminary cytotoxicity screening by ISO (10993-5, 2009) [24]. The IC_50_ values of the compounds are presented in Table 3. Compounds **2a** and **2b** displayed IC_50_ values of 930 and 20 µM against NIH/3T3 cells, which are significantly higher than their IC_50_ values (0.342 and 0.028) against *h*MAO-A. This result reveals that compounds **2a** and **2b** are not cytotoxic at their effective concentration against *h*MAO-A, which improves the biological importance of both compounds.

#### 2.3.2. Genotoxicity Test

An Ames MPF assay was performed to investigate the genotoxicity of the compounds **2a** and **2b**. In this assay, more than 25 positive wells were observed with our positive controls. Negative control wells also showed less than eight positive wells in the presence and absence of S9 with TA98 and TA100, which complied with the requirements for the validation of the test as described in the previous studies [25]. Our results are presented in Table 4.

**Figure 4:**
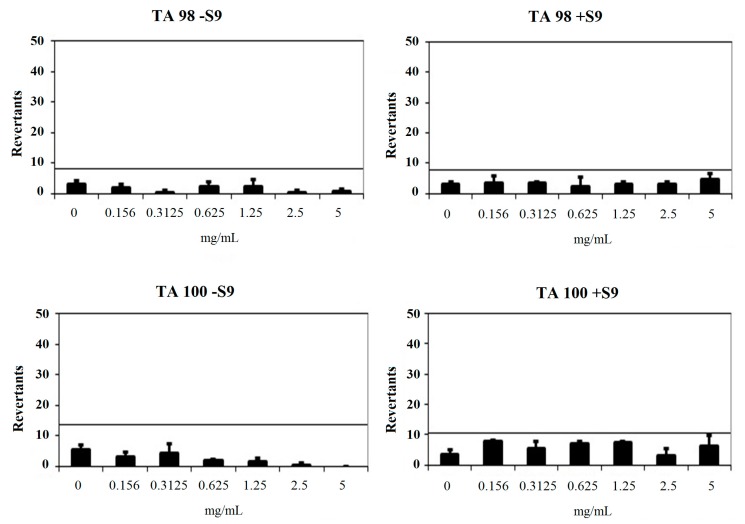
Dose-response curve of compound **2a** against TA98 and TA100 in the presence and absence of S9 according to AMES MPF test.

Compound **2a** had a baseline of 4.00 with TA98 in the absence of S9 and 4.00 in the presence of S9. Fold-inductions over baseline did not reach the mentioned values above the baseline. Also, mentioned-fold increases over the baseline according to the criteria were not determined with this compound against TA98 with S9. Therefore, **2a** was classified as non-mutagenic against TA98 in the presence and absence of metabolic activation (S9). Compound **2a** showed a baseline of 6.86 with TA100 in the absence of S9 and 7.72 in the presence of S9. Fold-inductions over baseline were less than 1.5 in each concentration of the compounds. Also, fold-inductions over baseline did not reach the mentioned values above the baseline. Therefore, **2a** was not genotoxic against TA100 with/without metabolic activation (Figure 4).

**Figure 5:**
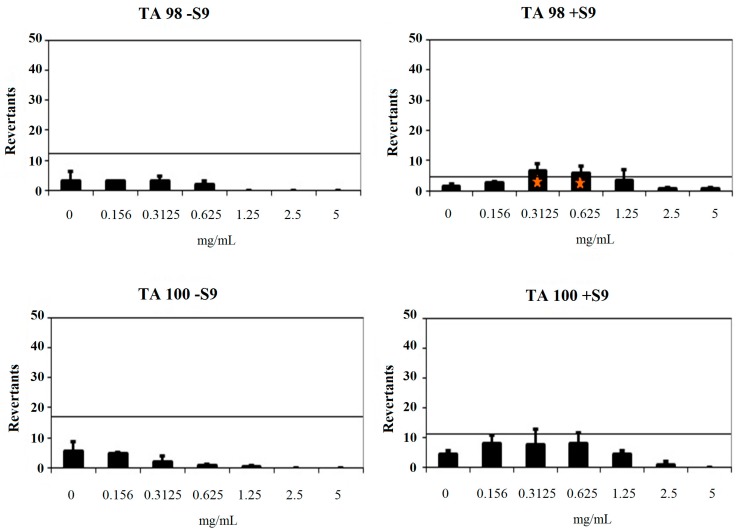
Dose-response curve of compound **2b** against TA98 and TA100 in the presence and absence of S9 according to AMES MPF test. Orange triangle shows t test *p* value (unpaired 1-sided) < 0.05 with > 2-fold induction over baseline.

Compound **2b** showed a baseline of 9.18 and 2.73 against TA98 without/with S9, respectively. Fold-inductions over baseline did not reach values more than 1.5 against TA98 without S9. However, fold-inductions over baseline were more than 1.5 in 0.3125 and 0.625 mg/mL concentrations of the compound against TA98 with S9. Besides, no significant increase at the highest concentration level was observed. Therefore, compound **2b** was not classified as a mutagen against T98 without metabolic activation and TA98 with metabolic activation. Compound **2b** was found to have a baseline of 11.61 and 5.49 with/without S9 against TA100. Mentioned fold-increases over the baseline according to the criteria were not determined with compound **2b** against TA100 without S9. Also, fold-inductions over baseline did not reach values more 1.5 in each concentration of the compounds against TA100 with S9. Thus, compound **2b** was found to be non-mutagenic against TA100 in the absence of metabolic activation and in the presence of metabolic activation (Figure 5). As a result, the Ames MPF assay findings also increase the importance of compounds **2a** and **2b** as *h*MAO-A inhibitor candidates.

### 2.4. Prediction of ADME Parameters and BBB Permeability

Low toxicological effects and an essential pharmacological activity are not enough for a compound to become a drug candidate. It is beneficial to evaluate pharmacokinetic profiles during the early development phases of new drug molecules. In recent years, combinatorial chemistry has considerably increased the number of compounds, for which early data on absorption, distribution, metabolism and excretion (ADME) are needed [26]. Therefore, predictions of ADME properties of the obtained compounds **2a**–**2n** were implemented by online Molinspiration property program [27]. This program applies the Lipinski’s rule of five, which assesses the ADME properties of drug like compounds, and is significant for the optimization of a biologically active compound. In keeping with this rule, an orally active drug has not more than one violation. The theoretical calculations of ADME parameters (topological polar surface area (TPSA), molecular volume (MV), number of hydrogen acceptors (HBA), number of hydrogen donors (HBD), octanol/water partition coefficient (log P), and molecular weight (MW)) are accessible in Table 5 along with the violations (Vio) of Lipinski’s rule. In regard to these data, the obtained compounds **2a**–**2n** fitted Lipinski’s rules by possessing no more than one violation. Accordingly, it can be suggested that the obtained compounds may be have a good pharmacokinetic profile, increasing their pharmacological significance. Drugs that specifically target the CNS must first permeate the blood brain barrier (BBB). Though the BBB is protective in nature, the incapability of drug molecules to permeate the BBB is an important impairment for CNS drug candidates and should be addressed early in the drug discovery progress. Hence, the task of predicting the BBB permeability of new compounds is of a great significance [28]. From this point of assessment, BBB permeability of the synthesized compounds **2a**–**2n** was calculated by a CBLigand-BBB prediction server [29]. This predictor practises two different algorithms as AdaBoost and Support Vector Machine (SVM), combining with four different fingerprints, employed to predict if a compound can pass (+) or cannot pass (−) the BBB. In each case, predictor scores higher than 0, if the compound can pass the BBB. According to Table 5, all calculations for the obtained compounds caused as BBB (+), which is required for MAO inhibitors to display the biological activity.

The hydrolytic stability of test compounds is another important parameter, which affects the biological activity results. It is known that hydrazones are stable in plasma and the stablity rate increases with the aromatic substituents on the imine nitrogen, presumably because of an enhanced electrophilicity of the hydrazone [30]. Thus, it can be revealed that compounds **2a**–**2n** possess the required hydrolytic stability due to presence of phenyl substituents on the imine nitrogen.

### 2.5. Molecular Docking Studies

**Figure 6:**
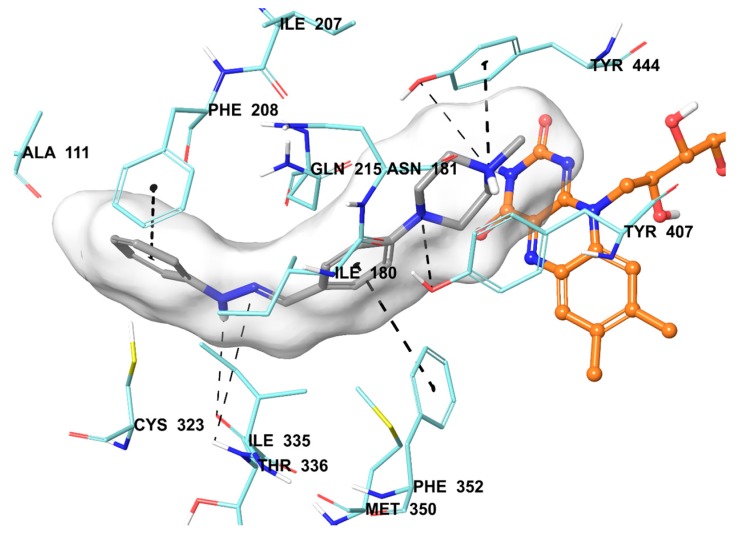
The mode of interaction of compound **2b** in the active region of *h*MAO-A. The inhibitor (grey colored) and the important residues in the active site of the enzyme are presented by a tube model. The FAD molecule is colored orange with a ball and stick model.

**Figure 7:**
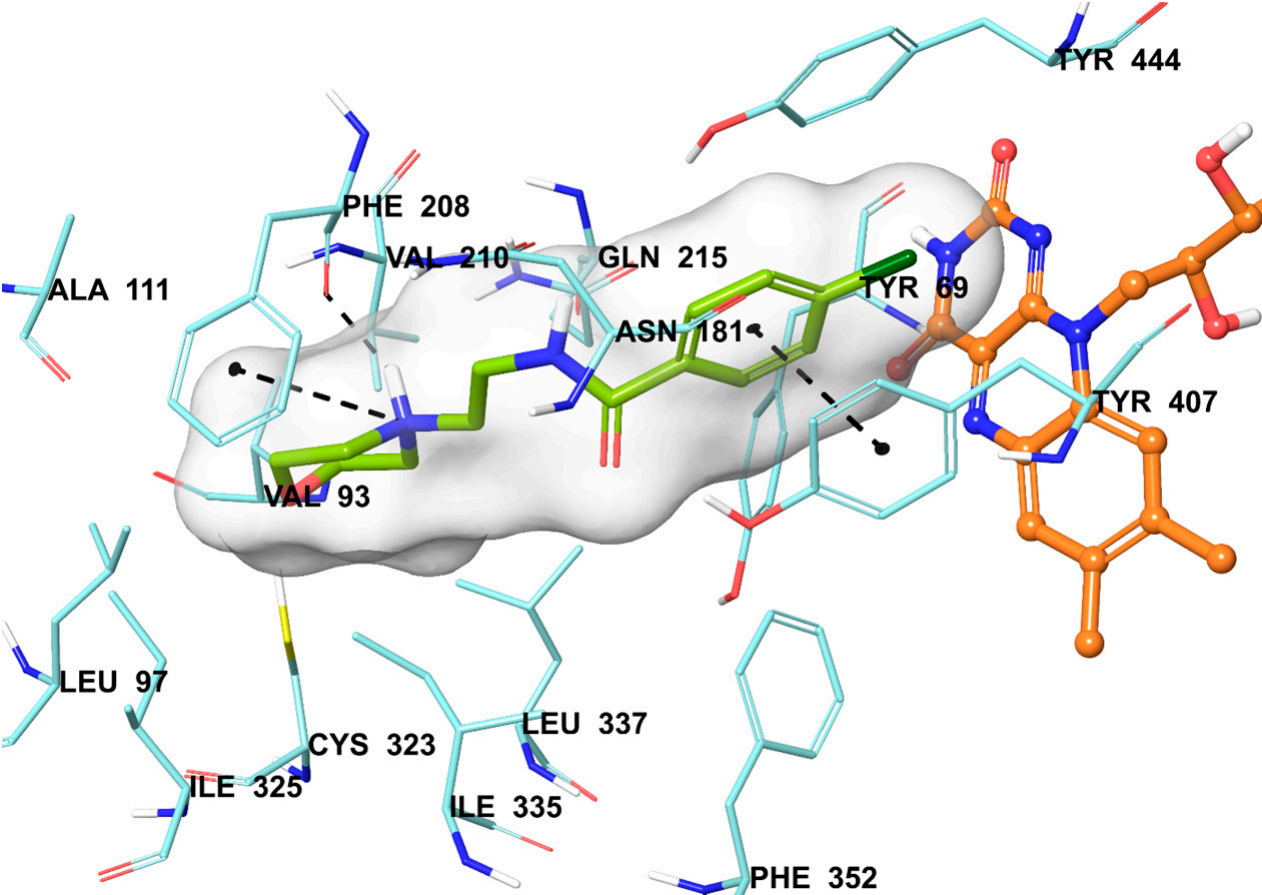
The mode of interaction of moclobemide in the active region of *h*MAO-A. The inhibitor (green colored), and the important residues in the active site of the enzyme are presented by tube models. The FAD molecule is colored orange with a ball and stick model.

The compound **2b** were found to be the most active and selective *h*MAO-A inhibitor as mentioned in the MAO inhibition assay. Docking studies were performed in order to gain more insight into the binding modes of compound **2b**, and to evaluate the effects of structural modifications on the inhibitory activity against *h*MAO-A. X-ray crystal structure of *h*MAO-A (PDB ID: 2Z5X) [31] was obtained from Protein Data Bank server (www.pdb.org). The docking poses of compound **2b** and reference agent moclobemide on *h*MAO-A are presented in Figure 6 and Figure 7.

Compound **2b** snugly binds to the amino residues lining the cavity, and is located very near the FAD cofactor. According to the docking pose of compound **2b**, the hydrazone moiety is essential for polar interactions. This group has two nitrogen atoms capable of forming two hydrogen bonds with Thr336. The amino nitrogen creates a hydrogen bond with the carbonyl of Thr336, while the imine nitrogen establishes same bond with the amino of Thr336. These interactions support the approach, which reveals that amino acid side chains, coating the cavity, are very favorable to interact with the amine moieties [32,33,34,35]. The benzene ring attached to hydrazone moiety displays a π-π interaction with Phe352, whereas the benzylidene substructure establishes the same interaction with Phe208. Furthermore, the nitrogen atoms of piperazine are very important in terms of binding to active site. The first nitrogen atom of piperazine forms a hydrogen bond with the hydroxyl of Tyr407. The nitrogen atom of the fourth position shows a cation-π interaction between the phenyl of Tyr444. The hydroxyl of Tyr444 also creates a hydrogen bond with this nitrogen atom.

Once the structures of the synthesized compounds compared to each other, it is seen that the moieties at the fourth positions of benzylidene substructure are the main cause of structural difference. Particularly, it may be suggested that 4-methylpiperazine moiety in the compound **2b** is very important in terms of high *h*MAO-A inhibition. Although, compounds **2c** and **2d** also possess 4-Phenylpiperazine and 4-(4-Methoxyphenyl)piperazine moieties at the fourth position of benzylidene substructure, these compounds could not display inhibitory potency like **2b**. This may be caused by the elongated structures of the compounds **2c**, **2d**, **2m**, and **2n** which could not be accomodated in the enzyme active site.

In addition to compound **2b**, the reference agent moclobemide used in enzyme inhibition assay was also subjected to docking study (Figure 7). When the docking pose of moclobemide was analyzed, it is seen that there are three interactions between this molecule and enzyme active region residues. The phenyl ring creates a π-π interaction with Tyr407. The other interactions are related to morpholine moiety of moclobemide. The nitrogen atom of morpholine have two interactions as cation-π and hydrogen bond with phenyl and carbonyl of Phe208, respectively. The more interactions observed in compound **2b** than moclobemide may explain the higher enzyme inhibitory activity of this compound than reference agent.

## 3. Materials and Methods

### 3.1. General Information

All chemicals were obtained either from Sigma-Aldrich (Sigma-Aldrich Corp., St. Louis, MO, USA) or Merck (Merck KGaA, Darmstadt, Germany), and used without further purification. Melting points of the compounds were measured by using an automatic melting point determination instrument (MP90, Mettler-Toledo, Columbus, OH, USA) and are uncorrected. ^1^H- and ^13^C-NMR spectra were recorded in DMSO-*d_6_* on a Bruker digital FT-NMR spectrometer (Bruker Bioscience, Billerica, MA, USA) at 300 MHz and 75 MHz, respectively. The IR spectra of the compounds were recorded using an IRAffinity-1S Fourier transform IR (FTIR) spectrometer (Shimadzu, Tokyo, Japan). HRMS studies were performed on an LCMS-IT-TOF system (Shimadzu). Chemical purities of the compounds were checked by classical TLC applications performed on silica gel 60 F254 (Merck KGaA).

### 3.2. Chemistry

#### 3.2.1. Synthesis of 4-substituted Benzaldehydes **1a**–**1n**

A mixture of 4-fluorobenzaldehyde (8.85 mL, 0.1 mol), the corresponding phenol, thiophenol or amine (0.1 mol), and a catalytic quantity of potassium carbonate (K_2_CO_3_) was refluxed in DMF (20 mL) for 36 h. After completion of the reaction, the mixture was poured into ice-water (50 mL), and the precipitated product was filtered, washed with deionised water, dried, and recrystallized from EtOH.

#### 3.2.2. General Procedure for the Synthesis of Target Compounds **2a**–**2n**

Phenylhydrazine, the appropriate 4-substituted benzaldehyde derivative **1a**–**1n** and catalytic quantity of acetic acid were refluxed in EtOH for 2 h. The mixture was cooled, precipitated product was filtered, dried, and recrystallized from EtOH.

*1-(4-(2-Methypiperidin-1-yl)benzylidene)-2-phenylhydrazine* (**2a**). Yield: 85%, M.P. = 180.1–185.2 °C, FTIR (ATR, cm^−1^): 3269 (N-H), 2933 (C-H), 1251 (C-N), 823, 748. ^1^H-NMR: δ = 0.97 (3H, d, *J =* 6.63 Hz, -CH_3_), 1.54–1.59 (4H, m, piperidine), 1.71–1.74 (4H, m, piperidine), 2.83–2.89 (1H, m, -CH-), 6.68 (1H, t, *J =* 7.26 Hz, monosubstituted benzene H4), 6.89 (2H, d, *J =* 8.85 Hz, disubstituted benzene CH), 7.01 (2H, d, *J =* 7.53 Hz, monosubstituted benzene H2,2’), 7.18 (2H, t, *J =* 7.89 Hz, monosubstituted benzene H3,3’), 7.46 (2H, d, *J =* 8.85 Hz, disubstituted benzene CH), 7.76 (1H, s, -CH=N-), 10.02 (1H, s, NH). ^13^C-NMR: δ = 13.32, 18.92, 25.94, 31.06, 42.56, 49.69, 112.12, 115.80, 118.43, 125.72, 127.22, 129.47, 137.83, 146.23, 150.99. HRMS (*m*/*z*): [M + H]^+^ calcd for C_19_H_23_N_3_: 294.1965; found: 294.1966.

*1-(4-(4-Methylpiperazine-1-yl)benzylidene)-2-phenylhydrazine* (**2b**). Yield: 83%, M.P. = 166.8–180.9 °C, FTIR (ATR, cm^−1^): 3313 (N-H), 2933 (C-H), 1253 (C-N), 815, 759. ^1^H-NMR: δ = 2.77 (3H, s, -CH_3_), 3.27 (4H, br.s, piperazine), 3.49 (4H, br.s, piperazine), 6.70 (1H, t, *J =* 7.23 Hz, monosubstituted benzene H_4_), 6.99–7.05 (4H, m, monosubstituted benzene H_2,2’_, disubstituted benzene CH), 7.19 (2H, t, *J = 7.29* Hz, monosubstituted benzene H_3,3’_), 7.53 (2H, d, *J = 8.76* Hz, disubstituted benzene CH), 7.83 (1H, s, -CH=N-), 10.25 (1H, s, NH). ^13^C-NMR: δ = 42.46, 45.60, 52.43, 112.22, 116.19, 118.65, 127.15, 127.92, 129.49, 137.19, 146.10, 149.78. HRMS (*m*/*z*): [M + H]^+^ calcd for C_18_H_22_N_4_: 295.1917; found: 295.1924.

*1-(4-(4-Phenylpiperazine-1-yl)benzylidene)-2-phenylhydrazine* (**2c**). Yield: 86%, M.P. = 149.4–160.9 °C, FTIR (ATR, cm^−1^): 3313 (N-H), 2933 (C-H), 1253 (C-N), 815, 7.59. ^1^H-NMR: δ = 3.26-3.27 (4H, m, piperazine), 3.31–3.33 (4H, m, piperazine), 6.71 (1H, t, *J =* 7.20 Hz, monosubstituted benzene H_4_), 6.81 (1H, t, *J =* 7.23 Hz, monosubstituted benzene H_4_), 6.97–7.06 (6H, m, monosubstituted benzene CH, disubstituted benzene CH), 7.18–7.26 (4H, m, monosubstituted benzene CH), 7.53 (2H, d, *J =* 8.79 Hz, disubstituted benzene CH), 7.80 (1H, s, -CH=N-), 10.08 (1H, s, NH). ^13^C-NMR: δ = 48.32, 48.70, 112.20, 115.76, 116.14, 118.60, 119.63, 127.04, 127.17, 129.45, 129.52, 137.55, 146.15, 151.12, 151.35. HRMS (*m*/*z*): [M + H]^+^ calcd for C_23_H_24_N_4_: 357.2074; found: 357.2057.

*1-(4-(4-(4-Methoxyphenyl)piperazine-1-yl)benzylidene)-2-phenylhydrazine* (**2d**). Yield: 81%, M.P. = 141.2–150.4 °C, FTIR (ATR, cm^−1^): 3302 (N-H), 2951 (C-H), 1273 (C-N), 819, 750. ^1^H-NMR: δ = 3.14–3.16 (4H, m, piperazine), 3.31–3.33 (4H, m, piperazine), 3.69 (3H, s, -OCH_3_), 6.70 (1H, t, *J =* 7.23 Hz, monosubstituted benzene H_4_), 6.84 (2H, d, *J =* 9.10 Hz, methoxyphenyl CH), 6.96 (2H, d, *J =* 9.10 Hz, methoxyphenyl CH), 6.99–7.03 (4H, m, monosubstituted benzene H_2,2’_, disubstituted benzene CH), 7.19 (2H, t, *J =* 8.37 Hz, monosubstituted benzene H_3,3’_), 7.52 (2H, d, *J =* 8.76 Hz, disubstituted benzene CH), 7.79 (1H, s, -CH=N-), 10.06 (1H, s, NH). ^13^C-NMR: δ = 48.44, 50.18, 55.66, 112.18, 114.75, 115.73, 118.20, 118.59, 126.98, 127.15, 129.51, 137.56, 145.72, 146.14, 151.17, 151.63. HRMS (*m*/*z*): [M + H]^+^ calcd for C_24_H_26_N_4_O: 387.2179; found: 387.2166.

*1-(4-(4-Methoxyphenoxy)benzylidene)-2-phenylhydrazine* (**2e**). Yield: 83%, M.P. = 170.9–174.9 °C, FTIR (ATR, cm^−1^): 3302 (N-H), 2924 (C-H), 1255 (C-N), 831, 744. ^1^H-NMR: δ = 3.76 (3H, s, -OCH_3_), 6.72 (1H, t, *J = 7.23* Hz, monosubstituted benzene H_4_), 6.93 (2H, d, *J =* 8.64 Hz, methoxyphenyl CH), 6.96–7.05 (6H, m, monosubstituted benzene H_2,2’_, disubstituted benzene CH, methoxyphenyl CH), 7.20 (2H, t, *J =* 8.10 Hz, monosubstituted benzene H_3,3’_), 7.61 (2H, d, *J =* 8.67 Hz, Disubstituted benzene CH), 7.83 (1H, s, -CH=N-), 10.23 (1H, s, NH). ^13^C-NMR: δ = 55.89, 112.33, 115.58, 117.87, 118.98, 121.29, 127.67, 129.54, 130.89, 136.49, 145.87, 149.60, 156.22, 158.40. HRMS (*m*/*z*): [M + H]^+^ calcd for C_20_H_18_N_2_O_2_: 319.1441; found: 319.1448

*1-(4-((4-Methoxyphenyl)thio)benzylidene)-2-phenylhydrazine* (**2f**). Yield: 85%, M.P. = 178.1–184.3 °C, FTIR (ATR, cm^−1^): 3300 (N-H), 2956 (C-H), 1244 (C-N), 815, 742. ^1^H-NMR: δ = 3.79 (3H, s, -OCH_3_), 6.74 (1H, t, *J = 7.26* Hz, monosubstituted benzene H_4_), 6.99–7.06 (4H, m, monosubstituted benzene H_2,2’_, disubstituted benzene CH), 7.13 (2H, d, *J* = 8.43 Hz, methoxyphenyl CH), 7.20 (2H, t, *J* = 7.32 Hz, monosubstituted benzene H_3,3’_), 7.43 (2H, d, *J* = 8.85 Hz, disubstituted benzene CH), 7.56 (2H, d, *J =* 8.46 Hz, methoxyphenyl CH), 7.80 (1H, s, -CH=N-), 10.33 (1H, s, NH). ^13^C-NMR: δ = 55.80, 112.44, 115.87, 119.24, 123.42, 126.79, 128.50, 129.56, 134.28, 135.64, 136.14, 137.73, 145.64, 160.19. HRMS (*m*/*z*): [M + H]^+^ calcd for C_20_H_18_N_2_OS: 335.1213; found: 335.1207.

*1-(4-(4-Fluorophenoxy)benzylidene)-2-phenylhydrazine* (**2g**). Yield: 81%, M.P. = 121.3–124.9 °C, FTIR (ATR, cm^−1^): 3315 (N-H), 2951 (C-H), 1251 (C-N), 821, 752. ^1^H-NMR: δ = 6.73 (1H, t, *J =* 7.23 Hz, monosubstituted benzene H_4_), 6.99 (2H, d, *J =* 8.73 Hz, monosubstituted benzene H_2,2’_), 7.03–7.12 (4H, m, disubstituted benzene CH, fluorophenyl CH), 7.18–7.28 (4H, m, monosubstituted benzene H_3,3’_, fluorophenyl CH), 7.65 (2H, d, *J =* 8.76 Hz, disubstituted benzene CH), 7.85 (1H, s, -CH=N-), 10.27 (1H, s, NH). ^13^C-NMR: δ = 112.36, 116.94, 117.25, 118.88 (^2^*J_CF_* = 26.34 Hz), 121.28 (^3^*J_CF_* = 8.54 Hz), 127.76, 129.55, 131.62, 136.30, 145.82, 152.82, 157.42, 158.78 (^1^*J_CF_* = 238.19 Hz). HRMS (*m*/*z*): [M + H]^+^ calcd for C_19_H_15_FN_2_O: 307.1241; found: 307.1235.

*1-(4-((4-Fluorophenyl)thio)benzylidene)-2-phenylhydrazine* (**2h**). Yield: 80%, M.P. = 132.3–133.9 °C, FTIR (ATR, cm^−1^): 3325 (N-H), 2958 (C-H), 1257 (C-N), 831, 752. ^1^H-NMR: δ = 6.75 (1H, t, *J =* 7.23 Hz, monosubstituted benzene H_4_), 7.06 (2H, d, *J* = 8.00 Hz, monosubstituted benzene H_2,2’_), 7.20 (2H, d, *J =* 7.32 Hz, disubstituted benzene CH), 7.23–7.29 (4H, m, monosubstituted benzene H_3,3’,_ fluorophenyl CH), 7.42–7.47 (2H, m, fluorophenyl CH), 7.62 (2H, d, *J =* 8.00 Hz, disubstituted benzene CH), 7.82 (1H, s, -CH=N-), 10.40 (1H, s, NH). ^13^C-NMR: δ = 112.49, 117.08, 117.32, 119.36, 126.99, 129.59, 130.42 (^2^*J_CF_* = 26.74 Hz), 134.33 (^3^*J_CF_* = 8.34 Hz), 135.20, 135.34, 135.87, 145.56, 162.29 (^1^*J_CF_* = 244.11 Hz). HRMS (*m*/*z*): [M + H]^+^ calcd for C_19_H_15_FN_2_S: 323.1013; found: 323.1001.

*1-(4-(1-Imidazolyl)benzylidene)-2-phenylhydrazine* (**2i**). Yield: 85%, M.P. = 201.9–208.9 °C, FTIR (ATR, cm^−1^): 3350 (N-H), 2951 (C-H), 1269 (C-N), 906, 759. ^1^H-NMR: δ = 6.77 (1H, t, *J =* 7.17 Hz, monosubstituted benzene H_4_), 7.12 (2H, d, *J =* 7.50 Hz, monosubstituted benzene H_3,3’_), 7.22 (2H, t, *J =* 7.29 Hz, monosubstituted benzene H_2,2’_), 7.54 (1H, s, imidazole CH), 7.75 (2H, d, *J* = 8.76 Hz, disubstituted benzene CH), 7.82 (2H, d, *J =* 8.76 Hz, disubstituted benzene CH), 7.95 (1H, s, -CH=N-) 8.07 (1H, m, imidazole CH), 9.08 (1H, s, imidazole CH), 10.68 (1H, s, NH). ^13^C-NMR: δ = 112.61, 119.48, 119.80, 121.84, 125.67, 127.19, 129.59, 135.26, 135.33, 135.38, 136.33, 145.55. HRMS (*m*/*z*): [M + H]^+^ calcd for C_16_H_14_N_4_: 263.1291; found: 263.1295.

*1-(4-(1H-1,2,4-triazole-1-yl)benzylidene)-2-phenylhydrazine* (**2j**). Yield: 79%, M.P. = 127.4–132.2 °C, FTIR (ATR, cm^−1^): 3319 (N-H), 2951 (C-H), 1228 (C-N), 829, 756. ^1^H-NMR: δ = 6.77 (1H, t, *J* = 7.23 Hz, monosubstituted benzene H4), 7.10 (2H, d, *J* = 7.53 Hz, monosubstituted benzene H_3,3’_), 7.23 (2H, t, *J* = 7.26 Hz, monosubstituted benzene H_2,2’_), 7.26 (1H, s, triazole CH), 7.81 (2H, d, *J* = 8.76 Hz, disubstituted benzene CH), 7.88 (2H, d, *J* = 9.15 Hz, disubstituted benzene CH), 8.25 (1H, s, -CH=N-), 9.33 (1H, s, triazole CH), 10.47 (1H, s, NH). ^13^C-NMR: δ =112.61, 119.48, 119.80, 121.84, 125.67, 127.19, 129.59, 135.26, 135.38, 136.33, 145.55. HRMS (*m*/*z*): [M + H]^+^ calcd for C_15_H_13_N_5_: 264.1244; found: 264.1230.

*1-(4-((4-Chlorophenyl)thio)benzylidene)-2-phenylhydrazine* (**2k**). Yield: 85%, M.P. = 164.4–165.7 °C, FTIR (ATR, cm^−1^): 3223 (N-H), 2912 (C-H), 1255 (C-N), 827, 744. ^1^H-NMR: δ = 6.76 (1H, t, *J =* 7.26 Hz, monosubstituted benzene H4), 7.07 (2H, d, *J =* 7.59 Hz, monosubstituted benzene H_2,2’_), 7.19–7.24 (2H, m, monosubstituted benzene H_3,3’_), 7.32 (2H, d, *J* = 8.64 Hz, disubstituted benzene CH), 7.36 (2H, d, *J =* 8.40 Hz, disubstituted benzene CH), 7.43 (2H, d, *J =* 8.64 Hz, disubstituted benzene CH), 7.66 (2H, d, *J =* 8.40 Hz, disubstituted benzene CH), 7.85 (1H, s, -CH=N-), 10.43 (1H, s, NH). ^13^C-NMR: δ = 112.55, 119.44, 127.13, 129.59, 129.59, 129.96, 132.12, 132.18, 132.40, 133.38, 134.94, 135.76, 136.07, 145.53. ESI-MS (M+H): C_19_H_15_ClN_2_S: 339.10.

*1-(4-(Benzylpiperidine)benzylidene)-2-phenylhydrazine* (**2l**). Yield: 85%, M.P. = 175.4–178.3 °C, FTIR (ATR, cm^−1^): 3223 (N-H), 2816 (C-H), 1247 (C-N), 817, 740. ^1^H-NMR: δ = 1.24–1.28 (2H, m, piperidine), 1.61–1.65 (3H, m, piperidine), 2.51–2.54 (2H, m, piperidine), 2.59–2.67 (2H, m, piperidine), 3.70–3.74 (2H, d, *J =* 12.57 Hz, -CH_2_-), 6.69 (1H, t, *J =* 7.23 Hz, monosubstituted benzene H4), 6.90 (2H, d, *J =* 8.85 Hz, disubstituted benzene CH), 7.02 (2H, d, *J =* 7.56 Hz, monosubstituted benzene H_2,2’_), 7.16–7.21 (5H, m), 7.26–7.31 (2H, m), 7.46 (2H, d, *J =* 8.79 Hz, disubstituted benzene CH), 7.77 (1H, s, -CH=N-), 9.99 (1H, s, NH). ^13^C-NMR): δ = 31.66, 37.74, 42.73, 48.71, 112.17, 115.71, 118.51, 126.13, 126.24, 127.16, 128.61, 129.48, 129.49, 137.77, 140.66, 146.21, 151.44. ESI-MS (M + H): C_25_H_27_N_3_: 370.30.

*1-(4-(2-Dimethylaminoethyl)piperazine)benzylidene)-2-phenylhydrazine* (**2m**). Yield: 87%, M.P. = 140.7–143.7 °C, FTIR (ATR, cm^−1^): 3223 (N-H), 2823 (C-H), 1253 (C-N), 821, 744. ^1^H-NMR: δ = 2.15 (6H, s, -CH_3_), 2.35-2.39 (2H, m, -CH_2_-), 2.41-2.46 (2H, m, -CH_2_-), 2.51–2.55 (4H, m, piperazine), 3.15–3.18 (4H, m, piperazine), 6.69 (1H, t, *J* = 7.23 Hz, monosubstituted benzene H4), 6.93 (2H, d, *J =* 8.76 Hz, disubstituted benzene CH), 7.01 (2H, d, *J =* 7.65 Hz, monosubstituted benzene H_2,2’_), 7.18 (2H, t, *J =* 7.82 Hz, monosubstituted benzene H_3,3’_), 7.48 (2H, d, *J =* 8.76 Hz, disubstituted benzene CH), 7.77 (1H, s, -CH=N-), 10.01 (1H, s, NH). ^13^C-NMR: δ = 46.04, 48.24, 53.47, 56.34, 57.15, 112.19, 115.41, 118.57, 126.65, 127.12, 129.50, 137.68, 146.18, 151.29. ESI-MS (M + H): C_21_H_29_N_5_: 352.35.

*1-(4-(3-Dimethylaminopropyl)piperazine)benzylidene)-2-phenylhydrazine* (**2n**). Yield: 84%, M.P. = 142.6–145.5 °C, FTIR (ATR, cm^−1^): 3217 (N-H), 2823 (C-H), 1267 (C-N), 821, 744. ^1^H-NMR: δ = 1.59 (2H, p, *J =* 7.56 Hz, -CH_2_-), 2.15 (6H, s, -CH_3_), 2.22–2.35 (4H, m, -CH_2_-), 2.47-2.49 (4H, m, piperazine), 3.16–3.19 (4H, m, piperazine), 6.69 (1H, t, *J =* 7.23 Hz, monosubstituted benzene H4), 6.93 (2H, d, *J =* 8.76 Hz, disubstituted benzene CH), 7.01 (2H, d, *J =* 7.59 Hz, monosubstituted benzene H_2,2’_), 7.18 (2H, t, *J =* 7.38 Hz, monosubstituted benzene H3,3’), 7.48 (2H, d, *J =* 8.76 Hz, disubstituted benzene CH), 7.78 (1H, s, -CH=N-), 10.04 (1H, s, NH). ^13^C-NMR: δ = 24.75, 45.50, 48.22, 53.16, 56.41, 57.67, 112.18, 115.41, 118.54, 126.66, 127.11, 129.48, 137.65, 146.17, 151.27. ESI-MS (M + H): C_22_H_31_N_5_: 366.35.

### 3.3. Activity Studies

#### 3.3.1. MAO-A and MAO-B Inhibition Assay

Ampliflu™ Red (10-Acetyl-3,7-dihydroxyphenoxazine), peroxidase from horseradish, *h*MAO-A, *h*MAO-B, H_2_O_2_, tyramine hydrochloride, selegiline and moclobemide were purchased from Sigma-Aldrich (Steinheim, Germany) and retained under the suggested conditions by supplier. All pipetting processes were performed using a Biotek Precision XS robotic system (BioTek Instruments, Winooski, VT, USA). Measurements were carried out by a BioTek-Synergy H1 microplate reader based on the fluorescence generated (excitation, 535 nm, emission, 587 nm) over a 30 min period, in which the fluorescence increased linearly.

In the enzymatic assay, three different daily prepared solutions were used. (I) Inhibitor solutions: Synthesized compounds and reference agents were prepared in 2% DMSO in 10^−3^–10^−9^ M concentrations (10 mL for each concentration). (II) Enzyme solutions: Recombinant *h*MAO-A (0.5 U/mL) and recombinant *h*MAO-B (0.64 U/mL) enzymes were dissolved in the phosphate buffer and final volumes were adjusted to 10 mL. (III) Working solution: Horseradish peroxidase (200 U/mL, 100 μL), Ampliflu™ Red (20 mM, 200 μL) and tyramine (100 mM, 200 μL) were dissolved in the phosphate buffer and final volume was adjusted to 10 mL.

The solutions of inhibitor (20 μL/well) and *h*MAO-A (100 μL/well) or *h*MAO-B (100 μL/well) were added to the flat black bottom 96-well micro test plate, and incubated at 37 °C for 30 min. After this incubation period, the reaction was started by adding a working solution (100 μL/well). The mixture was incubated at 37 °C for 30 min and the fluorescence (Ex/Em = 535/587 nm) was measured at 5 min intervals. Control experiments were carried out simultaneously by replacing the inhibitor solution with 2% DMSO (20 μL). To check the probable inhibitory effect of inhibitors on horseradish peroxidase, a parallel reading was performed by replacing enzyme solutions with 3% H_2_O_2_ solution (20 mM 100 μL/well). In addition, the possible capacity of the inhibitors to modify the fluorescence generated in the reaction mixture due to non-enzymatic inhibition was determined by mixing inhibitor and working solutions.

The specific fluorescence emission (used to obtain the final results) was calculated after subtraction of the background activity, which was determined from vials containing all components except the *h*MAO isoforms, which were replaced by phosphate buffer (100 μL/well). Blank, control and all concentrations of inhibitors were analyzed in quadruplicate and inhibition percent was calculated by using following equation:

$$\%\;\mathrm{Inhibition}=\frac{\left({\mathrm{FC}}_{t2}-{\mathrm{FC}}_{t1}\right)-\left({\mathrm{FI}}_{t2}-{\mathrm{FI}}_{t1}\right)}{{\mathrm{FC}}_{t2}-{\mathrm{FC}}_{t1}}\times 100$$

where FCt_2_: Fluorescence of a control well measured at t_2_ time, FCt_1_: Fluorescence of a control well measured at t_1_ time, FIt_2_: Fluorescence of an inhibitor well measured at t_2_ time, FIt_1_: Fluorescence of an inhibitor well measured at t_1_ time. The IC_50_ values were calculated from a dose-response curve obtained by plotting the percentage inhibition versus the log concentration with the use of GraphPad “PRISM” software (version 5.0, GraphPad Software Inc., La Jolla, CA, USA). The results were displayed as mean ± standard deviation (SD).

#### 3.3.2. Enzyme Kinetics Studies

The same materials were used as in the MAO inhibition assay. The most active compounds **2a** and **2b** were tested at three different concentrations (IC_50_/2, IC_50_ and 2 × IC_50_). The solutions of inhibitor (20 μL/well) and enzyme were added to the flat black bottom 96-well micro test plate, and incubated at 37 °C for 30 min. After incubation period, the working solution, including various concentrations (20, 10, 5, 2.5, 1.25, and 0.625 μM) of tyramine (100 μL/well) was added. The increase of the fluorescence (E_x_/E_m_ = 535/587 nm) was recorded for 30 min. A parallel experiment was carried out without inhibitor. All processes were assayed in quadruplicate. The results were analyzed as Lineweaver-Burk plots using Microsoft Office Excel 2013. The K_m_/V_max_ (slope) values of the Lineweaver-Burk plots were replotted versus the inhibitor concentration, and the *K_i_* values were determined from the x-axis intercept as −K*_i_*.

### 3.4. Toxicology Studies

#### 3.4.1. Cytotoxicity Test

In cytotoxicity test by using NIH/3T3 mouse embryonic fibroblast cell line (ATCC^®^ CRL-1658™, London, UK), firstly NIH/3T3 cells were incubated in the Dulbecco’s Modified Eagle’s Medium (DMEM, Sigma Aldrich, St. Louis, MO, USA). NIH/3T3 cells were plated on 96-well culture plates as 10,000 cells per well and were then treated with the compounds at concentrations ranging from 1000 µM to 0.316 µM (1000, 316, 100, 31.6, 10, 3.16, 1, 0.316 µM). MTT assay was performed as previously described [35,36,37,38]. Dose-response curves were plotted against compound concentrations applied to determine IC_50_ values. The following formula was used to calculate the inhibition percentage for each concentration. % inhibition = 100 − (mean sample × 100/mean solvent).

#### 3.4.2. Genotoxicity Test

The genotoxicity of the compounds was determined by Ames assay as previously described using the Ames MPF 98/100 mutagenicity test sample kit (Xenometrix AG, Allschwil, Switzerland). This test was performed with *Salmonella typhimurium* strains TA98 (frameshift mutations) and TA100 (base-pair substitutions). Compounds was prepared in six different concentrations (5, 2.5, 1.25, 0.625, 0.3125, 0.156 mg/mL) which was between 16 and 5000 µg/mL according to the previous guidelines in DMSO. In order to detect mutagenic potential, test performed with and without Aroclor™-1254 induced male Sprague-Dawley rat liver microsomal enzyme (S9) mix (Xenometrix AG). While the positive control for TA 98 without S9 mix was 2-nitrofluorene (2 μg/mL), 1 μg/mL of 2-aminoanthracene was positive control with S9 against TA 98. For TA100, 4-nitroquinoline N-oxide (0.1 μg/mL) was positive control without S9 mix and 2.5 μg/mL of 2-aminoanthracene was positive control. 4% DMSO solution was the solvent control. At the end of the experiment, revertant bacteria decreased the pH of solution and indicator medium colour was changed to yellow. Yellow wells were counted as positive and compared with the negative control. Fold induction over the negative control and fold induction over the baseline were calculated. Fold induction over the negative control is the ratio of the mean number of positive wells for the dose concentration divided by the mean number of positive wells for the zero dose (negative) control. Fold induction over the baseline is the ratio of the mean number of positive wells for the dose concentration divided by zero dose baseline. The zero dose baseline is obtained by adding one standard deviation to the mean number of positive wells of the zero dose control [36,37]. Mutagenity was determined according to the criteria from previous studies [25]. For a baseline value ≤ 3, significant increases between 2 and 3-fold the baseline were classified as weak mutagen, increases ≥ 3-fold the baseline were classified as mutagen. For a baseline was > 3, significant increases between 1.5 and 2.5-fold the baseline were classified as weak mutagen, and increases ≥ 2.5-fold the baseline, were classified as mutagen. In principle, for a mutagenic compound, at least two adjacent doses with significant increase or a significant increase at the highest dose level should be observed. All doses were compared according to Student’s t-test at *p* < 0.05 as statistically. When compounds did not have any of the properties referred above, they were classified as a non-mutagenic compound.

### 3.5. Prediction of ADME Parameters and BBB Permeability

Physicochemical parameters of compounds **2a**–**2n** were analyzed by online Molinspiration property calculation program [27]. BBB permeability of the compounds was assigned by an online BBB Predictor [29].

### 3.6. Molecular Docking Studies

A structure based in silico procedure was applied to discover the binding modes of moclobemide and compound **2b** to *h*MAO-A enzyme active site. The crystal structures of *h*MAO-A (PDB ID: 2Z5X) [30], which was crystallized with the reversible inhibitor harmine, was retrieved from the Protein Data Bank server (www.pdb.org). The structures of ligands were built using the *Schrödinger Maestro* [39] interface and then were submitted to the Protein Preparation Wizard protocol of the Schrödinger Suite 2016 Update 2 [40]. The ligands were prepared by the LigPrep 3.8 [41] to assign the protonation states at pH 7.4 ± 1.0 and the atom types, correctly. Bond orders were assigned and hydrogen atoms were added to the structures. The grid generation was formed using Glide 7.1 [42]. The grid box with dimensions of 20 Å × 20 Å × 20 Å was centered in the vicinity of the flavin (FAD) N5 atom on the catalytic site of the protein to cover all binding sites and neighboring residues [43,44]. Flexible docking runs were performed with single precision docking mode (SP).

## 4. Conclusions

Despite their significant potency, hydrazine type irreversible MAO inhibitors are not desirable in the treatment of neurological disorders due to their side effects. It may be possible to eliminate such side effects by the development of a new class of compounds with reversible enzyme inhibition. From this point of view, the synthesis, enzyme inhibition, enzyme kinetics, preliminary toxicological screening, ADME prediction and docking evaluations of new hydrazone derivatives were undertaken in the current study. Enzymatic studies revealed the potency of compounds **2a**, **2b** as selective, reversible and competitive *h*MAOA inhibitors. Toxicological and ADME studies highlighted the biological importance of these compounds. Docking assessments clearly demonstrated the binding modes of these compounds to enzyme active site. Accordingly, all these data may hopefully prompt medicinal chemists to design and synthesize more potent and safer *h*MAO-A inhibitors, which may be valuable for the treatment of patients with depression.

## Figures and Tables

**Table 1:** % Inhibition of compounds **2a**–**2n**, moclobemide and selegiline against MAO-A and MAO-B.

Compound	MAO-A Inhibition %		MAO-B Inhibition %	
	10^−3^ M	10^−4^ M	10^−3^ M	10^−4^ M
**2a**	88.198 ± 0.821	84.288 ± 0.641	35.011 ± 0.766	22.150 ± 0.443
**2b**	93.530 ± 0.412	88.862 ± 0.214	63.747 ± 1.028	42.951 ± 1.203
**2c**	70.926 ± 1.064	43.245 ± 0.861	38.507 ± 0.732	34.740 ± 0.556
**2d**	68.645 ± 1.501	40.212 ± 0.970	39.226 ± 0.863	30.630 ± 0.613
**2e**	32.492 ± 0.844	25.018 ± 0.425	26.528 ± 0.478	19.401 ± 0.327
**2f**	30.522 ± 0.817	26.705 ± 0.748	33.302 ± 0.833	21.880 ± 0.416
**2g**	54.538 ± 1.003	20.380 ± 0.469	21.683 ± 0.520	17.080 ± 0.412
**2h**	28.552 ± 0.570	21.045 ± 0.548	30.210 ± 0.574	24.126 ± 0.355
**2i**	40.926 ± 0.900	18.326 ± 0.618	38.215 ± 1.095	30.718 ± 0.707
**2j**	38.645 ± 0.743	33.013 ± 0.660	27.511 ± 0.633	22.055 ± 0.419
**2k**	29.212 ± 0.672	21.526 ± 0.468	36.278 ± 0.774	18.978 ± 0.825
**2l**	74.251 ± 1.614	44.828 ± 0.917	45.194 ± 0.692	28.560 ± 0.629
**2m**	68.276 ± 1.048	39.471 ± 0.719	41.072 ± 0.732	27.607 ± 0.961
**2n**	64.435 ± 1.104	36.154 ± 0.817	40.629 ± 0.933	29.068 ± 0.734
**Moclobemide**	94.121 ± 2.760	82.143 ± 2.691	-	-
**Selegiline**	-	-	98.910 ± 1.280	96.882 ± 1.312

**Table 2:** IC_50_ values of **2a**, **2b** and moclobemide against MAO-A.

Compound	MAO-A Inhibition %							MAO-A IC_50_ (µM)
	10^−3^ M	10^−4^ M	10^−5^ M	10^−6^ M	10^−7^ M	10^−8^ M	10^−9^ M	
**2a**	88.198 ± 0.821	84.288 ± 0.641	75.098 ± 0.693	50.828 ± 0.582	34.748 ± 0.378	30.548 ± 0.542	25.458 ± 0.517	0.342 ± 0.015
**2b**	93.530 ± 0.412	88.862 ± 0.214	80.418 ± 0.470	66.218 ± 0.540	48.243 ± 0.631	45.273 ± 0.480	38.150 ± 0.367	0.028 ± 0.001
**Moclobemide**	94.121 ± 2.760	82.143 ± 2.691	60.458 ± 2.559	36.151 ± 1.984	22.135 ± 1.337	18.166 ± 0.812	14.128 ± 0.725	6.061 ± 0.262

**Table 3:** Cytotoxic activity of the compounds **2a** and **2b** against NIH/3T3 Cell Line.

Compound	IC_50_ (µM)
**2a**	930 ± 15.43
**2b**	20 ± 1.32

**Table 4:** The AMES MPF results of the compounds **2a** and **2b**.

Comp.	Concentration (mg/mL)	Revertants Fold Increase (Over Baseline)			
		TA 98		TA 100	
		S9−	S9+	S9−	S9+
**2a**	0.156	0.42	0.92	0.44	0.99
	0.3125	0.08 *	0.83	0.63	0.69
	0.625	0.58	0.58	0.24 *	0.91
	1.25	0.50	0.75	0.19 *	0.95
	2.5	0.08 *	0.75	0.05 *	0.39
	5	0.17 *	1.17	0.00 *	0.82
**2b**	0.156	1.24 *	0.85	0.40	1.46 *
	0.3125	0.36	2.44 *	0.17 *	1.40
	0.625	0.22	1.59 *	0.06 *	1.46
	1.25	0.00	0.37	0.03 *	0.79
	2.5	0.00	0.37	0.00 *	0.12 *
	5	0.00	0.12	0.00 *	0.00 *

* *t* test *p* value (unpaired 1-sided) < 0.05.

**Table 5:** Some physicochemical parameters of the compounds **2a**–**2n** and reference drugs used in prediction of ADME profiles.

Compound	MW	logP	TPSA	HBA	HBD	MV	Vio	BBB
**2a**	293.41	6.10	27.63	1	1	293.65	1	+
**2b**	294.40	4.76	105.11	2	1	289.61	0	+
**2c**	356.47	6.46	30.87	1	1	344.46	1	+
**2d**	386.50	6.51	40.10	2	1	370.00	1	+
**2e**	318.38	6.57	42.86	3	1	297.05	1	+
**2f**	334.44	6.79	33.62	3	1	306.20	1	+
**2g**	306.34	6.68	33.62	2	1	276.44	1	+
**2h**	322.41	6.89	24.39	1	1	285.58	1	+
**2i**	262.32	4.09	42.22	2	1	243.73	0	+
**2j**	263.30	3.56	55.11	3	1	239.58	0	+
**2k**	338.86	7.41	24.39	2	1	294.19	1	+
**2l**	369.51	7.44	27.63	3	1	365.30	1	+
**2m**	351.50	4.79	34.10	5	1	352.56	0	+
**2n**	365.52	5.06	34.10	5	1	369.36	1	+
**Moclobemide**	268.74	1.69	41.57	4	1	240.70	0	+
**Selegiline**	187.29	2.64	3.24	1	0	202.64	0	+

MW: Molecular weight, logP: Octanol/water partition coefficient, TPSA: Topological polar surface area, HBA: Number of hydrogen acceptors, HBD: Number of hydrogen donors, MV: Molecular volume, Vio: Number of violations, BBB: Blood brain barrier permeability.

## Supplementary Materials

The ^13^C-NMR, ^1^H-NMR, FTIR, and HRMS spectrums of compounds **2a**–**2n** are available online.
