# Supplementary material for: Synthesis of New Hydrazone Derivatives for MAO Enzymes Inhibitory Activity
Source: Molecules. 2017 Aug 20;22(8):1381. doi: 10.3390/molecules22081381 (PMC6152084; doi:10.3390/molecules22081381)
Supplement: Supplementary file 1 [file molecules-22-01381-s001.pdf]

DOPNALAB

Item	Value
Acquired Date&Time	5.10.2016 09:43:26
Acquired by	System Administrator
Filename	C:\Users\dopnalab\Desktop\derya\fa\fa-2a2.ispd
Spectrum name	fa-2a2
Sample name	fa_2a
Sample ID	
Option	
Comment	
No. of Scans	10
Resolution	4 [cm-1]
Apodization	Happ-Genzel

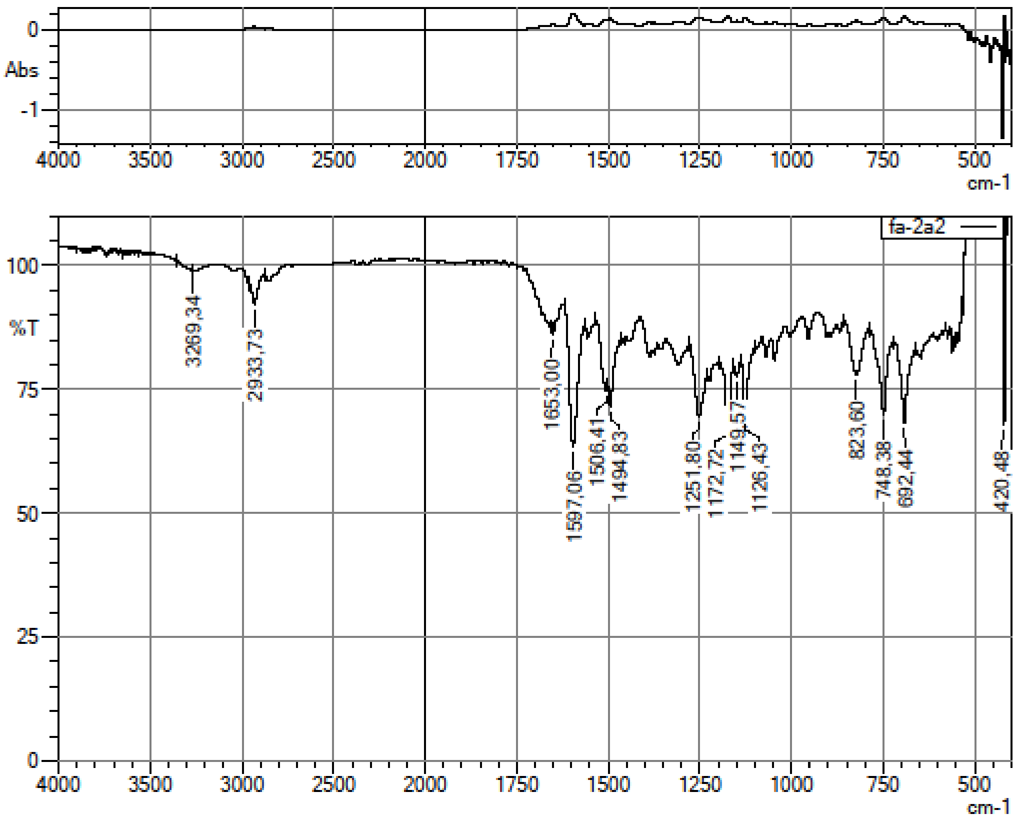

Spectra 1. IR spectra of the compound 2a

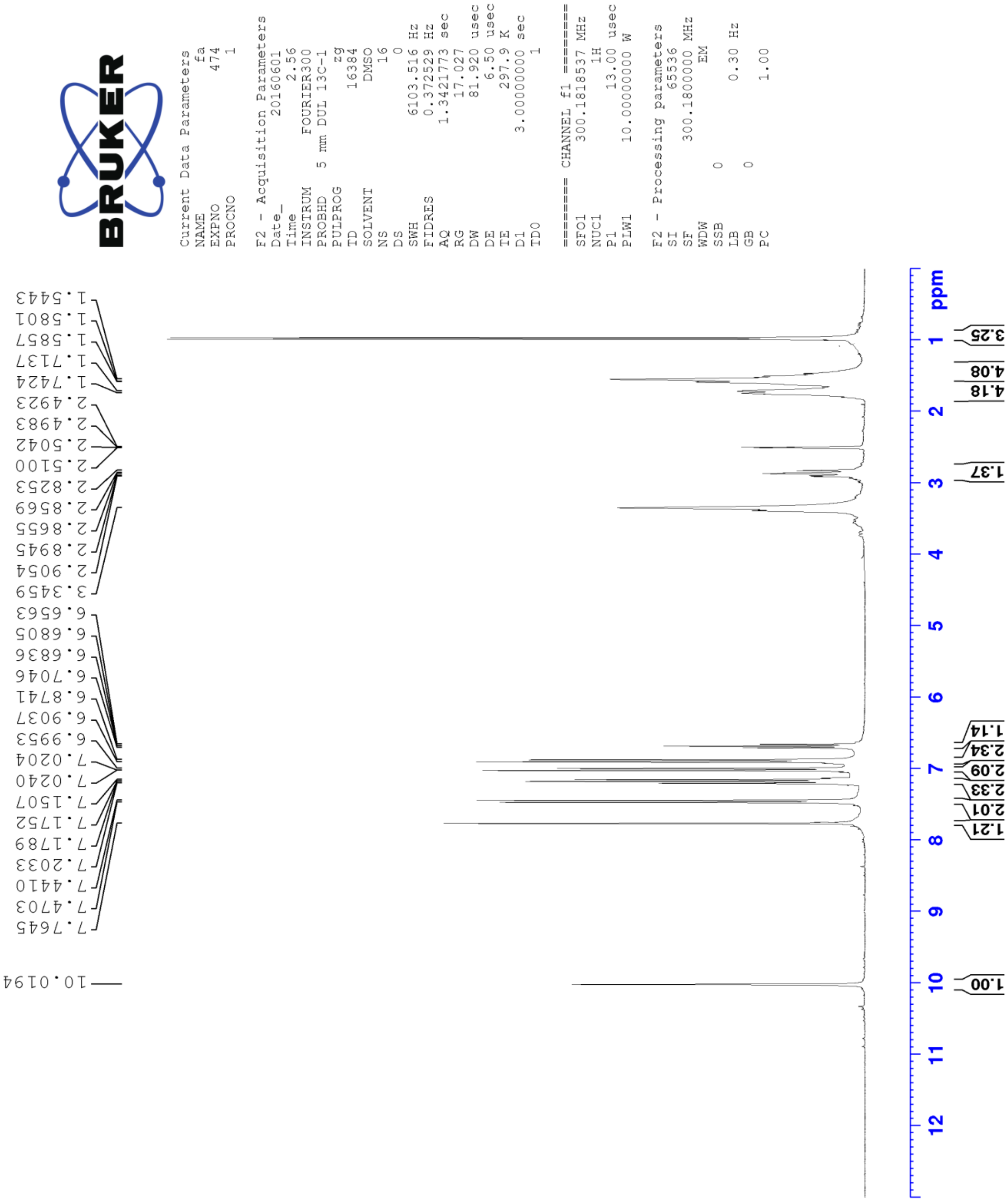

Spectra 2. ^{1}H NMR spectra of the compound 2a

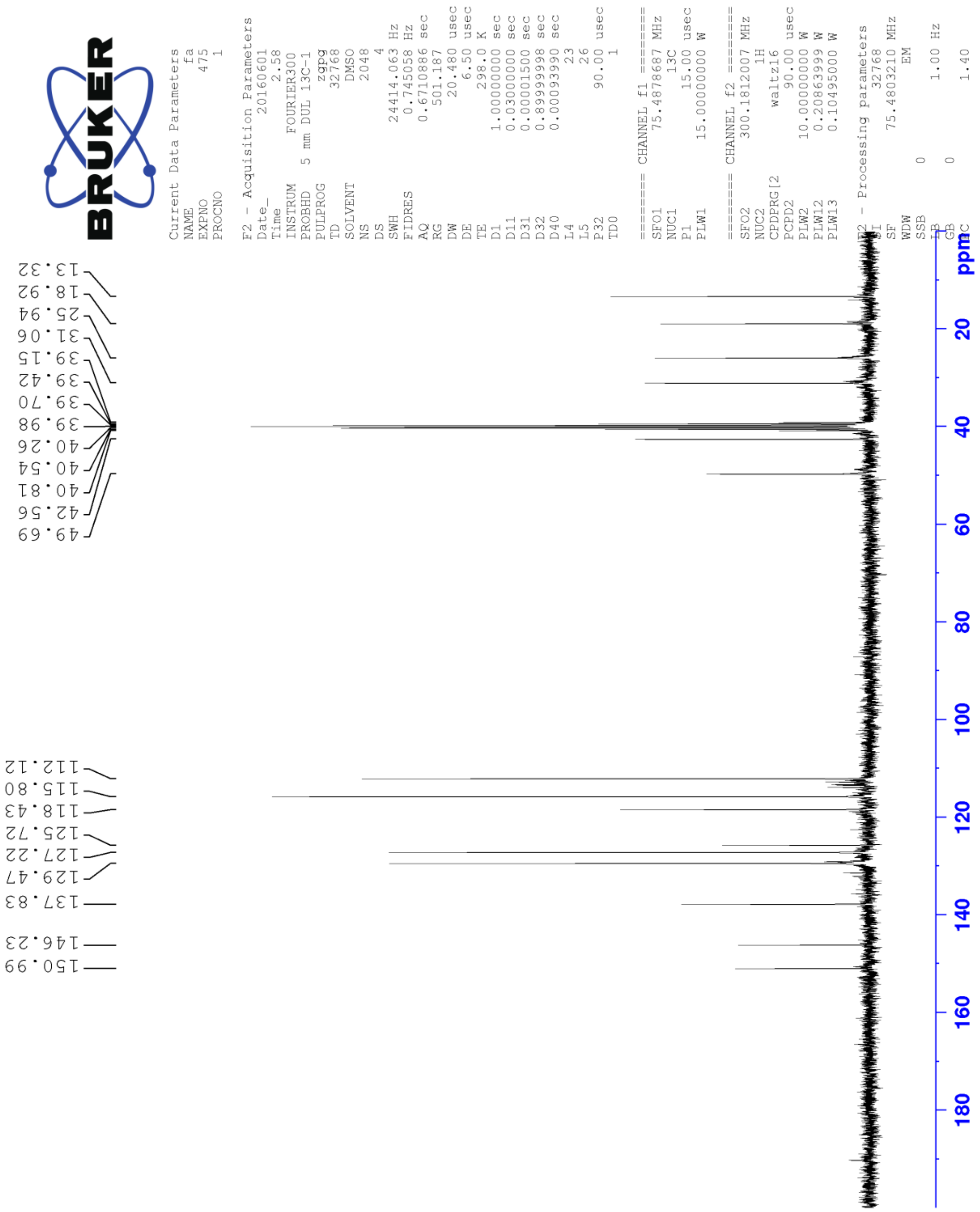

Spectra 3. ^{13}C NMR spectra of the compound 2a

Data File: C:\ LabSolutions\ Data\Analiz\derya\ FA-1_1.lcd

Elmt	Val.	Min	Max	Elmt	Val.	Min	Max	Elmt	Val.	Min	Max	Use Adduct
H	1	0	50	O	2	0	6	Cl	1	0	0	H
C	4	0	50	F	1	0	0	Br	1	0	0	
N	3	0	3	S	2	0	0	Pd	2	0	0	

Error Margin (ppm):	5	DBE Range:	10.0 - 20.0	Electron Ions:	both
HC Ratio:	unlimited	Apply N Rule:	yes	Use MSn Info:	no
Max Isotopes:	3	Isotope RI (%):	1.00	Isotope Res:	10000
MSn Iso RI (%):	10.00	MSn Logic Mode:	AND	Max Results:	500

Event#: 1 MS(E+) Ret. Time : 0.960 -> 1.107 Scan# : 145 -> 167

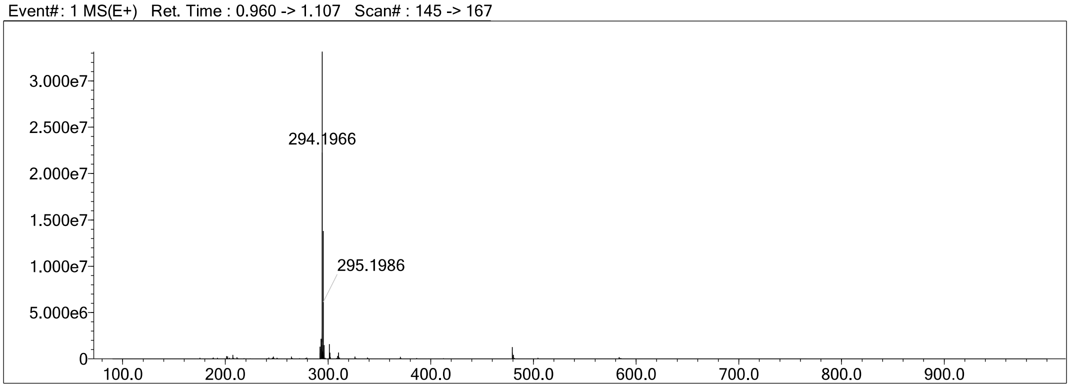

Measured region for 294.1966 m/ z

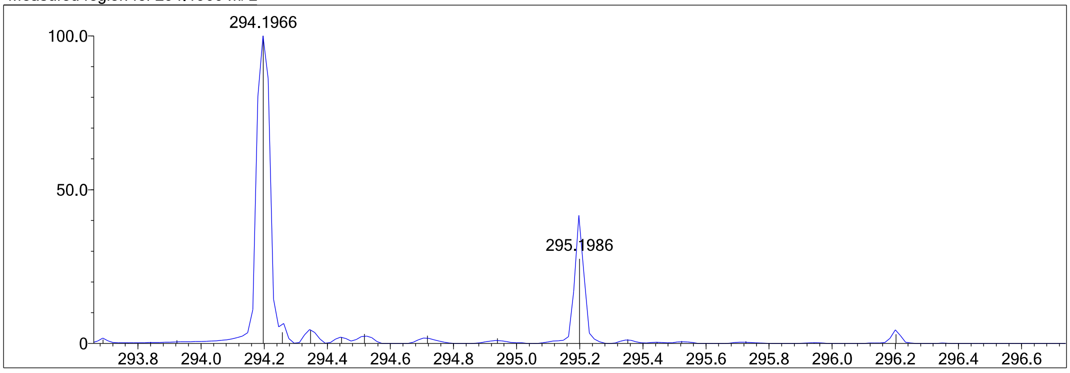

C19 H23 N3 [M+H]+ : Predicted region for 294.1965 m/ z

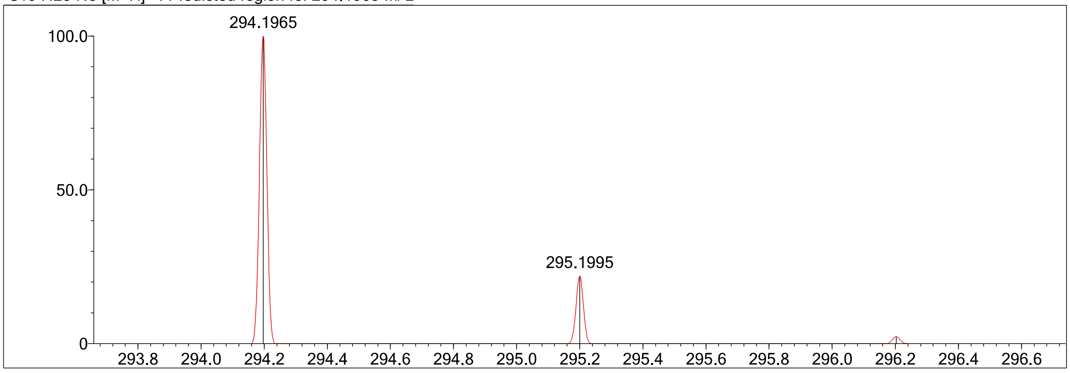

Rank	Score	Formula (M)	Ion	Meas. m/ z	Pred. m/ z	Df. (mDa)	Df. (ppm)	Iso	DBE
1	68.06	C19 H23 N3	[M+H]+	294.1966	294.1965	0.1	0.34	68.06	10.0

Spectra 4. HRMS spectra of the compound 2a

DOPNALAB

	Item	Value
	Acquired Date&Time	5.10.2016 09:48:36
	Acquired by	System Administrator
	Filename	C:\Users\dopnalab\Desktop\derya\fa\fa-2b1.ispd
	Spectrum name	fa-2b1
	Sample name	fa_2b
	Sample ID	
	Option	
	Comment	
	No. of Scans	10
	Resolution	4 [cm-1]
	Apodization	Happ-Genzel

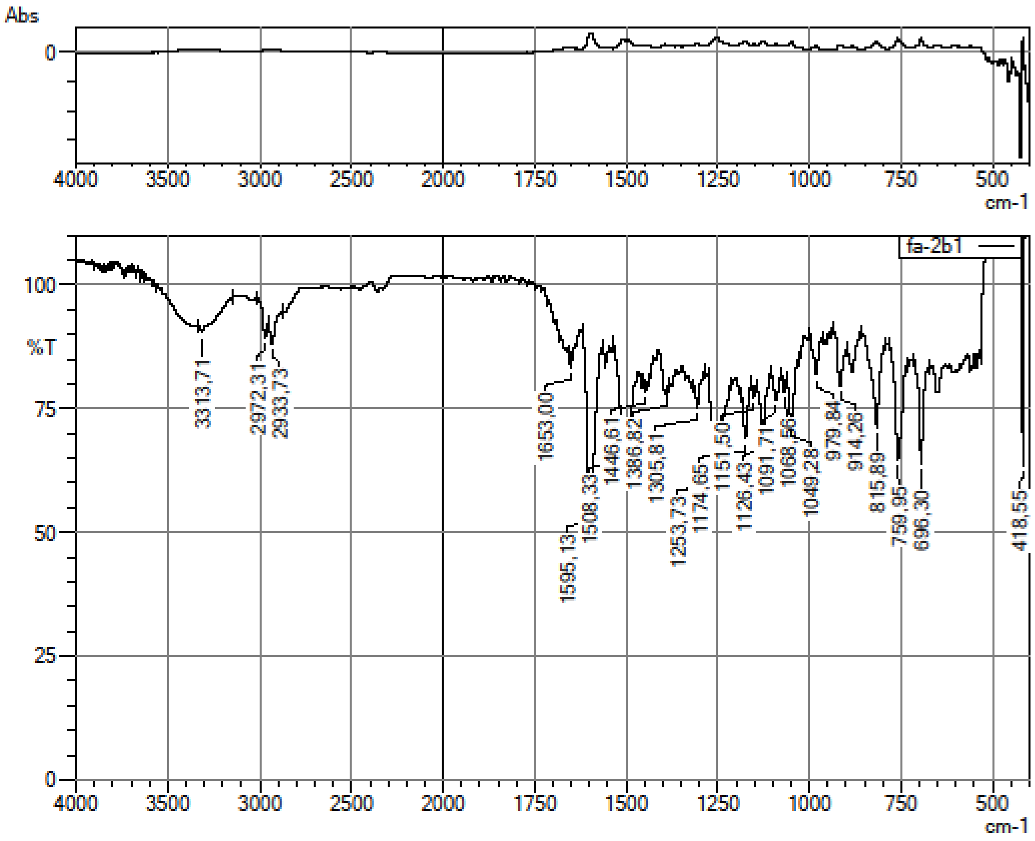

Spectra 5. IR spectra of the compound 2b

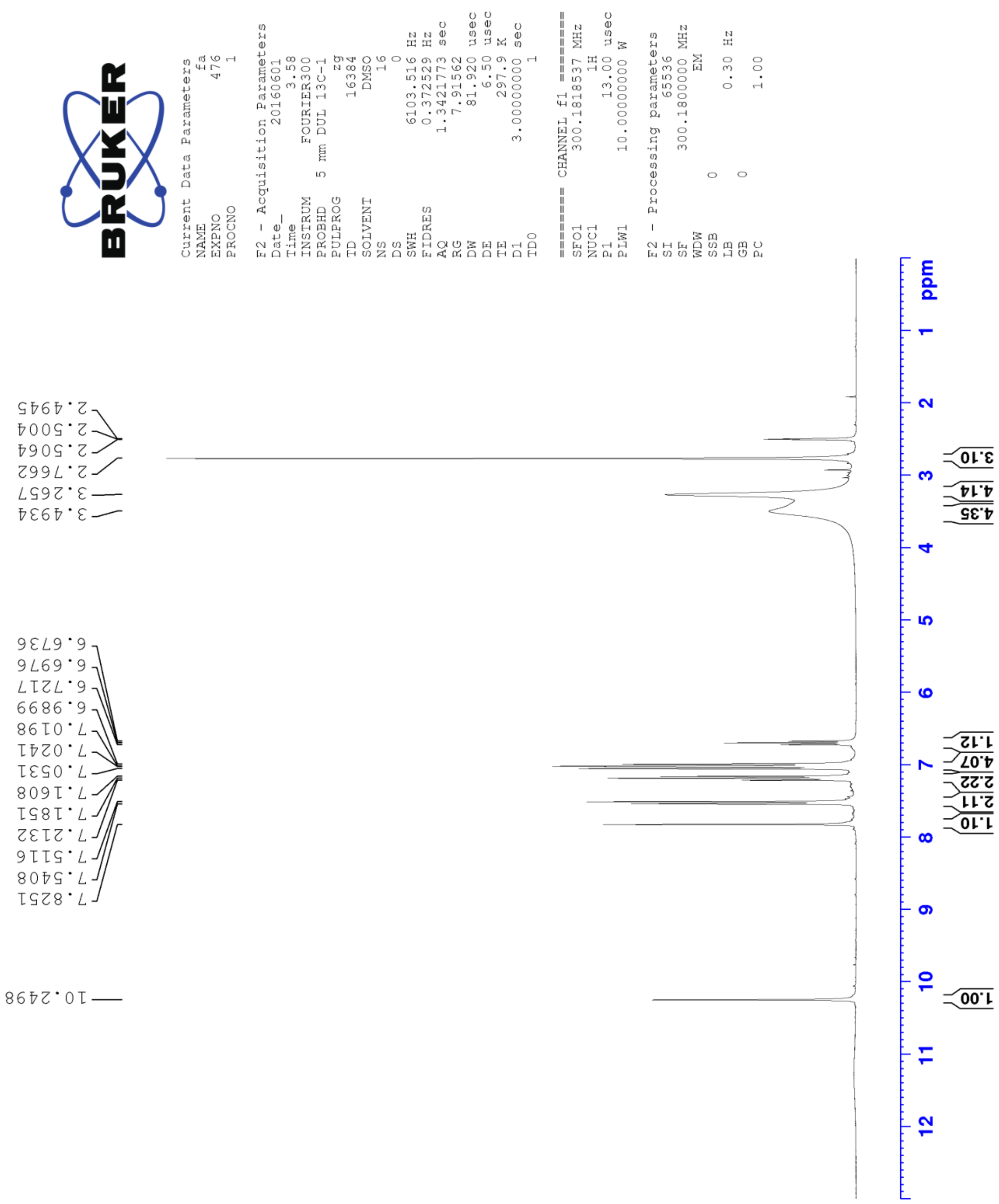

Spectra 6. ^{1}H NMR spectra of the compound 2b

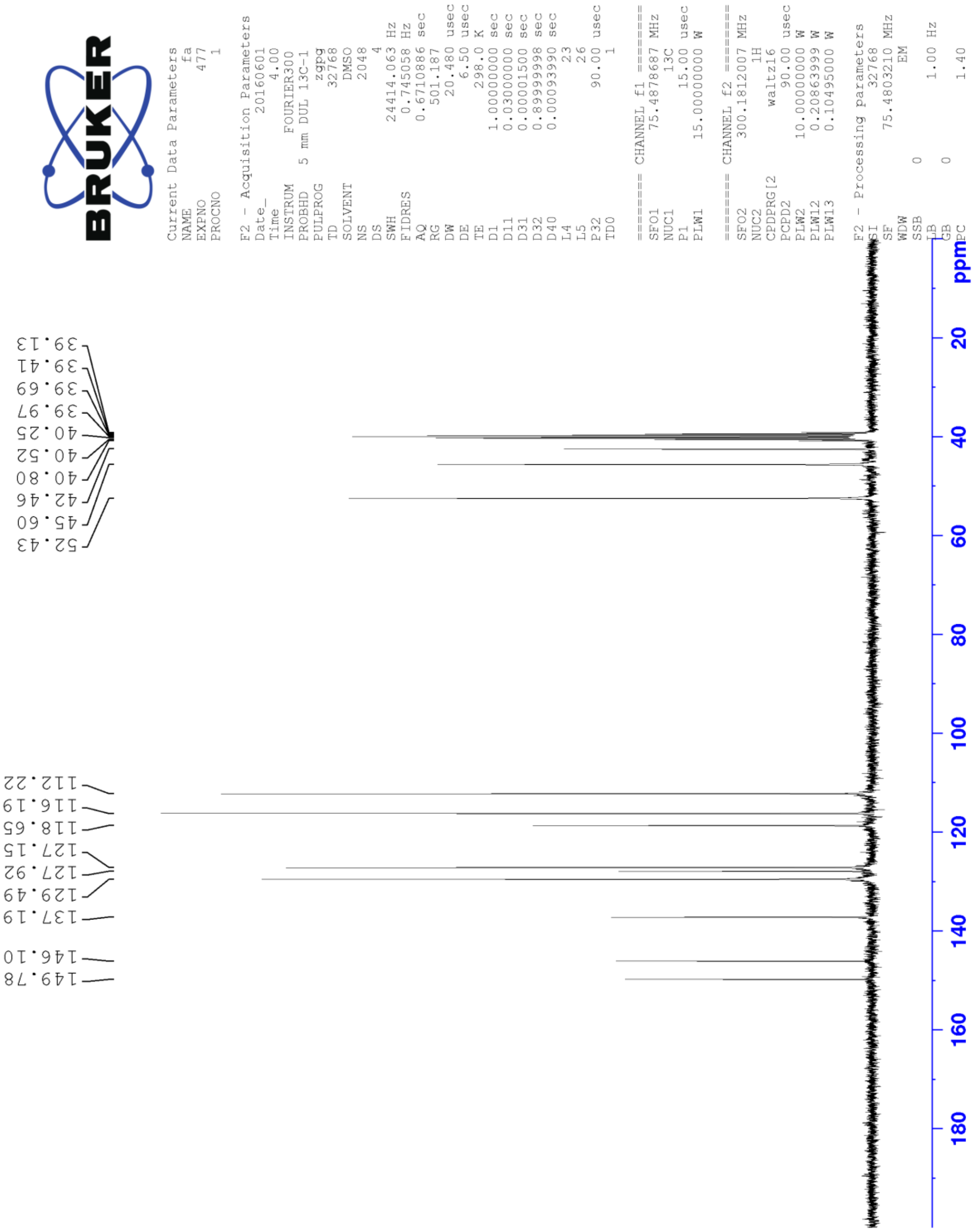

Spectra 7. ^{13}C NMR spectra of the compound 2b

Data File: C:\ LabSolutions\ Data\Analiz\derya\ FA-2_2.lcd

Elmt	Val.	Min	Max	Elmt	Val.	Min	Max	Elmt	Val.	Min	Max	Use Adduct
H	1	0	50	O	2	0	6	Cl	1	0	0	H
C	4	0	50	F	1	0	0	Br	1	0	0	
N	3	0	4	S	2	0	0	Pd	2	0	0	

Error Margin (ppm):	5	DBE Range:	10.0 - 20.0	Electron Ions:	both
HC Ratio:	unlimited	Apply N Rule:	yes	Use MSn Info:	no
Max Isotopes:	3	Isotope RI (%):	1.00	Isotope Res:	10000
MSn Iso RI (%):	10.00	MSn Logic Mode:	AND	Max Results:	500

Event#: 1 MS(E+) Ret. Time : 4.347 -> 4.413 Scan# : 653 -> 663

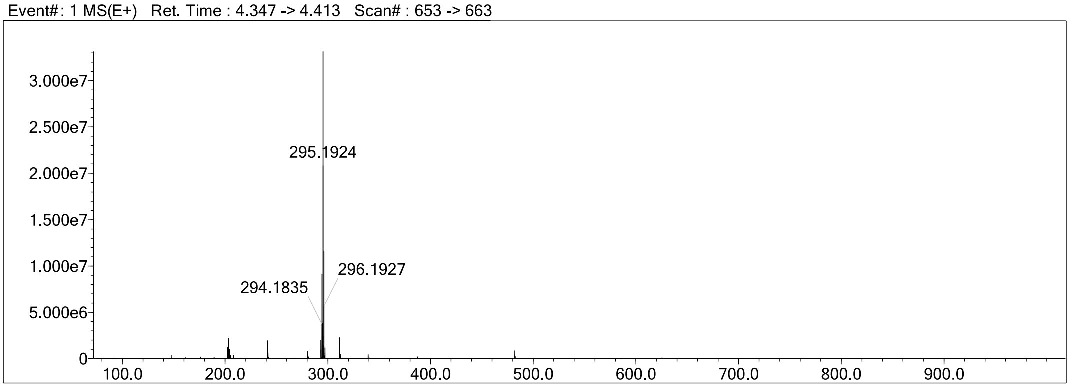

Measured region for 295.1924 m/z

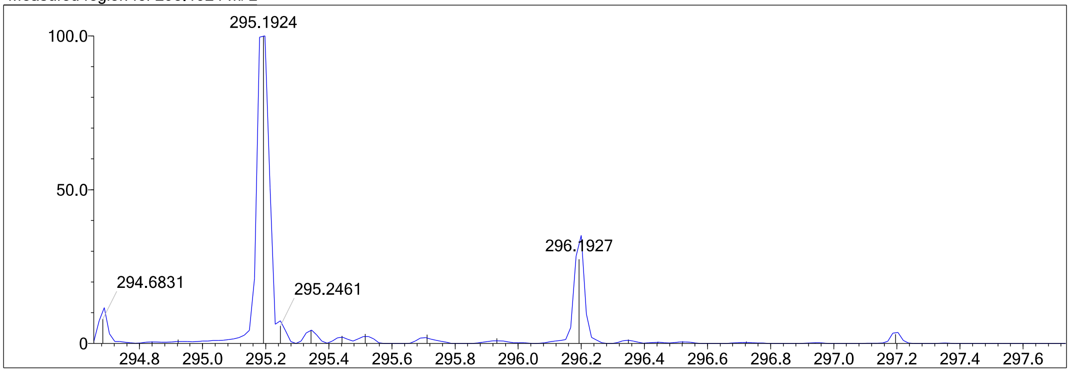

C18 H22 N4 [M+H]+ : Predicted region for 295.1917 m/z

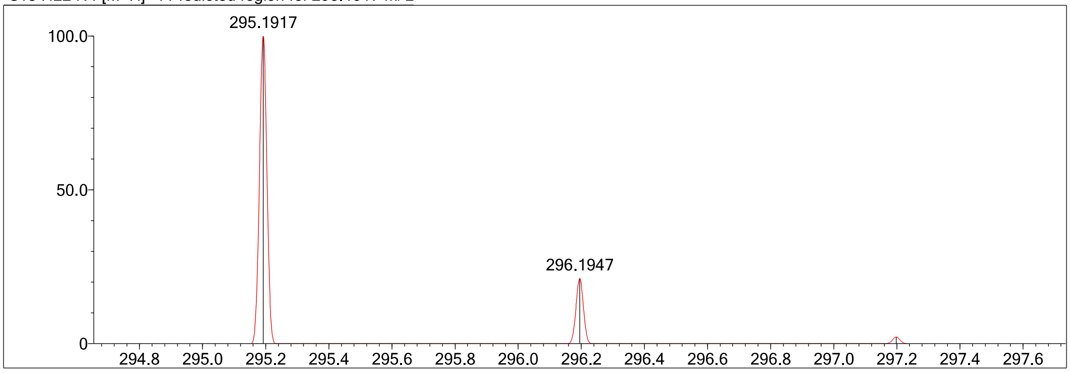

Rank	Score	Formula (M)	Ion	Meas. m/z	Pred. m/z	Df. (mDa)	Df. (ppm)	Iso	DBE
1	64.39	C18 H22 N4	[M+H]+	295.1924	295.1917	0.7	2.37	66.68	10.0

Spectra 8. HRMS spectra of the compound 2b

DOPNALAB

Item	Value
Acquired Date&Time	5.10.2016 09:53:15
Acquired by	System Administrator
Filename	C:\Users\dopnalab\Desktop\derya\fa\fa-2c1.ispd
Spectrum name	fa-2c1
Sample name	fa_2c
Sample ID	
Option	
Comment	
No. of Scans	10
Resolution	4 [cm-1]
Apodization	Happ-Genzel

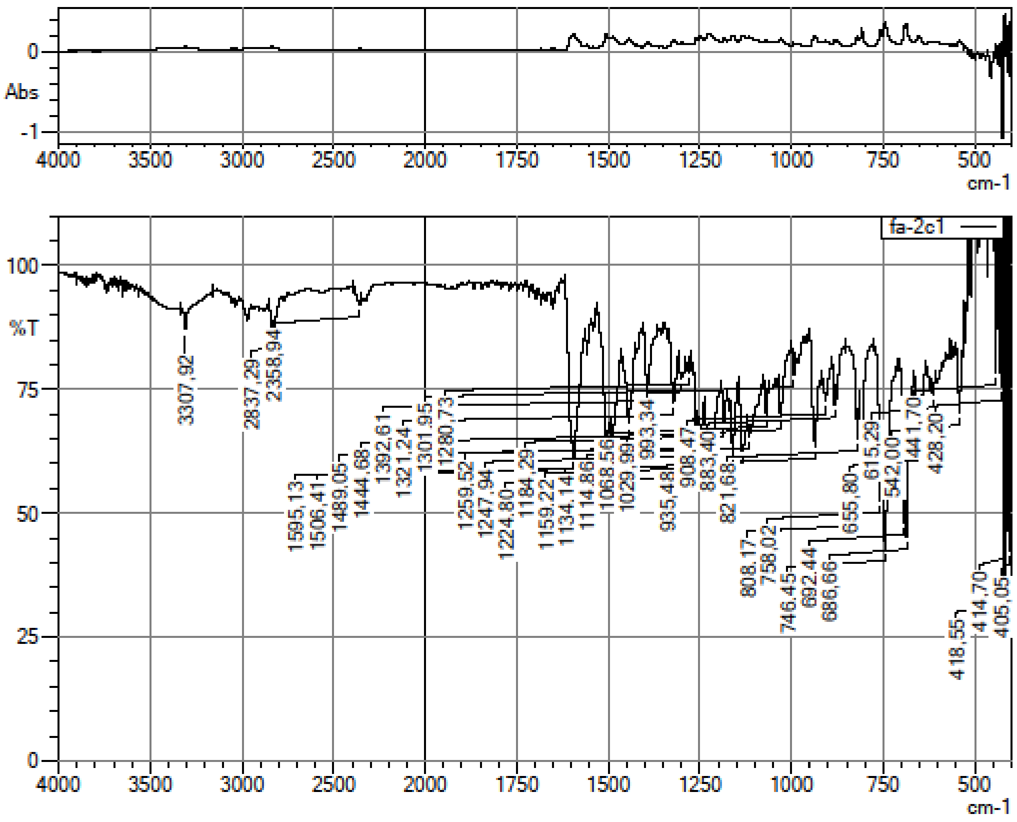

Spectra 9. IR spectra of the compound 2c

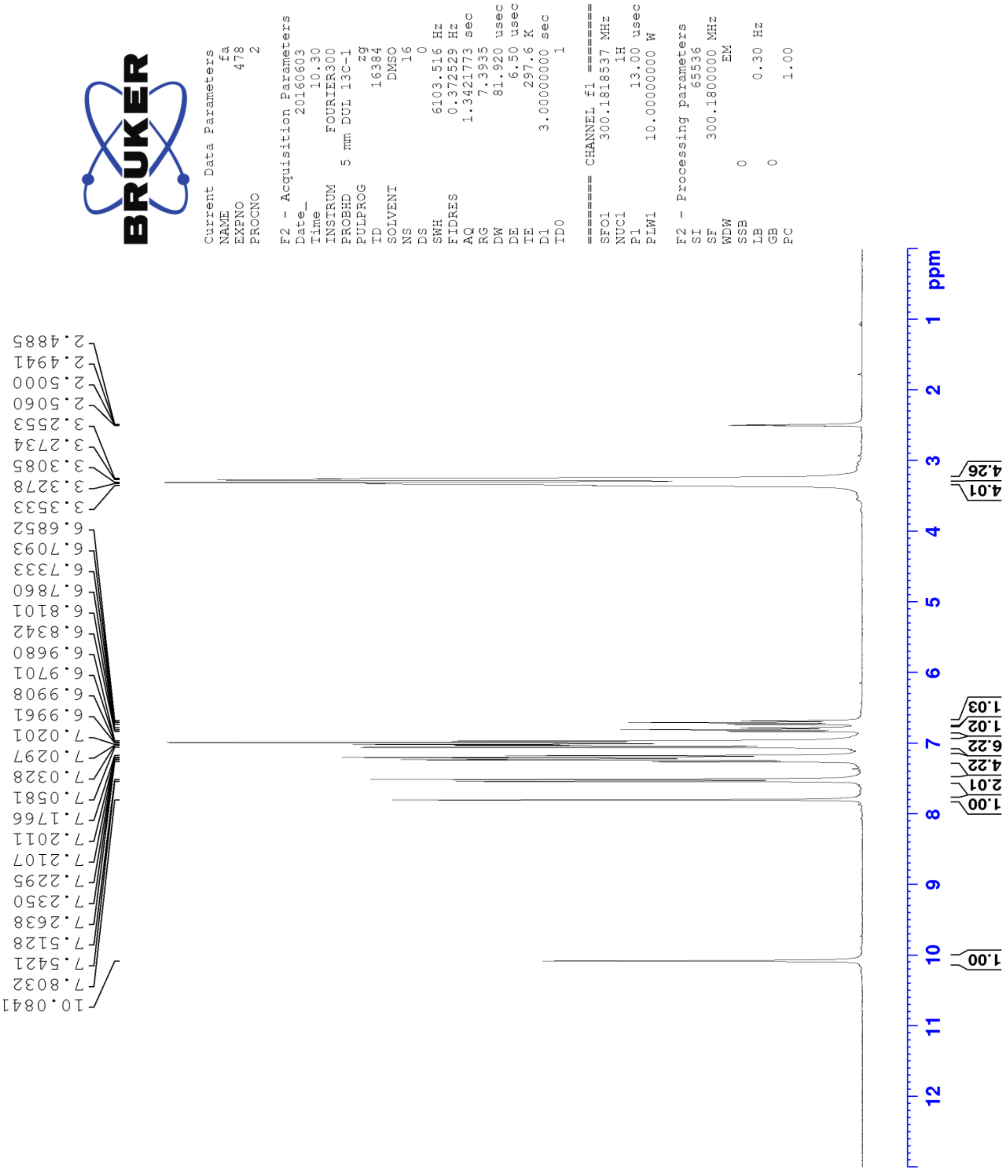

Spectra 10. ^{1}H NMR spectra of the compound 2c

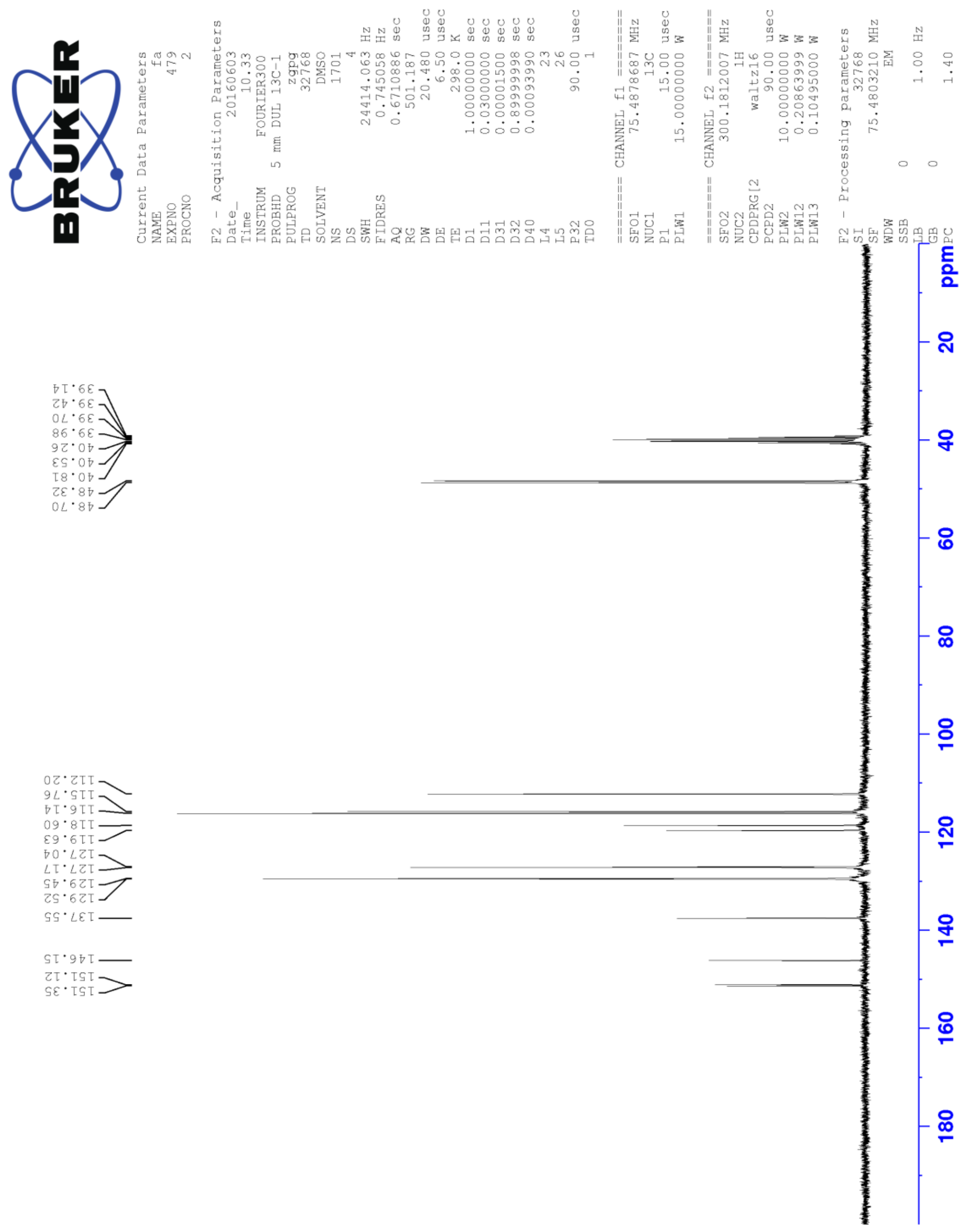

Spectra 11. ^{13}C NMR spectra of the compound 2c

Data File: C:\ LabSolutions\ Data\Analiz\derya\ FA-3_3.lcd

Elmt	Val.	Min	Max	Elmt	Val.	Min	Max	Elmt	Val.	Min	Max	Use Adduct
H	1	0	50	O	2	0	6	Cl	1	0	0	H
C	4	0	50	F	1	0	0	Br	1	0	0	
N	3	0	4	S	2	0	0	Pd	2	0	0	

Error Margin (ppm):	5	DBE Range:	10.0 - 20.0	Electron Ions:	both
HC Ratio:	unlimited	Apply N Rule:	yes	Use MSn Info:	no
Max Isotopes:	3	Isotope RI (%):	1.00	Isotope Res:	10000
MSn Iso RI (%):	10.00	MSn Logic Mode:	AND	Max Results:	500

Event#: 1 MS(E+) Ret. Time : 9.067 -> 9.067 Scan# : 1361 -> 1361

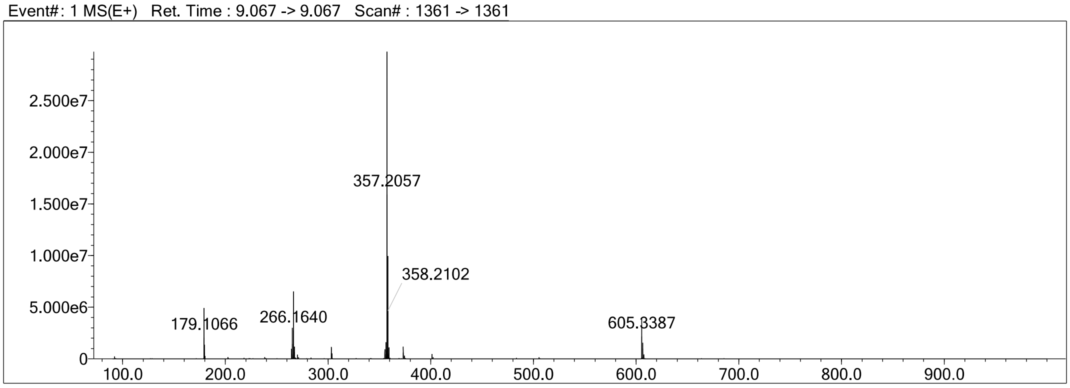

Measured region for 357.2057 m/ z

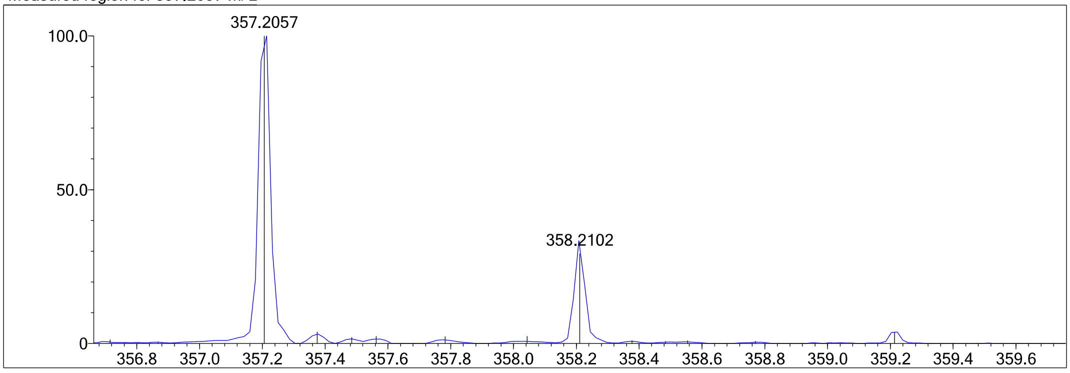

C23 H24 N4 [M+H]+ : Predicted region for 357.2074 m/ z

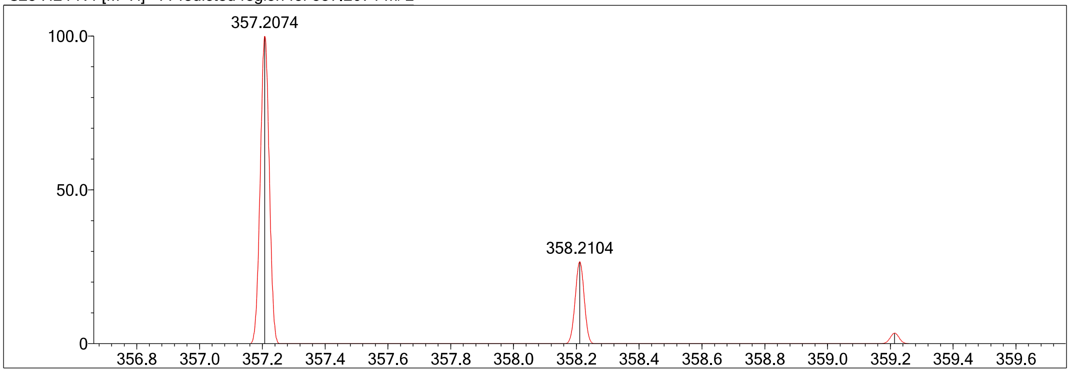

Rank	Score	Formula (M)	Ion	Meas. m/ z	Pred. m/ z	Df. (mDa)	Df. (ppm)	Iso	DBE
1	83.47	C23 H24 N4	[M+H]+	357.2057	357.2074	-1.7	-4.76	92.13	14.0

Spectra 12. HRMS spectra of the compound 2c

DOPNALAB

	Item	Value
	Acquired Date&Time	5.10.2016 09:56:50
	Acquired by	System Administrator
	Filename	C:\Users\dopnalab\Desktop\derya\fa\fa-2d1.ispd
	Spectrum name	fa-2d1
	Sample name	fa_2d
	Sample ID	
	Option	
	Comment	
	No. of Scans	10
	Resolution	4 [cm-1]
	Apodization	Happ-Genzel

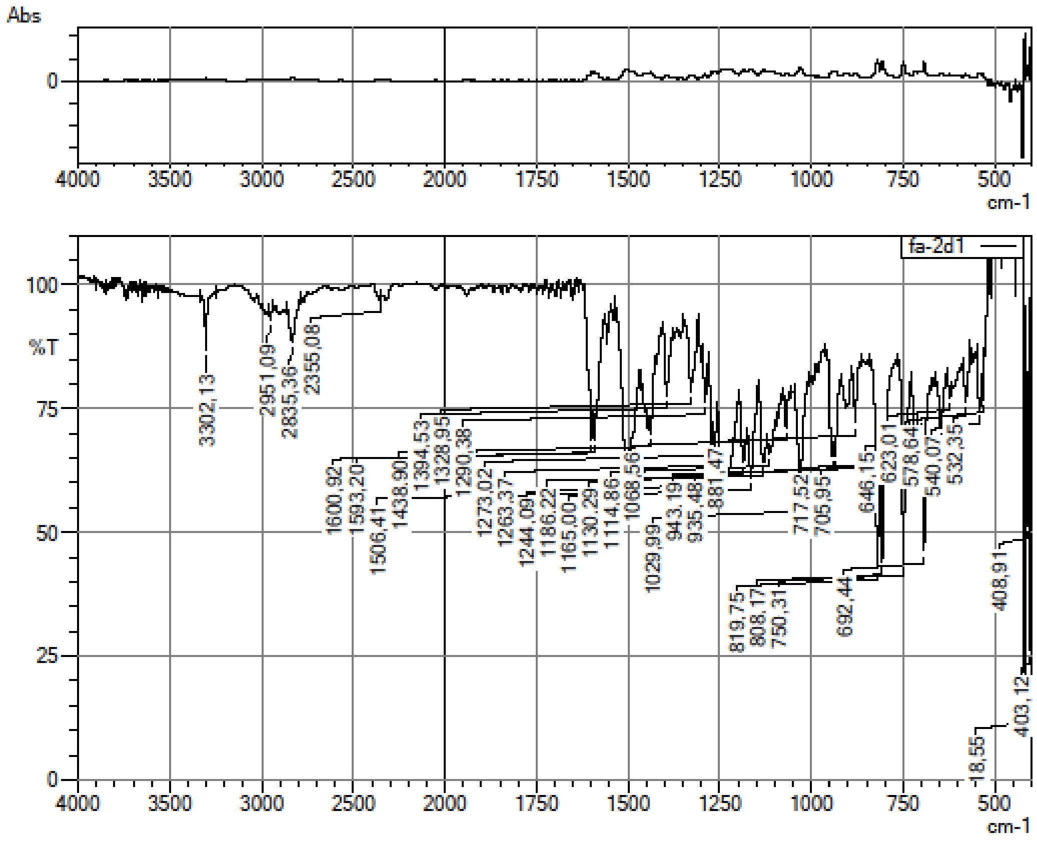

Spectra 13. IR spectra of the compound 2d

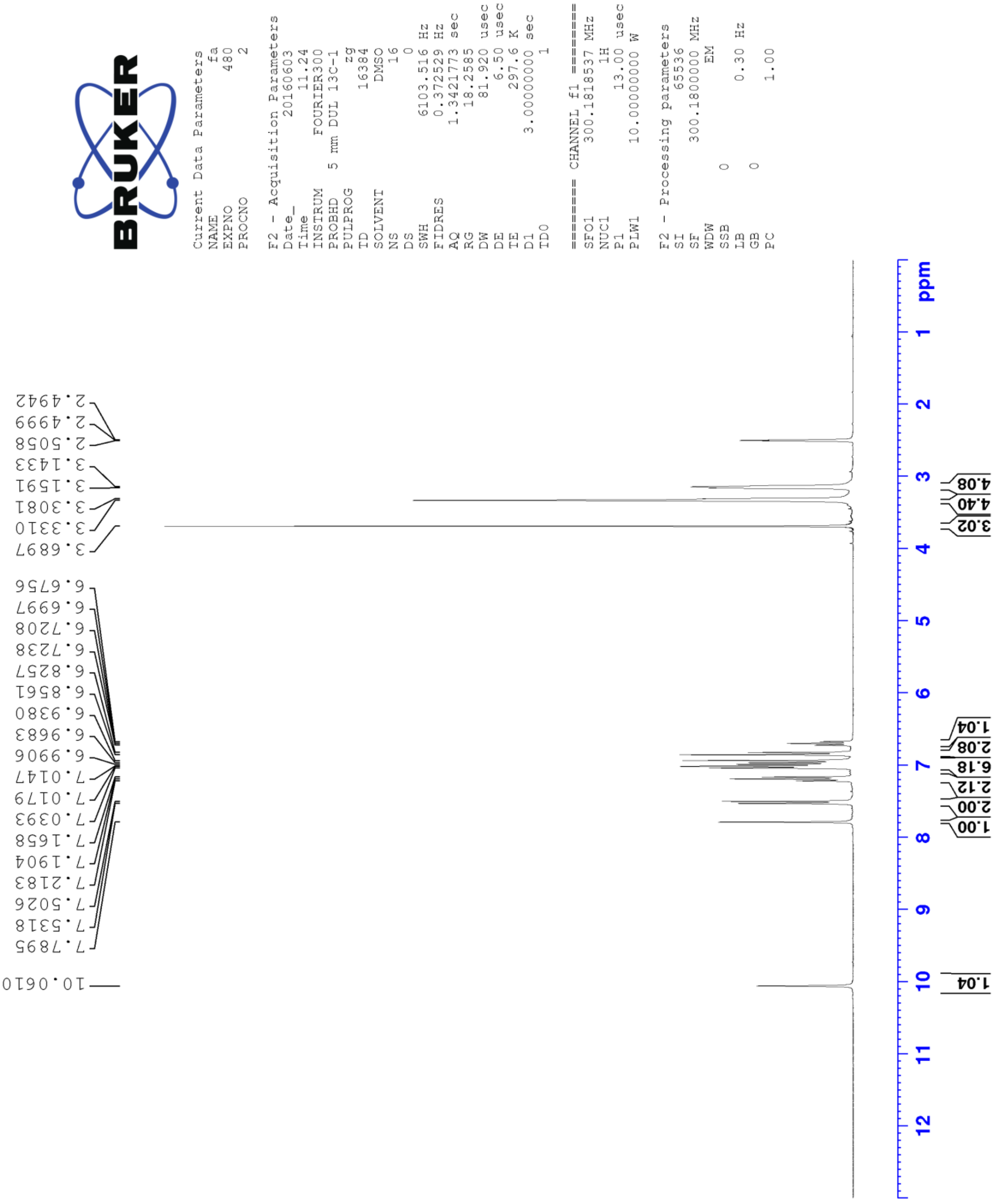

Spectra 14. ^{1}H NMR spectra of the compound 2d

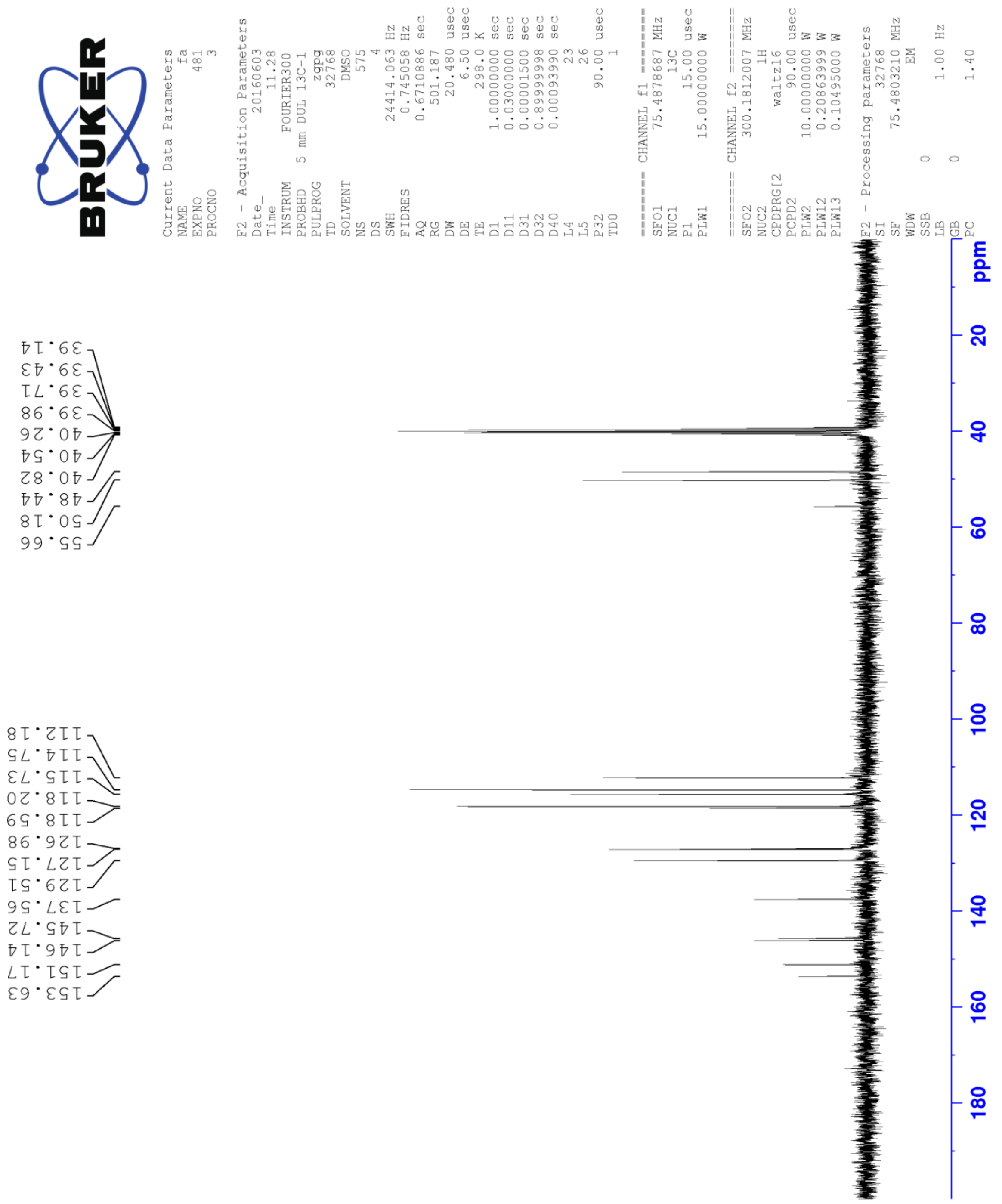

Spectra 15. ^{13}C NMR spectra of the compound 2d

Data File: C:\ LabSolutions\ Data\Analiz\derya\ FA-4_4.lcd

Elmt	Val.	Min	Max	Elmt	Val.	Min	Max	Elmt	Val.	Min	Max	Use Adduct
H	1	0	50	O	2	0	6	Cl	1	0	0	H
C	4	0	50	F	1	0	0	Br	1	0	0	
N	3	0	4	S	2	0	0	Pd	2	0	0	

Error Margin (ppm):	5	DBE Range:	10.0 - 20.0	Electron Ions:	both
HC Ratio:	unlimited	Apply N Rule:	yes	Use MSn Info:	no
Max Isotopes:	3	Isotope RI (%):	1.00	Isotope Res:	10000
MSn Iso RI (%):	10.00	MSn Logic Mode:	AND	Max Results:	500

Event#: 1 MS(E+) Ret. Time : 8.200 -> 8.200 Scan# : 1231 -> 1231

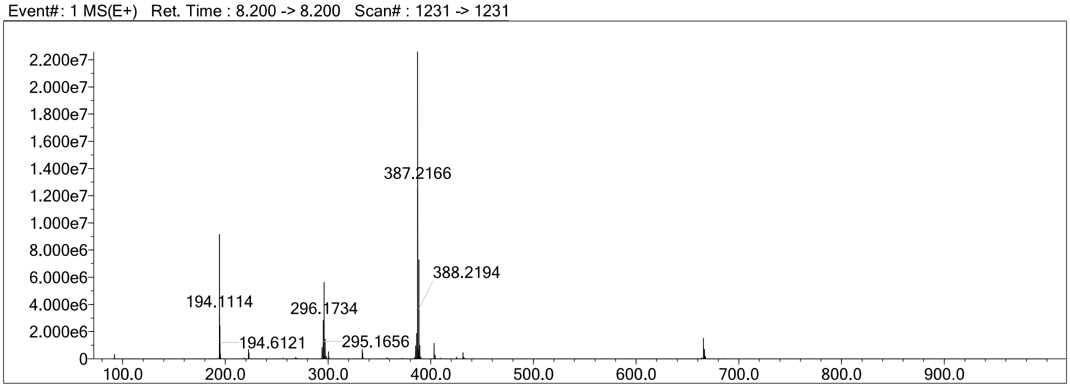

Measured region for 387.2166 m/z

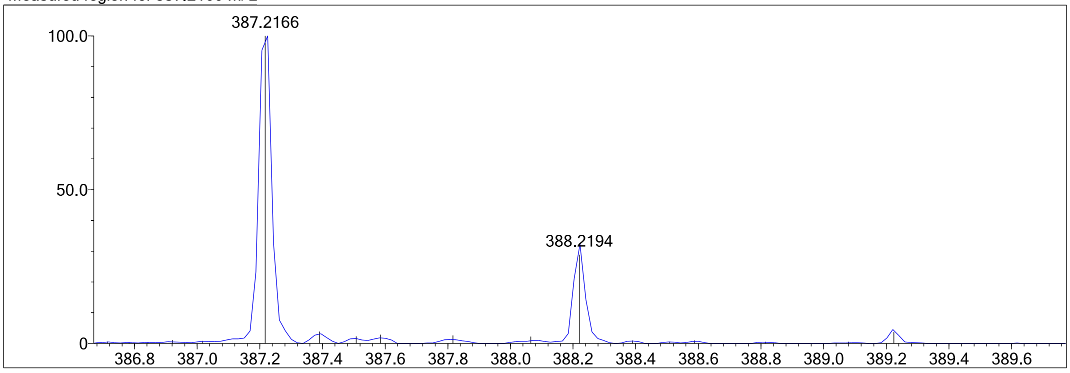

C24 H26 N4 O [M+H]+ : Predicted region for 387.2179 m/z

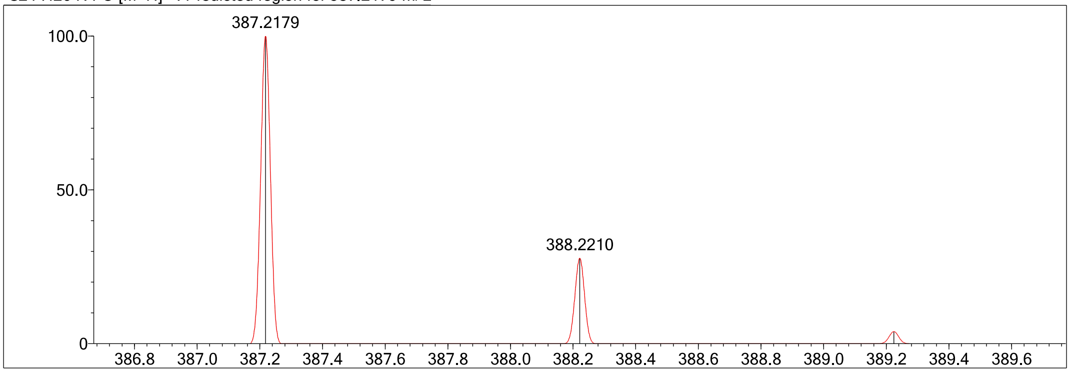

Rank	Score	Formula (M)	Ion	Meas. m/z	Pred. m/z	Df. (mDa)	Df. (ppm)	Iso	DBE
1	92.48	C24 H26 N4 O	[M+H]+	387.2166	387.2179	-1.3	-3.36	98.28	14.0

Spectra 16. HRMS spectra of the compound 2d

DOPNALAB

Item	Value
Acquired Date&Time	5.10.2016 10:00:28
Acquired by	System Administrator
Filename	C:\Users\dopnalab\Desktop\derya\fa\fa-2e1.ispd
Spectrum name	fa-2e1
Sample name	fa_2e
Sample ID	
Option	
Comment	
No. of Scans	10
Resolution	4 [cm-1]
Apodization	Happ-Genzel

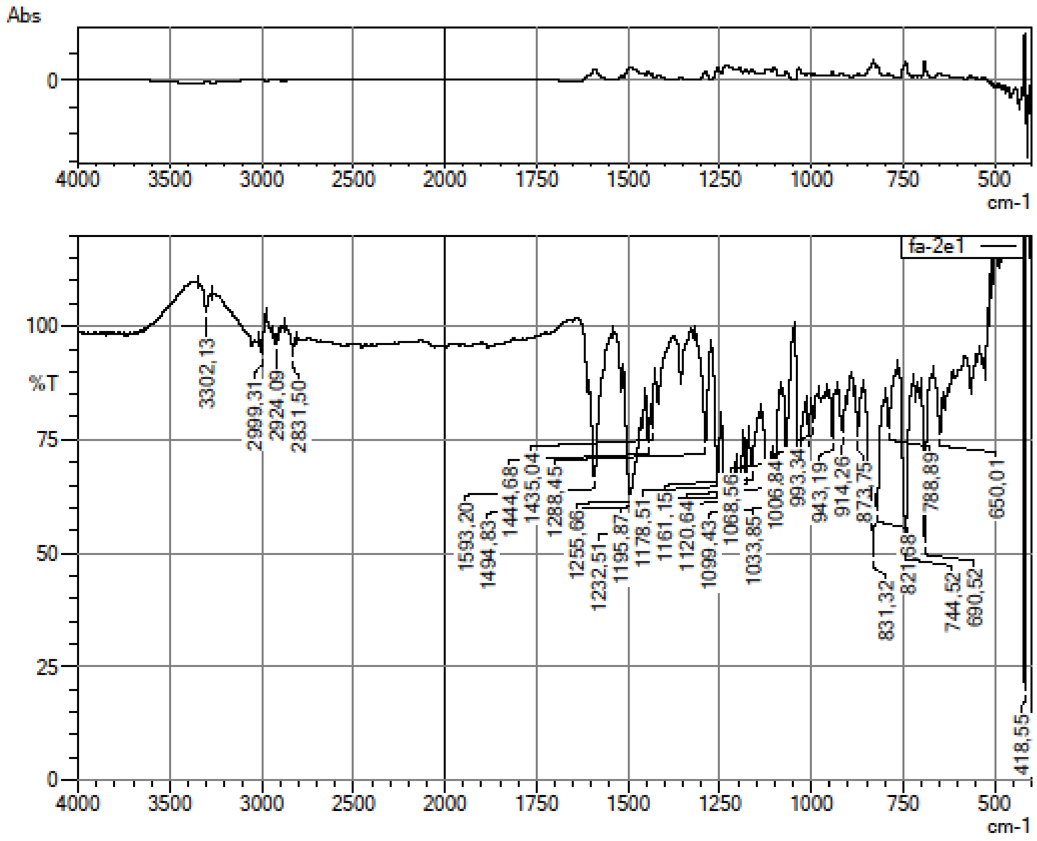

Spectra 17. IR spectra of the compound 2e

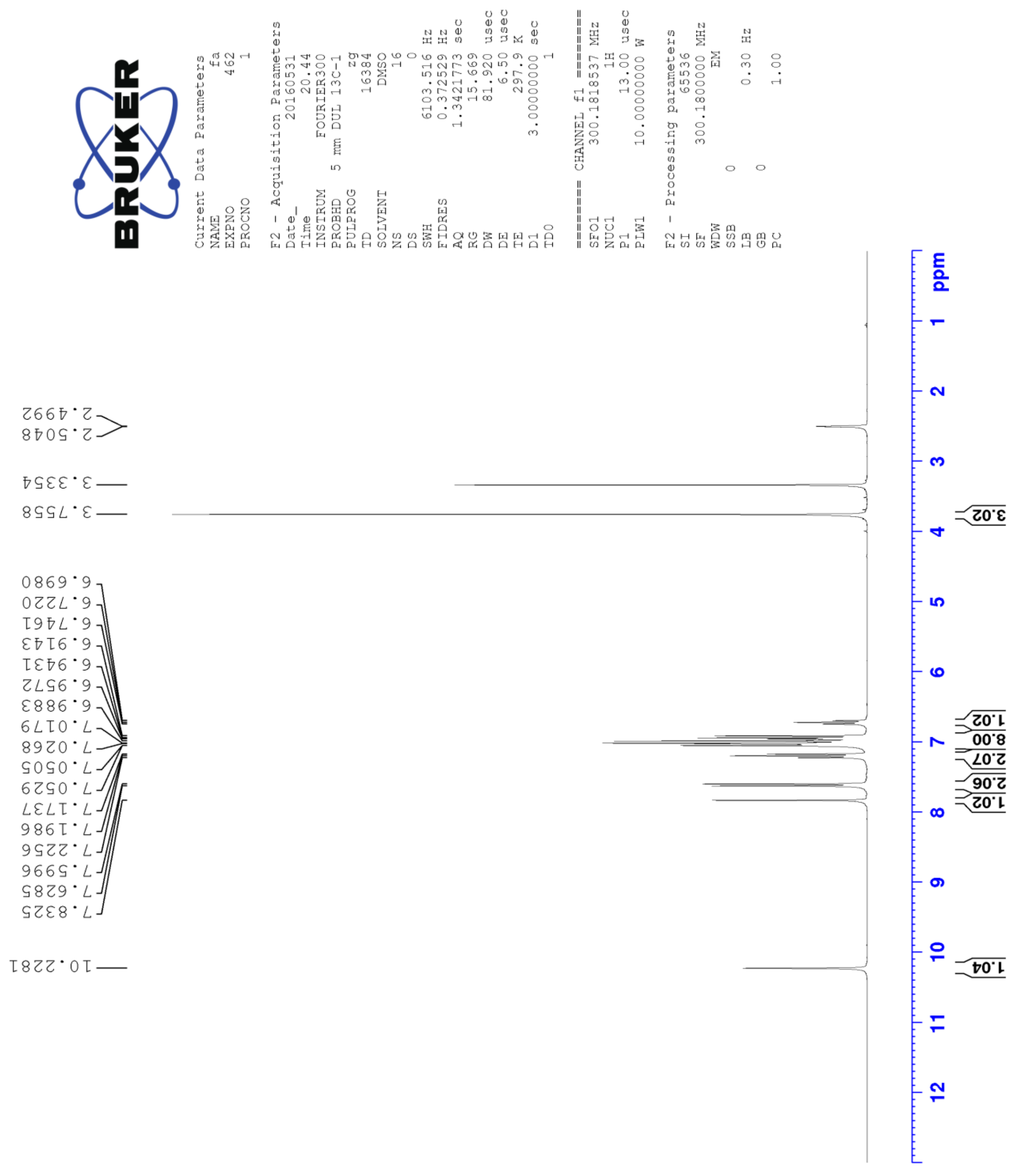

Spectra 18. ^{1}H NMR spectra of the compound 2e

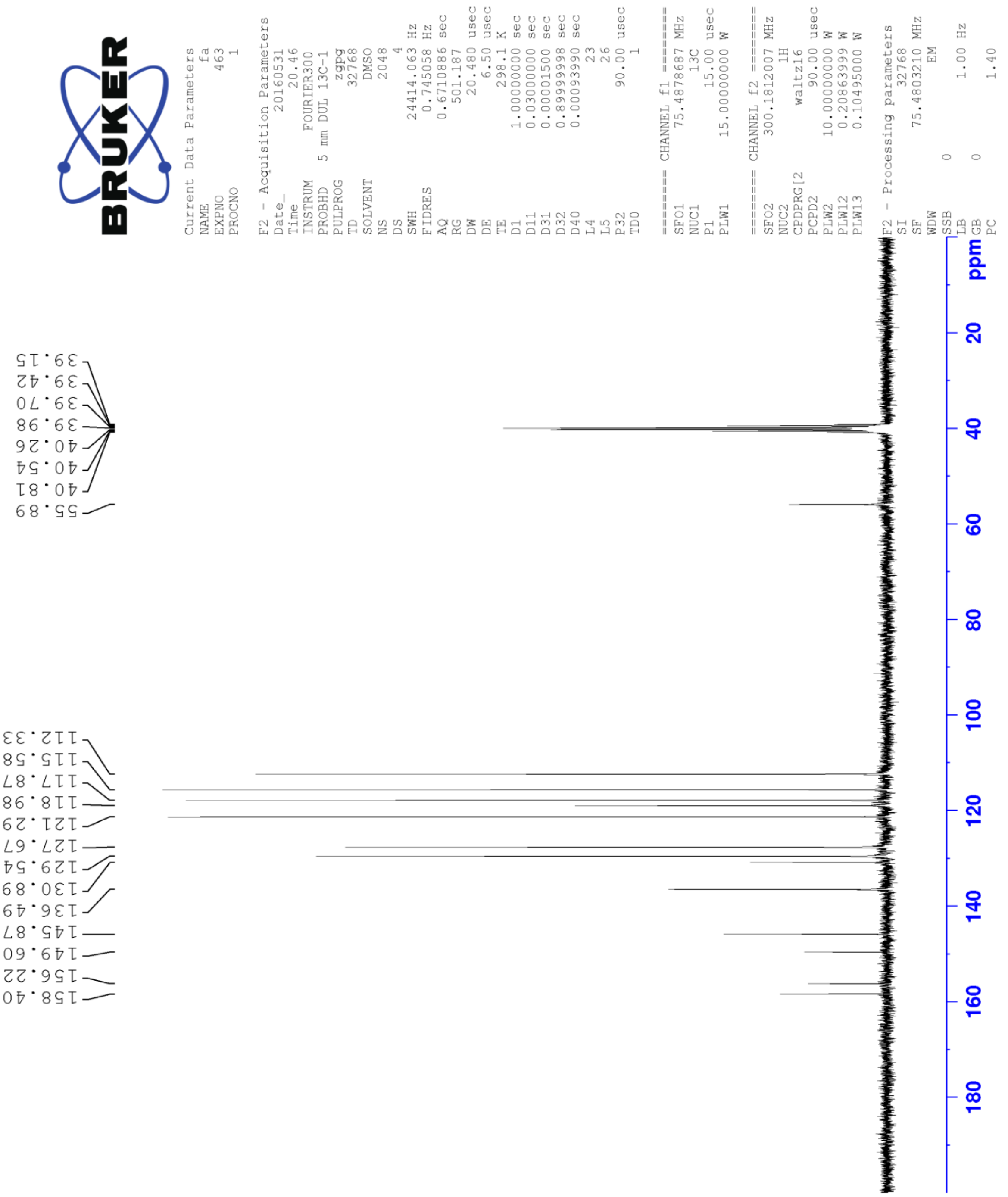

Spectra 19. ^{13}C NMR spectra of the compound 2e

Data File: C:\ LabSolutions\ Data\Analiz\derya\ FA-5_5.lcd

Elmt	Val.	Min	Max	Elmt	Val.	Min	Max	Elmt	Val.	Min	Max	Use Adduct
H	1	0	50	O	2	0	6	Cl	1	0	0	H
C	4	0	50	F	1	0	0	Br	1	0	0	
N	3	0	4	S	2	0	0	Pd	2	0	0	

Error Margin (ppm):	5	DBE Range:	10.0 - 20.0	Electron Ions:	both
HC Ratio:	unlimited	Apply N Rule:	yes	Use MSn Info:	no
Max Isotopes:	3	Isotope RI (%):	1.00	Isotope Res:	10000
MSn Iso RI (%):	10.00	MSn Logic Mode:	AND	Max Results:	500

Event#: 1 MS(E+) Ret. Time : 8.867 -> 8.867 Scan# : 1331 -> 1331

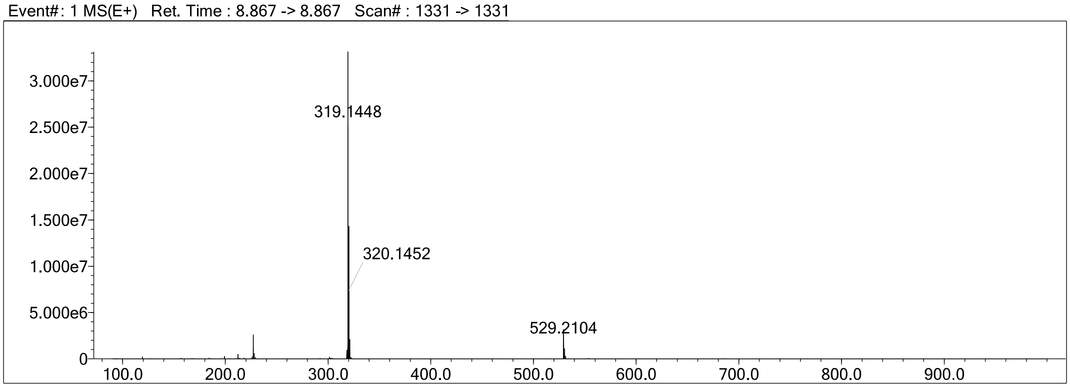

Measured region for 319.1448 m/z

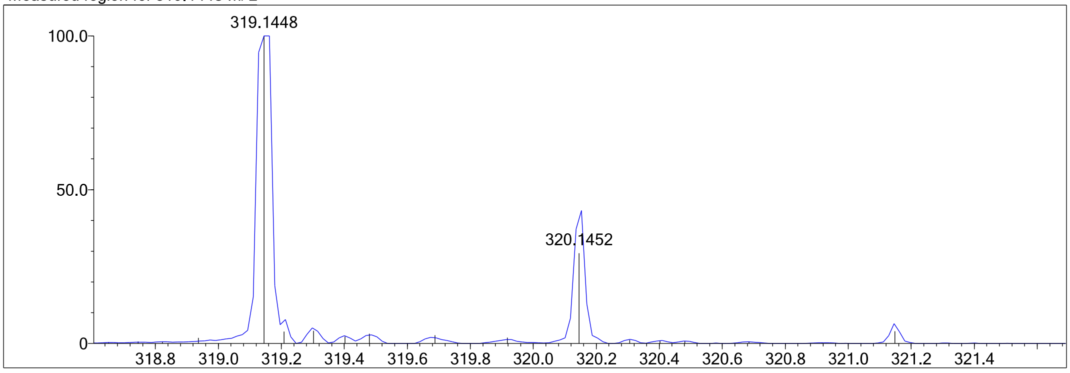

C20 H18 N2 O2 [M+H]+ : Predicted region for 319.1441 m/z

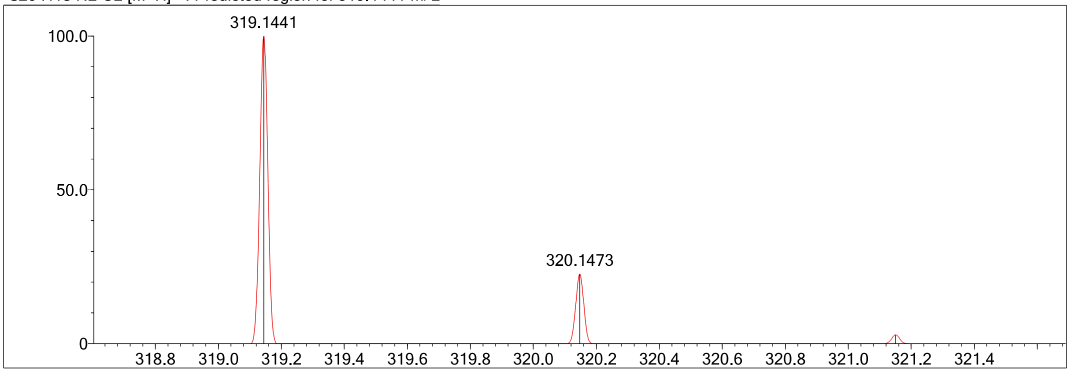

Rank	Score	Formula (M)	Ion	Meas. m/z	Pred. m/z	Df. (mDa)	Df. (ppm)	Iso	DBE
1	59.52	C20 H18 N2 O2	[M+H]+	319.1448	319.1441	0.7	2.19	61.35	13.0

Spectra 20. HRMS spectra of the compound 2e

DOPNALAB

	Item	Value
	Acquired Date&Time	5.10.2016 10:03:22
	Acquired by	System Administrator
	Filename	C:\Users\dopnalab\Desktop\derya\fa\fa-2f1.ispd
	Spectrum name	fa-2f1
	Sample name	fa_2f
	Sample ID	
	Option	
	Comment	
	No. of Scans	10
	Resolution	4 [cm-1]
	Apodization	Happ-Genzel

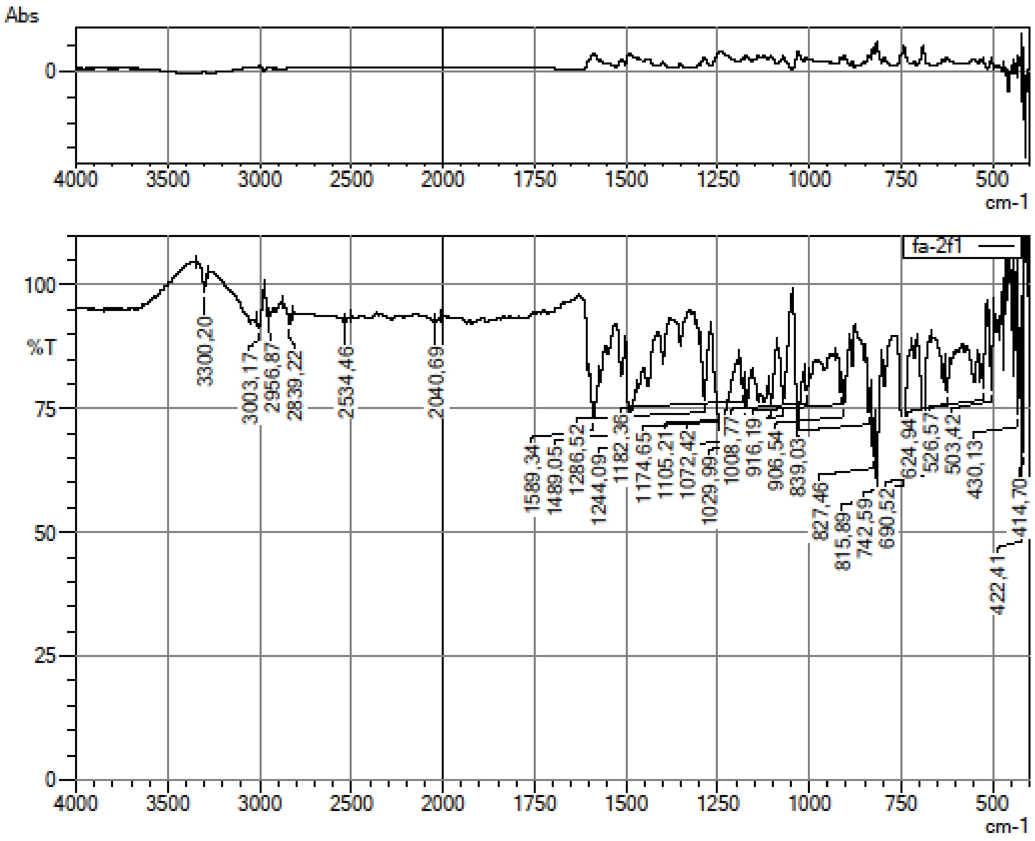

Spectra 21. IR spectra of the compound 2f

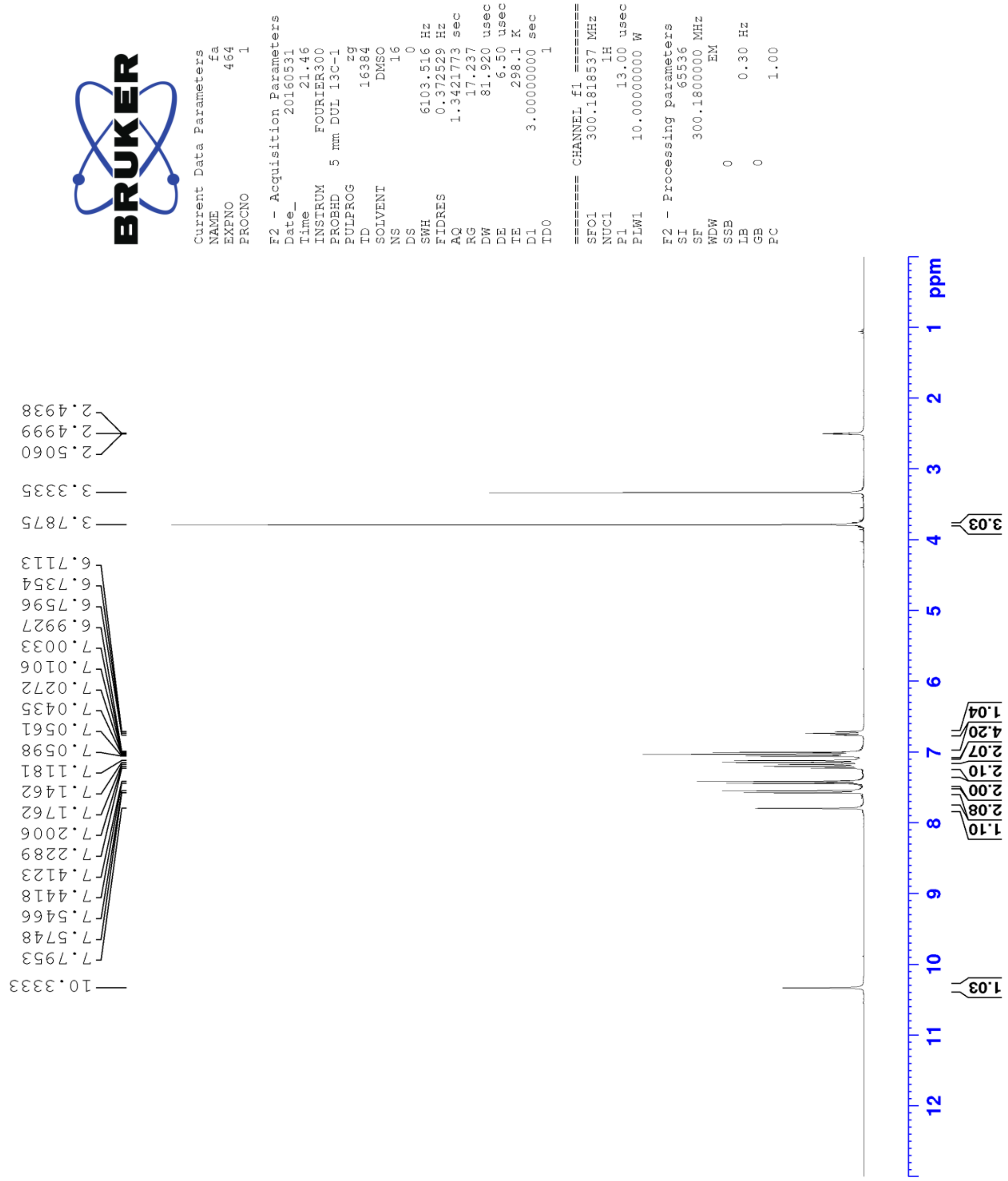

Spectra 22. ^{1}H NMR spectra of the compound 2f

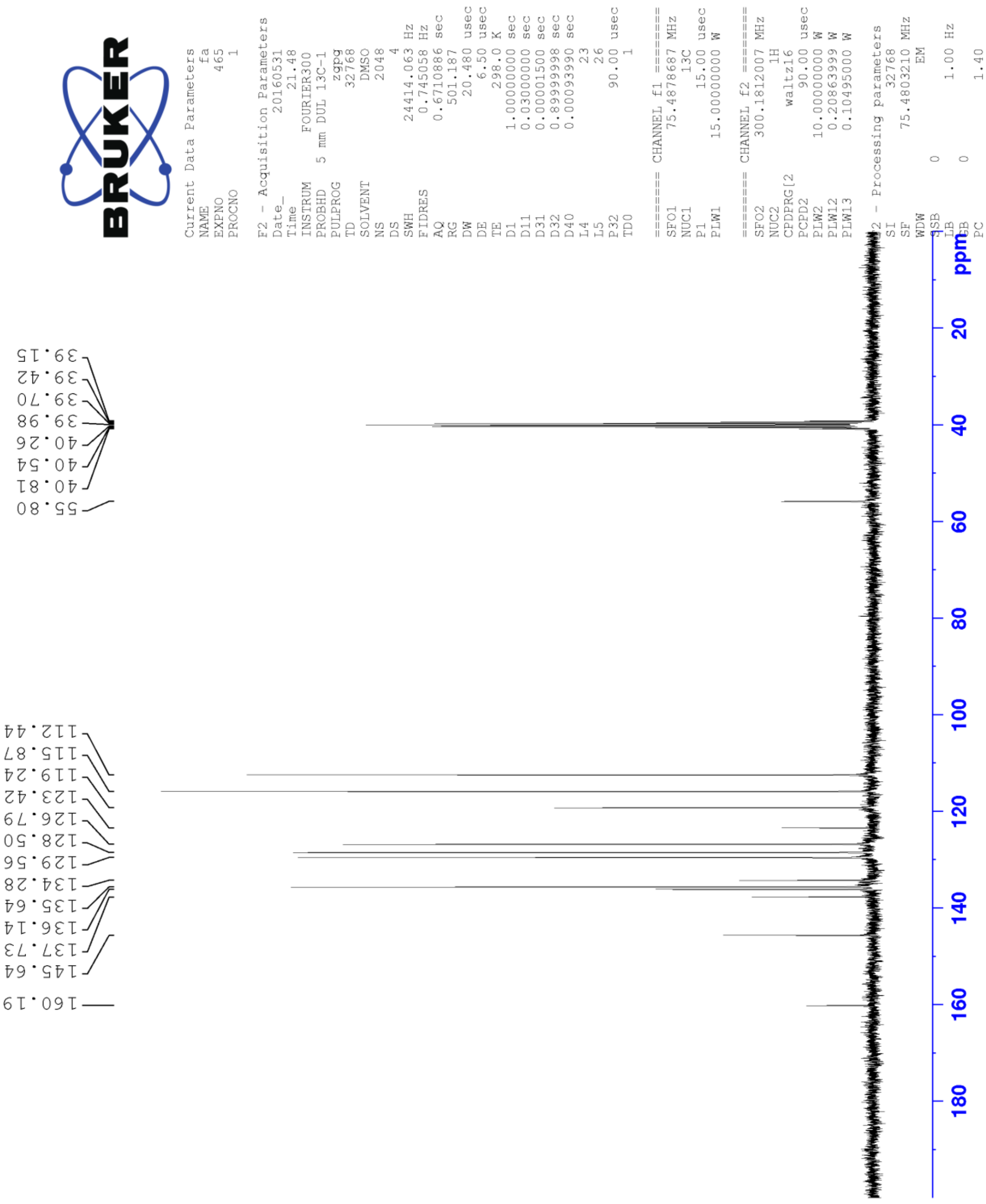

Spectra 23. ^{13}C NMR spectra of the compound 2f

Data File: C:\ LabSolutions\ Data\Analiz\derya\ FA-6_6.lcd

Elmt	Val.	Min	Max	Elmt	Val.	Min	Max	Elmt	Val.	Min	Max	Use Adduct
H	1	0	50	O	2	0	6	Cl	1	0	0	H
C	4	0	50	F	1	0	0	Br	1	0	0	
N	3	0	4	S	2	0	1	Pd	2	0	0	

Error Margin (ppm):	5	DBE Range:	10.0 - 20.0	Electron Ions:	both
HC Ratio:	unlimited	Apply N Rule:	yes	Use MSn Info:	no
Max Isotopes:	3	Isotope RI (%):	1.00	Isotope Res:	10000
MSn Iso RI (%):	10.00	MSn Logic Mode:	AND	Max Results:	500

Event#: 1 MS(E+) Ret. Time : 9.440 -> 9.440 Scan# : 1417 -> 1417

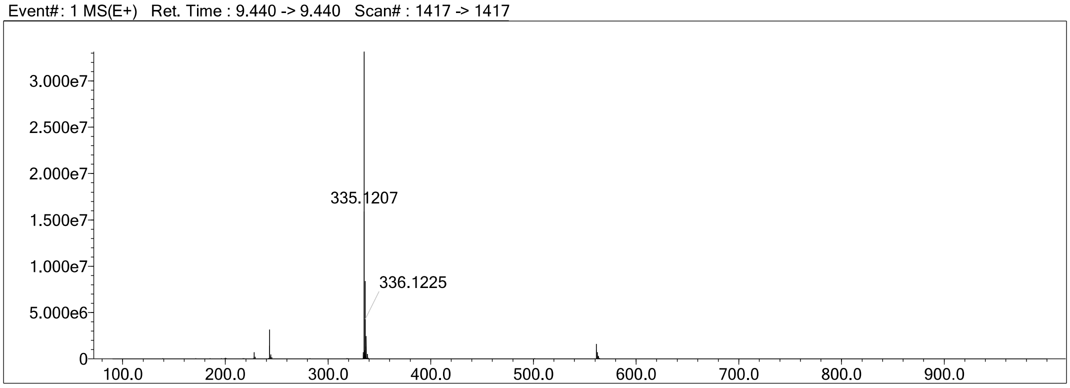

Measured region for 335.1207 m/z

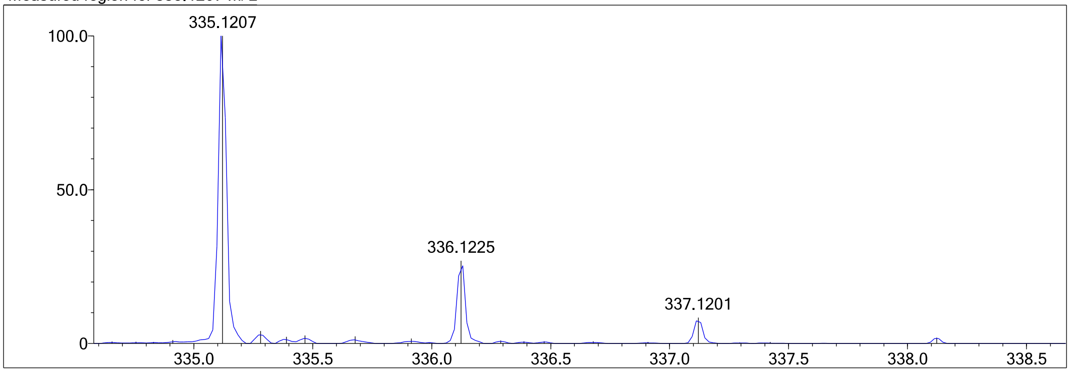

C20 H18 N2 O S [M+H]+ : Predicted region for 335.1213 m/z

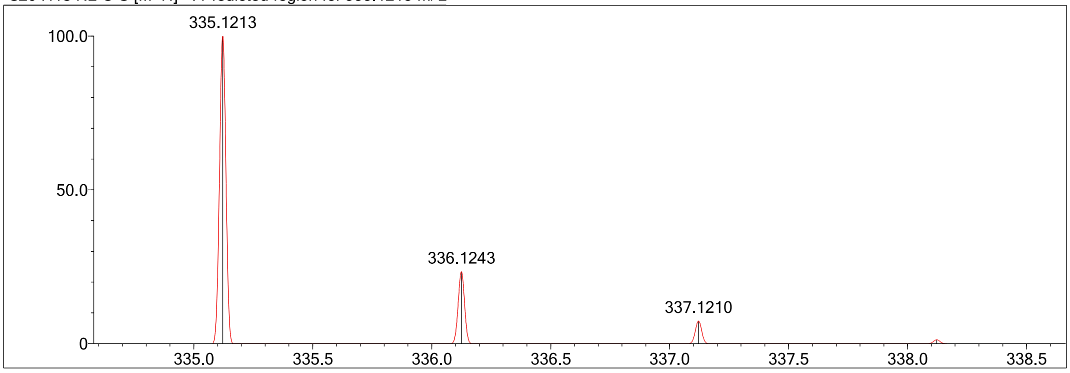

Rank	Score	Formula (M)	Ion	Meas. m/z	Pred. m/z	Df. (mDa)	Df. (ppm)	Iso	DBE
1	77.61	C20 H18 N2 O S	[M+H]+	335.1207	335.1213	-0.6	-1.79	79.17	13.0

Spectra 24. HRMS spectra of the compound 2f

DOPNALAB

Item	Value
Acquired Date&Time	5.10.2016 10:06:35
Acquired by	System Administrator
Filename	C:\Users\dopnalab\Desktop\derya\fa\fa-2g1.ispd
Spectrum name	fa-2g1
Sample name	fa_2g
Sample ID	
Option	
Comment	
No. of Scans	10
Resolution	4 [cm-1]
Apodization	Happ-Genzel

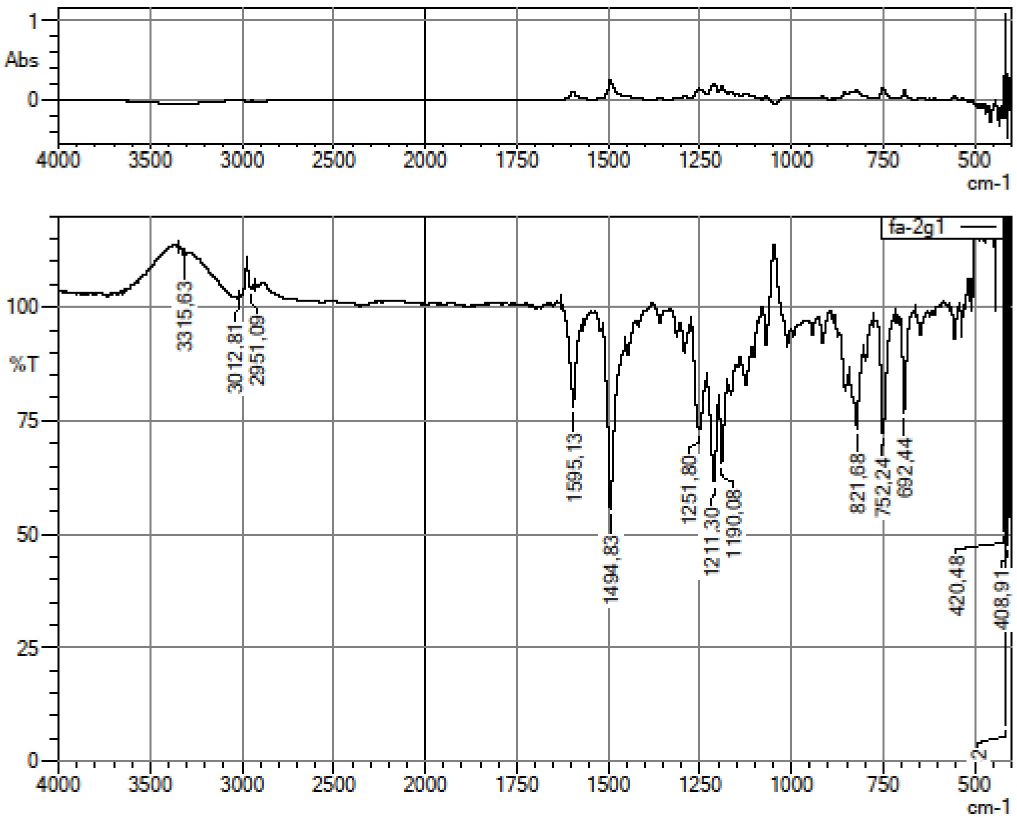

Spectra 25. IR spectra of the compound 2g

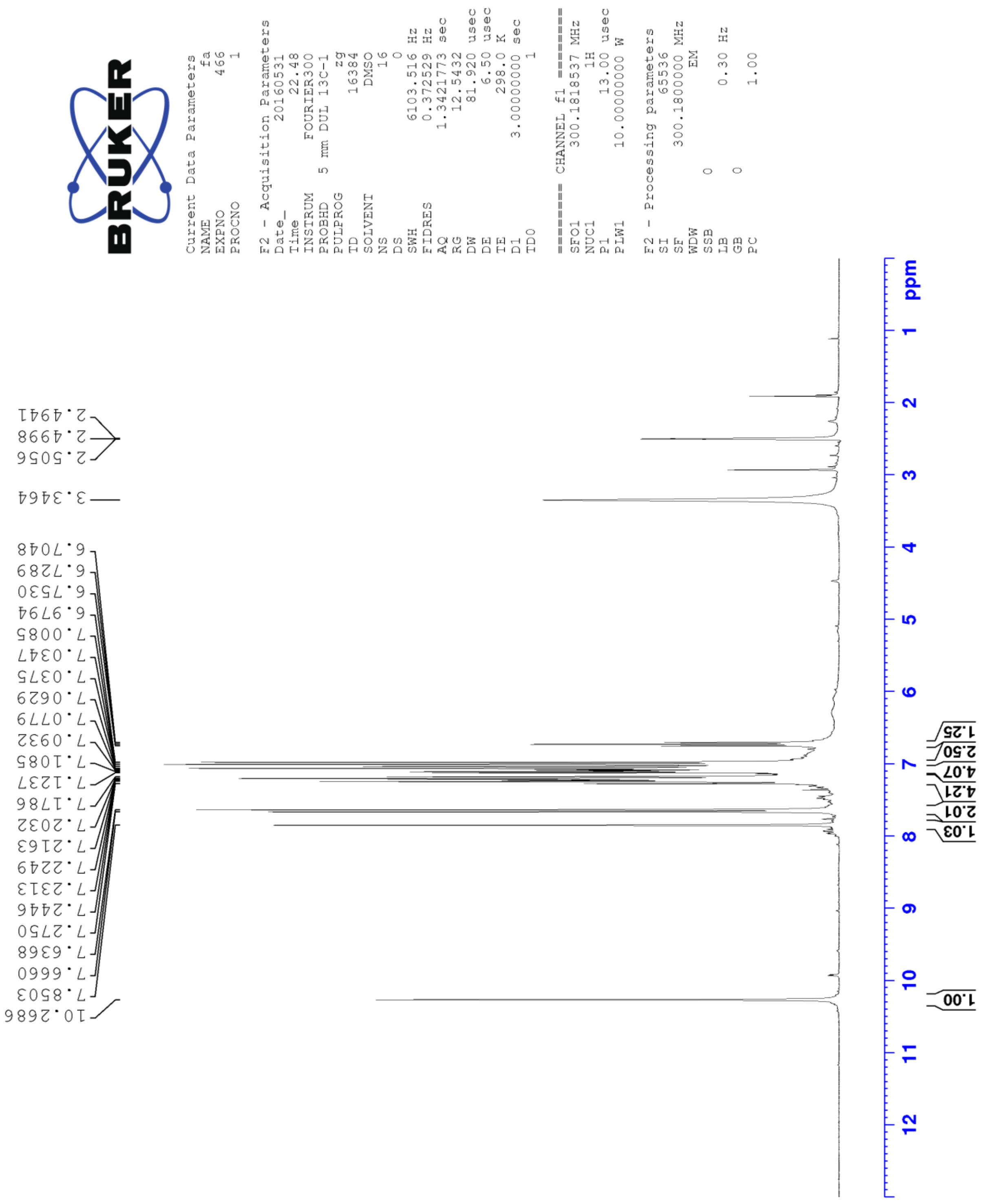

Spectra 26. ^{1}H NMR spectra of the compound 2g

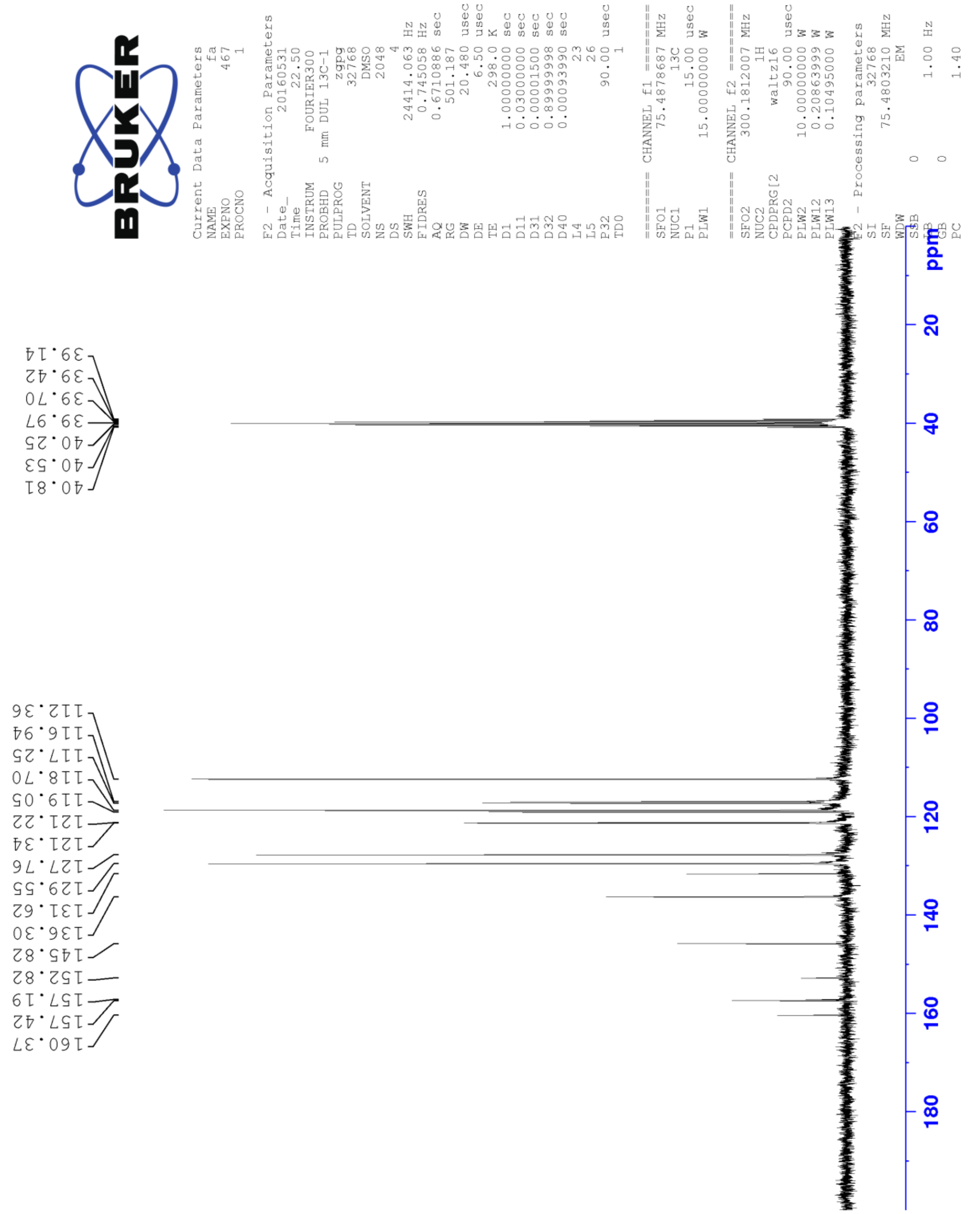

Spectra 27. ^{13}C NMR spectra of the compound 2g

Data File: C:\ LabSolutions\ Data\Analiz\derya\ FA-7_7.lcd

Elmt	Val.	Min	Max	Elmt	Val.	Min	Max	Elmt	Val.	Min	Max	Use Adduct
H	1	0	50	O	2	0	6	Cl	1	0	0	H
C	4	0	50	F	1	1	1	Br	1	0	0	
N	3	0	4	S	2	0	1	Pd	2	0	0	

Error Margin (ppm):	5	DBE Range:	10.0 - 20.0	Electron Ions:	both
HC Ratio:	unlimited	Apply N Rule:	yes	Use MSn Info:	no
Max Isotopes:	3	Isotope RI (%):	1.00	Isotope Res:	10000
MSn Iso RI (%):	10.00	MSn Logic Mode:	AND	Max Results:	500

Event#: 1 MS(E+) Ret. Time : 9.000 -> 9.000 Scan# : 1351 -> 1351

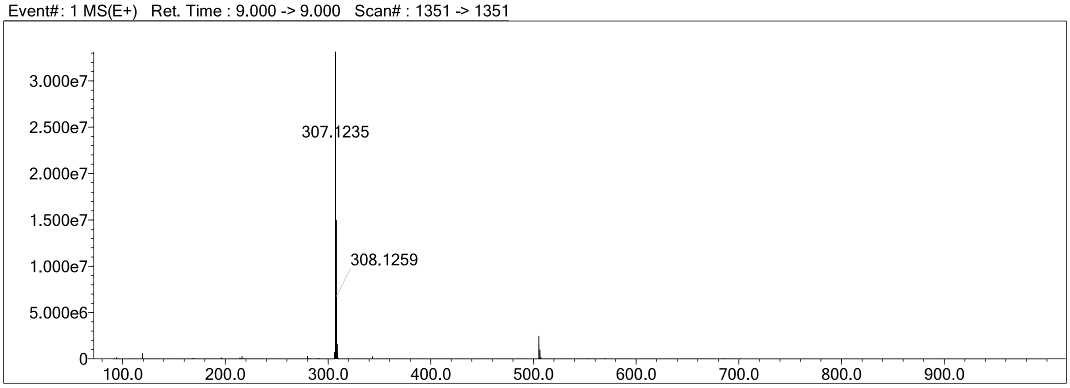

Measured region for 307.1235 m/ z

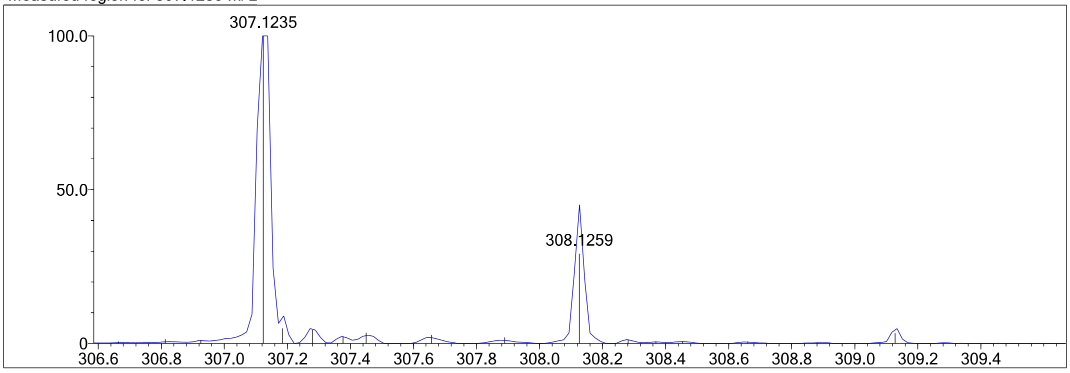

C19 H15 N2 O F [M+H]+ : Predicted region for 307.1241 m/ z

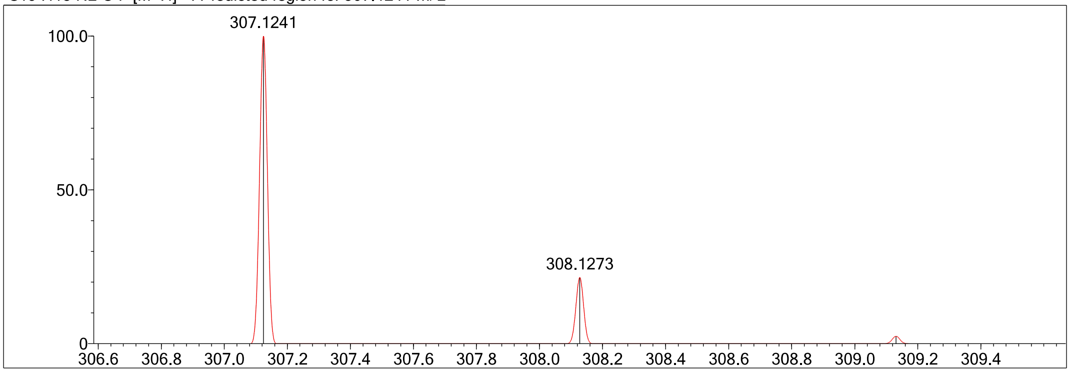

Rank	Score	Formula (M)	Ion	Meas. m/ z	Pred. m/ z	Df. (mDa)	Df. (ppm)	Iso	DBE
1	59.86	C19 H15 N2 O F	[M+H]+	307.1235	307.1241	-0.6	-1.95	61.32	13.0

Spectra 28. HRMS spectra of the compound 2g

DOPNALAB

	Item	Value
	Acquired Date&Time	5.10.2016 10:09:19
	Acquired by	System Administrator
	Filename	C:\Users\dopnalab\Desktop\derya\fa\fa-2h1.ispd
	Spectrum name	fa-2h1
	Sample name	fa_2h
	Sample ID	
	Option	
	Comment	
	No. of Scans	10
	Resolution	4 [cm-1]
	Apodization	Happ-Genzel

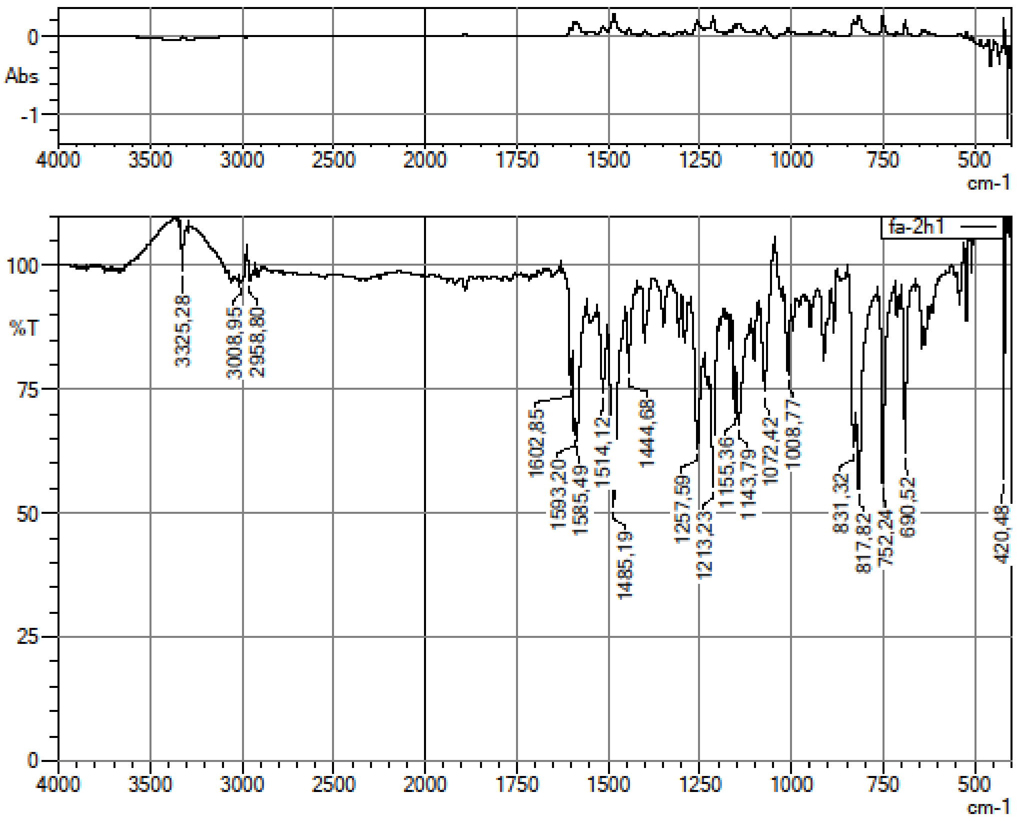

Spectra 29. IR spectra of the compound 2h

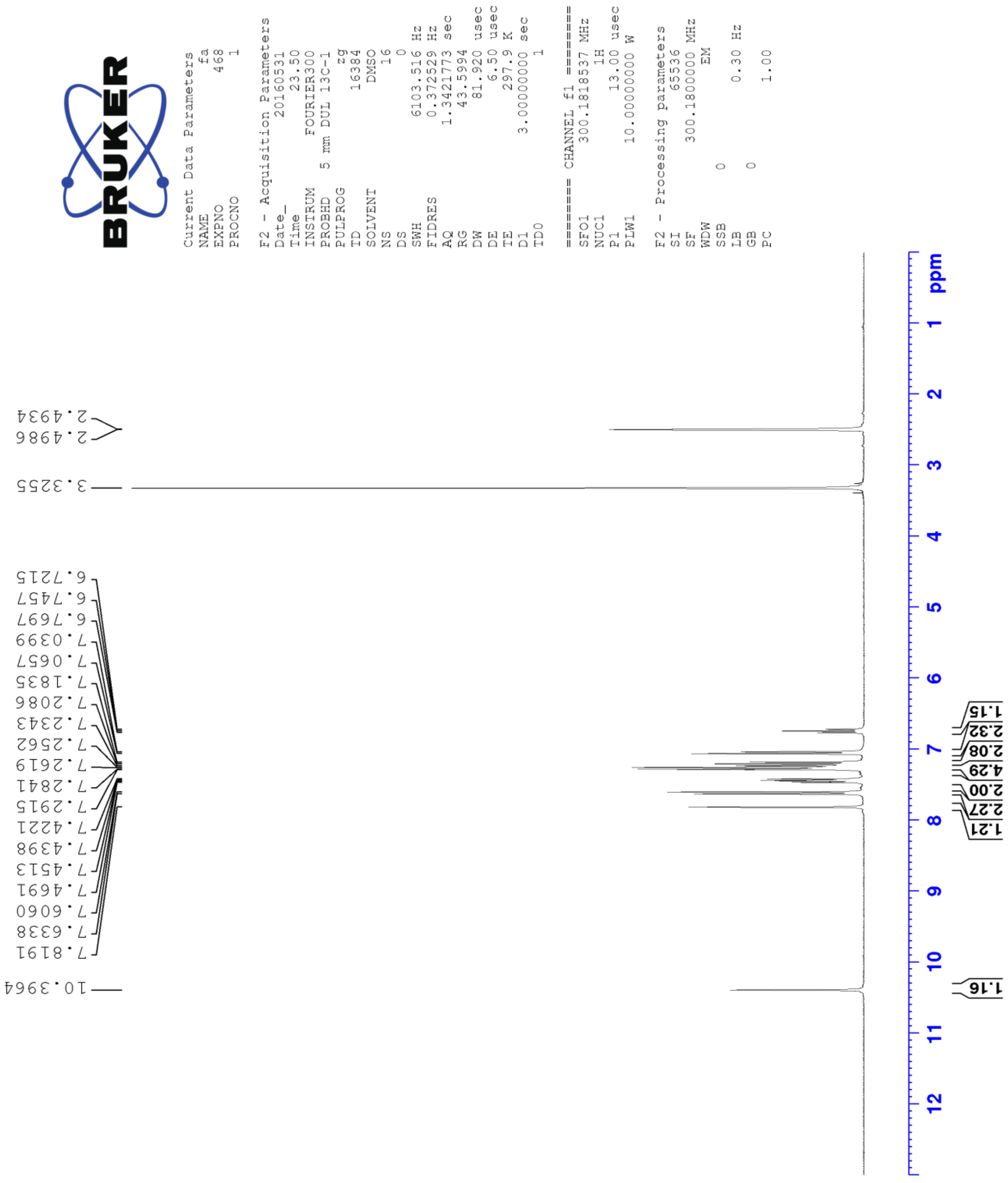

Spectra 30. ^{1}H NMR spectra of the compound 2h

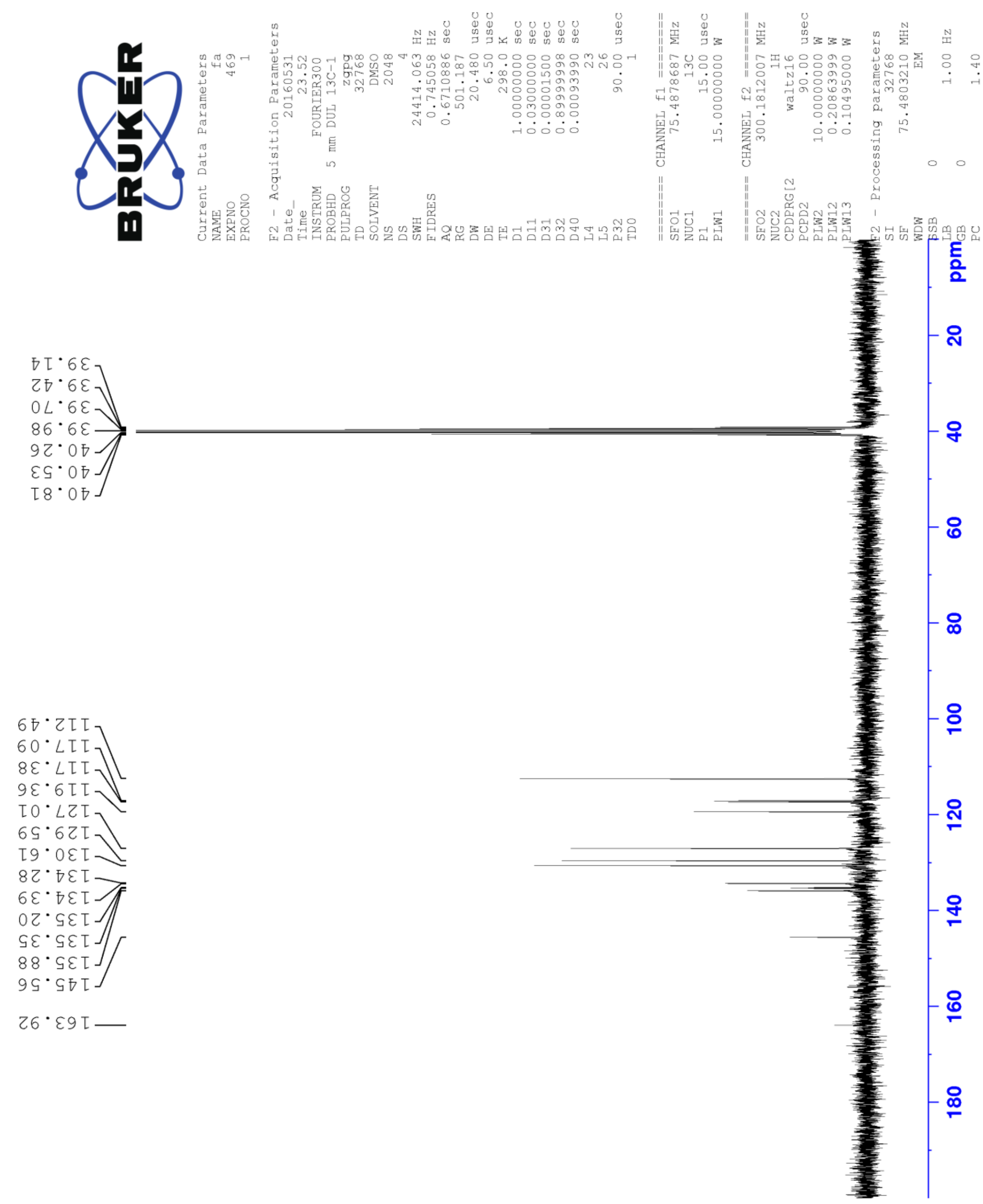

Spectra 31. ^{13}C NMR spectra of the compound 2h

Data File: C:\ LabSolutions\ Data\Analiz\derya\ FA-8_8.lcd

Elmt	Val.	Min	Max	Elmt	Val.	Min	Max	Elmt	Val.	Min	Max	Use Adduct
H	1	0	50	O	2	0	6	Cl	1	0	0	H
C	4	0	50	F	1	1	1	Br	1	0	0	
N	3	0	4	S	2	0	1	Pd	2	0	0	

Error Margin (ppm):	5	DBE Range:	10.0 - 20.0	Electron Ions:	both
HC Ratio:	unlimited	Apply N Rule:	yes	Use MSn Info:	no
Max Isotopes:	3	Isotope RI (%):	1.00	Isotope Res:	10000
MSn Iso RI (%):	10.00	MSn Logic Mode:	AND	Max Results:	500

Event#: 1 MS(E+) Ret. Time : 9.693 -> 9.693 Scan# : 1455 -> 1455

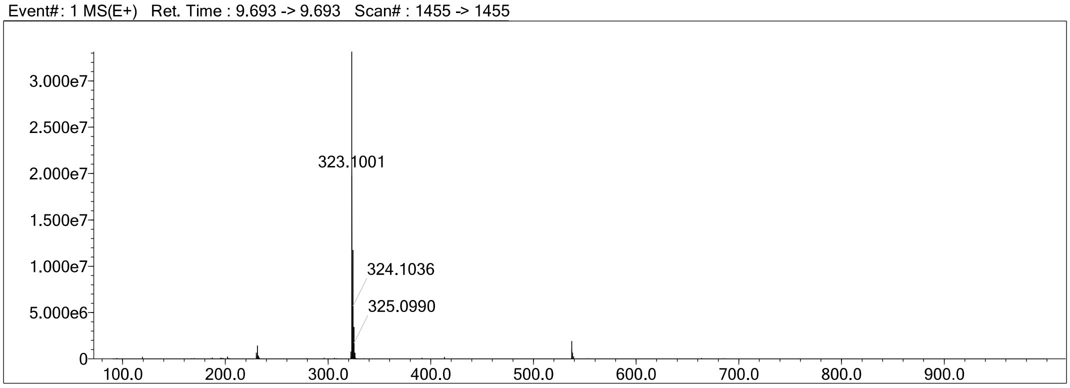

Measured region for 323.1001 m/z

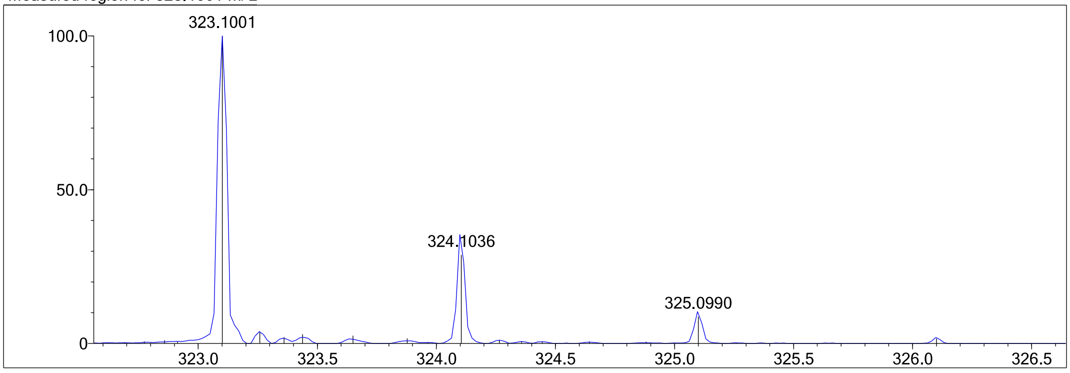

C19 H15 N2 F S [M+H]+ : Predicted region for 323.1013 m/z

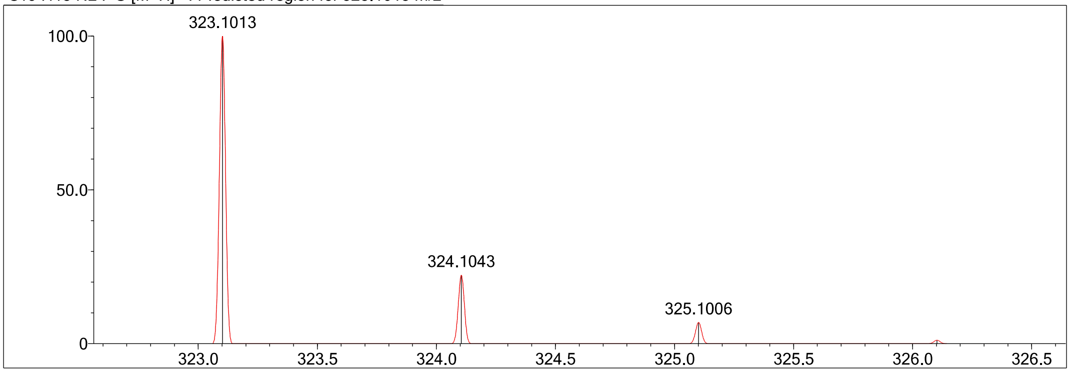

Rank	Score	Formula (M)	Ion	Meas. m/z	Pred. m/z	Df. (mDa)	Df. (ppm)	Iso	DBE
1	73.78	C19 H15 N2 F S	[M+H]+	323.1001	323.1013	-1.2	-3.71	79.14	13.0

Spectra 32. HRMS spectra of the compound 2h

DOPNALAB

	Item	Value
	Acquired Date&Time	5.10.2016 10:17:07
	Acquired by	System Administrator
	Filename	C:\Users\dopnalab\Desktop\derya\fa\fa-2i1.ispd
	Spectrum name	fa-2i1
	Sample name	fa_2i
	Sample ID	
	Option	
	Comment	
	No. of Scans	10
	Resolution	4 [cm-1]
	Apodization	Happ-Genzel

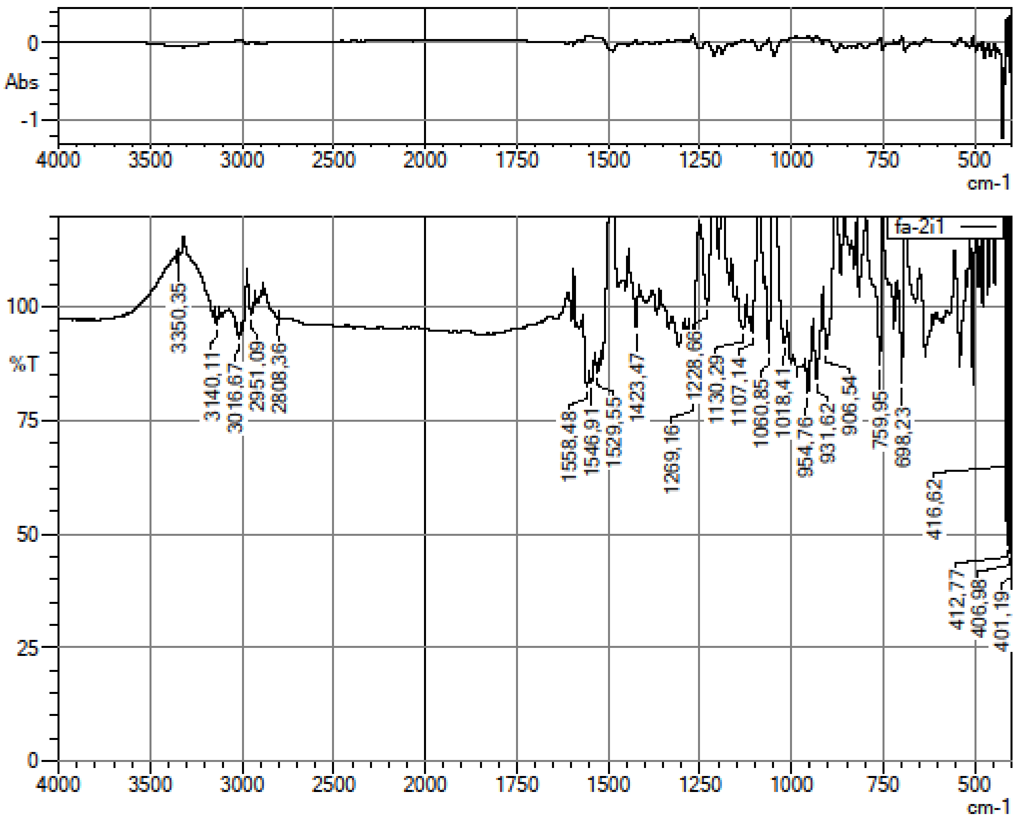

Spectra 33. IR spectra of the compound 2i

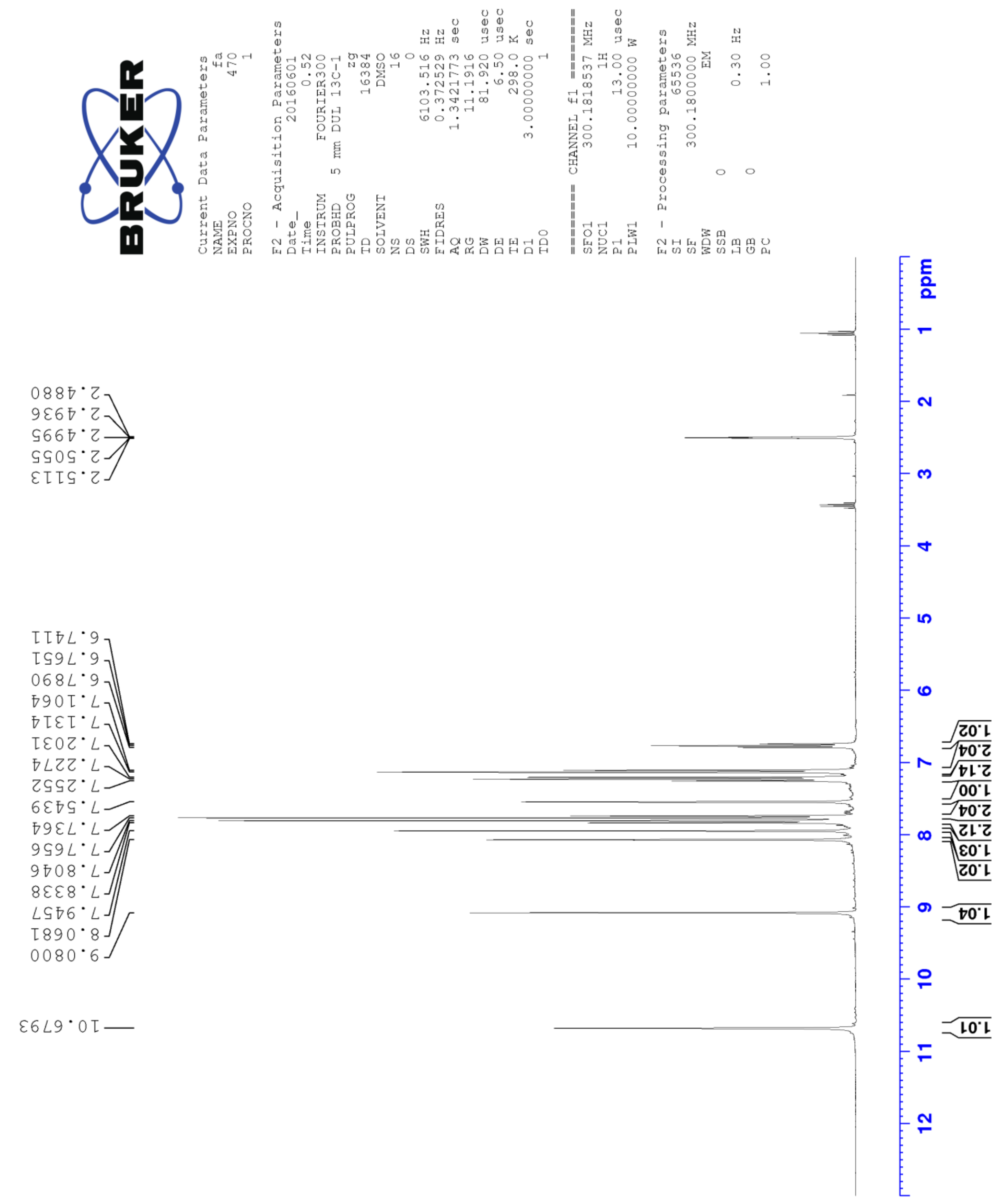

Spectra 34. ^{1}H NMR spectra of the compound 2i

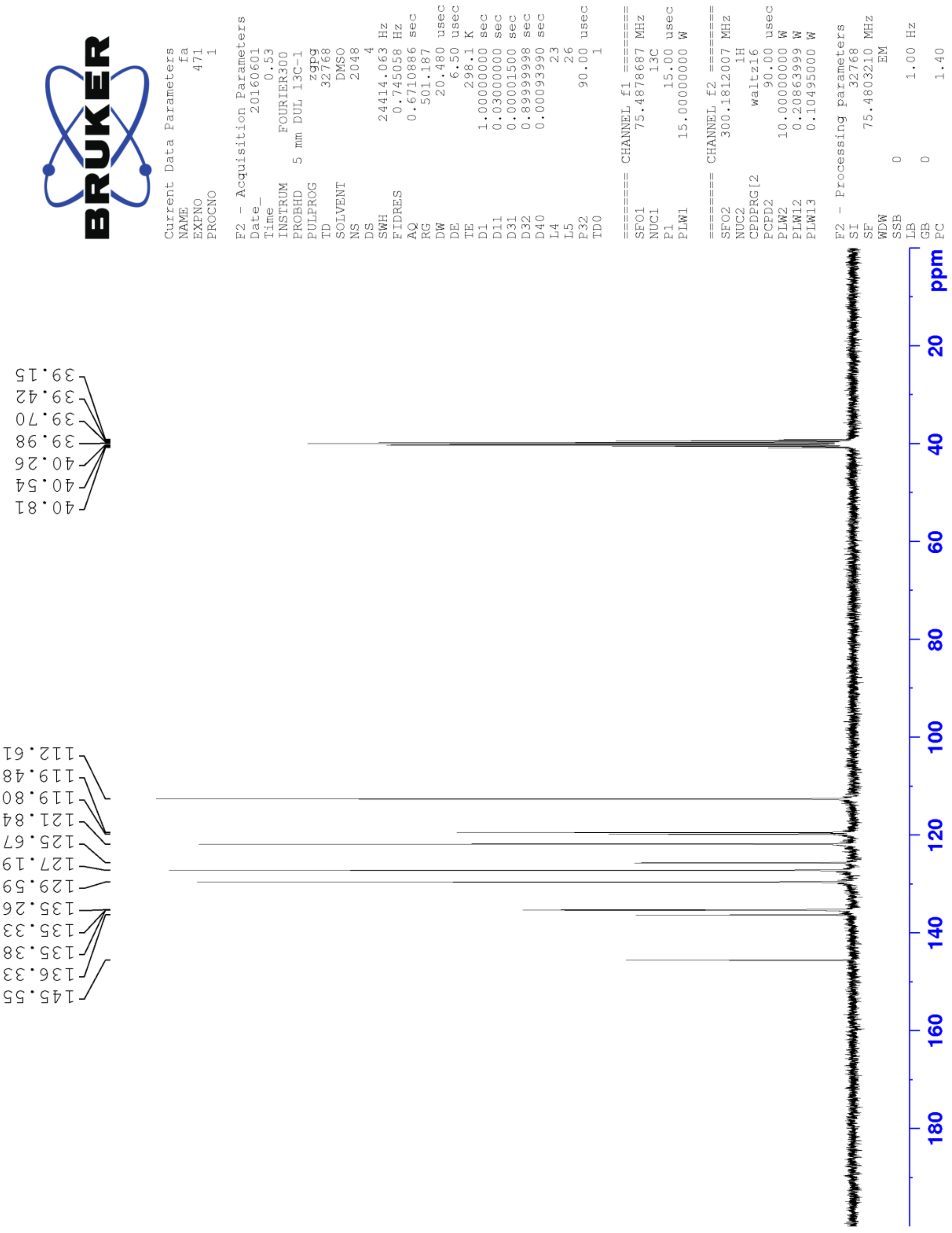

Spectra 35. ^{13}C NMR spectra of the compound 2i

Data File: C:\ LabSolutions\ Data\Analiz\derya\ FA-9_9.lcd

Elmt	Val.	Min	Max	Elmt	Val.	Min	Max	Elmt	Val.	Min	Max	Use Adduct
H	1	0	50	O	2	0	6	Cl	1	0	0	H
C	4	0	50	F	1	0	1	Br	1	0	0	
N	3	0	5	S	2	0	1	Pd	2	0	0	

Error Margin (ppm):	5	DBE Range:	10.0 - 20.0	Electron Ions:	both
HC Ratio:	unlimited	Apply N Rule:	yes	Use MSn Info:	no
Max Isotopes:	3	Isotope RI (%):	1.00	Isotope Res:	10000
MSn Iso RI (%):	10.00	MSn Logic Mode:	AND	Max Results:	500

Event#: 1 MS(E+) Ret. Time : 4.400 -> 4.520 Scan# : 661 -> 679

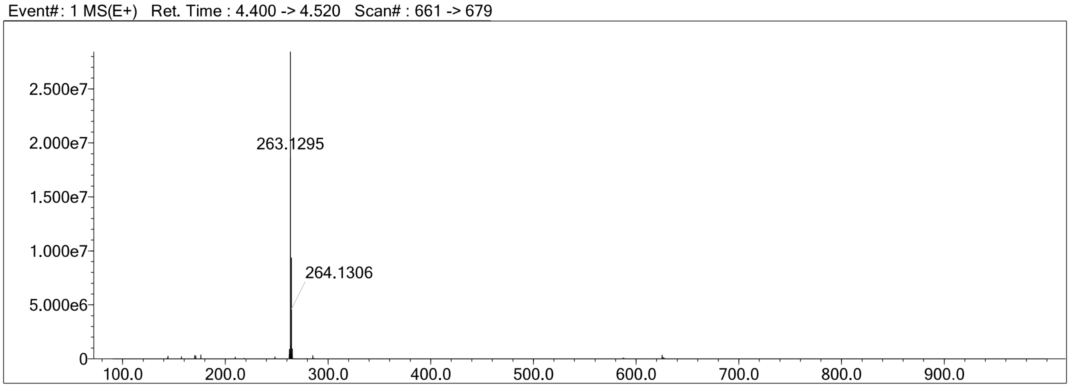

Measured region for 263.1295 m/z

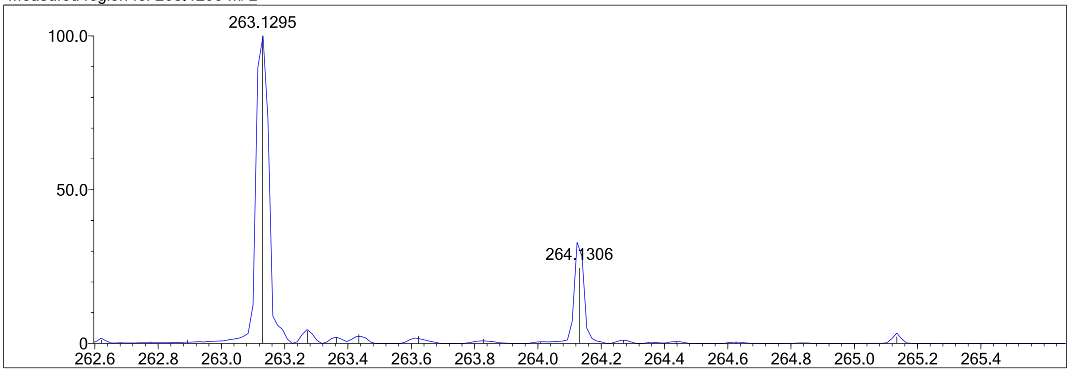

C16 H14 N4 [M+H]+ : Predicted region for 263.1291 m/z

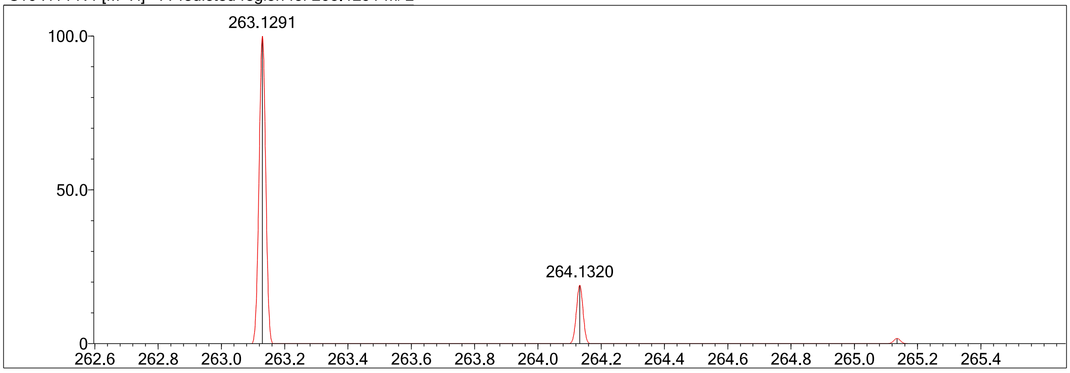

Rank	Score	Formula (M)	Ion	Meas. m/z	Pred. m/z	Df. (mDa)	Df. (ppm)	Iso	DBE
1	74.65	C16 H14 N4	[M+H]+	263.1295	263.1291	0.4	1.52	75.64	12.0

Spectra 36. HRMS spectra of the compound 2i

DOPNALAB

	Item	Value
	Acquired Date&Time	5.10.2016 10:21:39
	Acquired by	System Administrator
	Filename	C:\Users\dopnalab\Desktop\derya\fa\fa-2j1.ispd
	Spectrum name	fa-2j1
	Sample name	fa_2j
	Sample ID	
	Option	
	Comment	
	No. of Scans	10
	Resolution	4 [cm-1]
	Apodization	Happ-Genzel

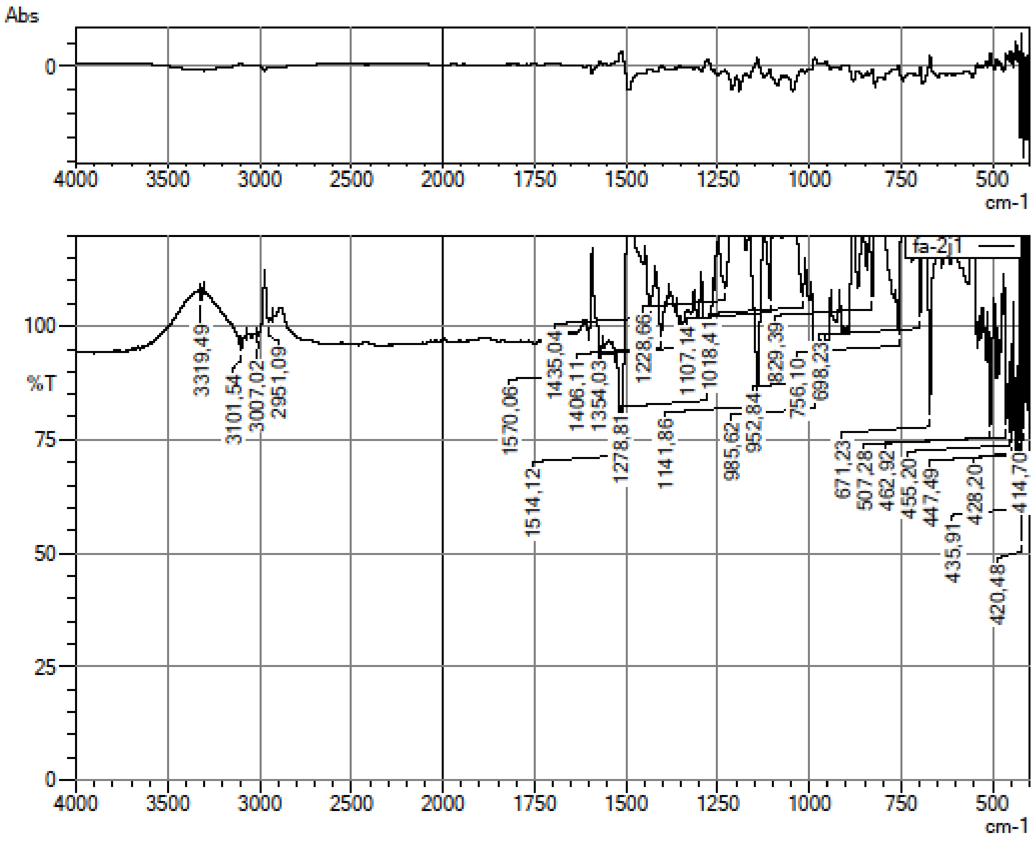

Spectra 37. IR spectra of the compound 2j

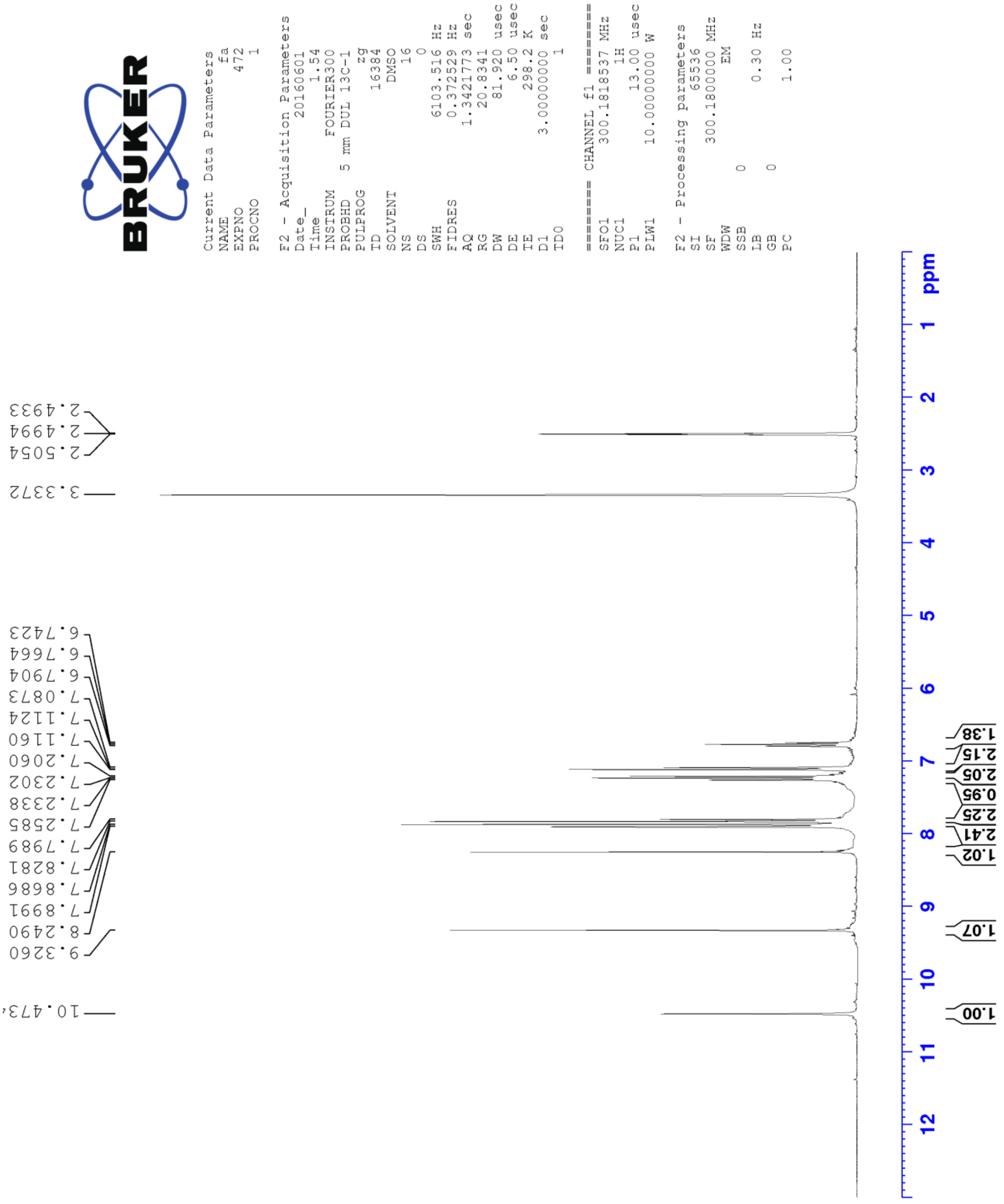

Spectra 38. ^{1}H NMR spectra of the compound 2j

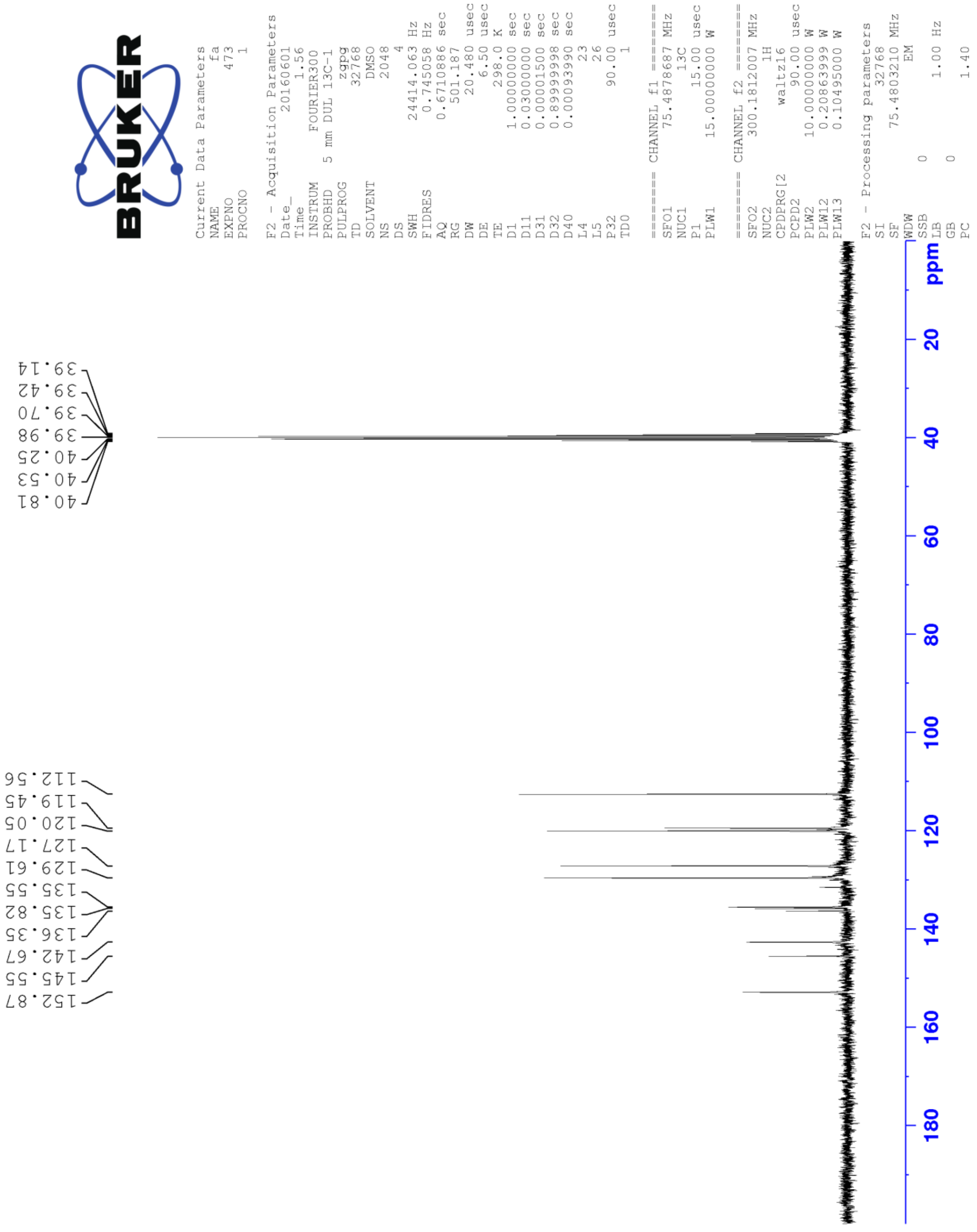

Spectra 39. ^{13}C NMR spectra of the compound 2j

Data File: C:\ LabSolutions\ Data\Analiz\derya\ FA-10_10.lcd

Elmt	Val.	Min	Max	Elmt	Val.	Min	Max	Elmt	Val.	Min	Max	Use Adduct
H	1	0	50	O	2	0	6	Cl	1	0	0	H
C	4	0	50	F	1	0	1	Br	1	0	0	
N	3	0	5	S	2	0	1	Pd	2	0	0	

Error Margin (ppm):	10	DBE Range:	10.0 - 20.0	Electron Ions:	both
HC Ratio:	unlimited	Apply N Rule:	yes	Use MSn Info:	no
Max Isotopes:	3	Isotope RI (%):	1.00	Isotope Res:	10000
MSn Iso RI (%):	10.00	MSn Logic Mode:	AND	Max Results:	500

Event#: 1 MS(E+) Ret. Time : 6.947 Scan# : 1043

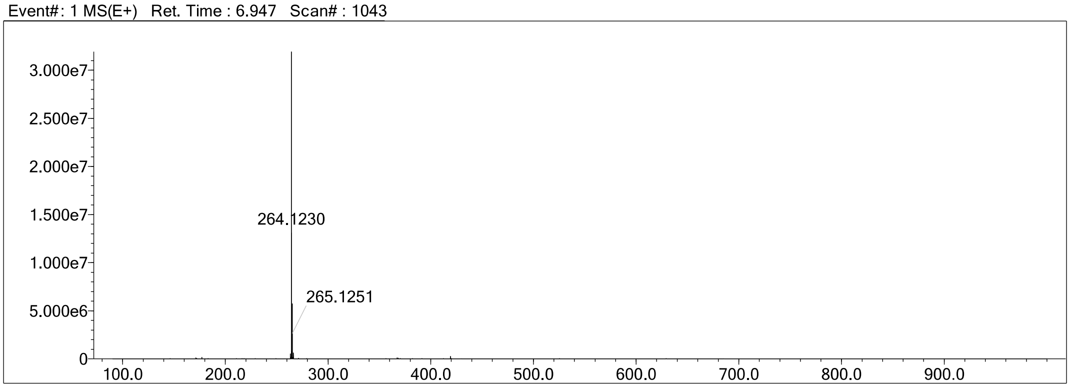

Measured region for 264.1230 m/z

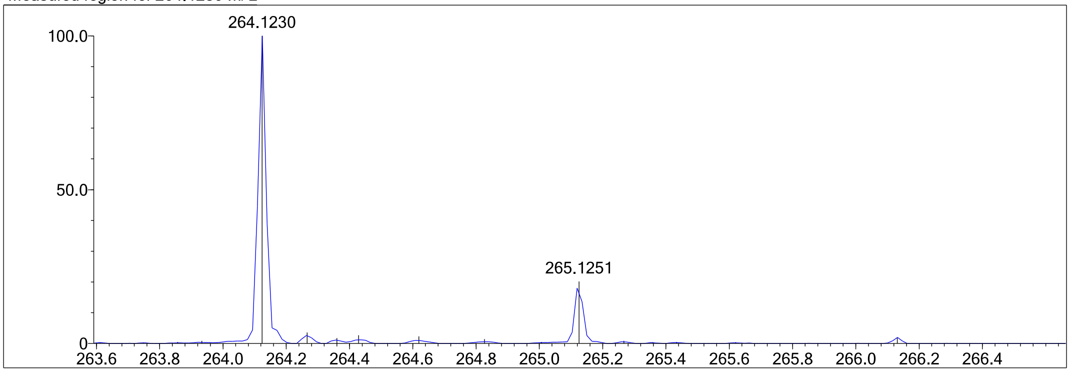

C15 H13 N5 [M+H]+ : Predicted region for 264.1244 m/z

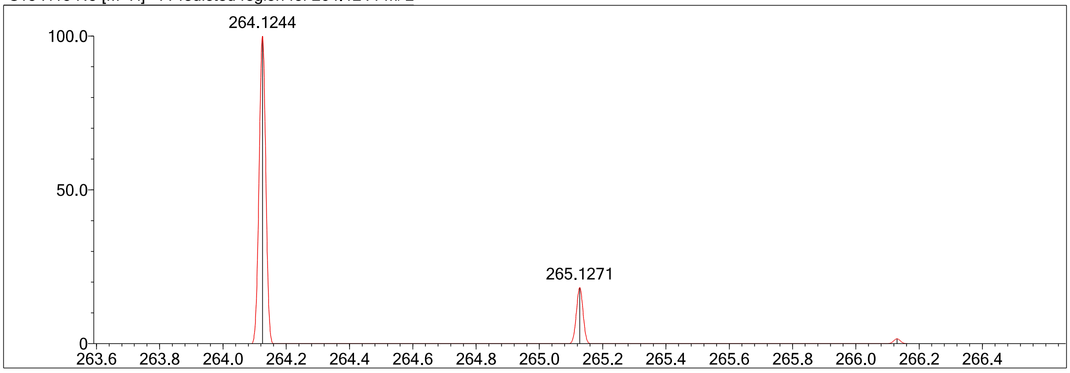

Rank	Score	Formula (M)	Ion	Meas. m/z	Pred. m/z	Df. (mDa)	Df. (ppm)	Iso	DBE
1	82.79	C15 H13 N5	[M+H]+	264.1230	264.1244	-1.4	-5.30	95.17	12.0

Spectra 40. HRMS spectra of the compound 2j

DOPNALAB

	Item	Value
	Acquired Date&Time	7.08.2017 10:39:00
	Acquired by	System Administrator
	Filename	C:\Users\dopnalab\Desktop\derya\fa\fa-141.ispd
	Spectrum name	fa-141
	Sample name	fa-14
	Sample ID	
	Option	
	Comment	
	No. of Scans	10
	Resolution	4 [cm-1]
	Apodization	Happ-Genzel

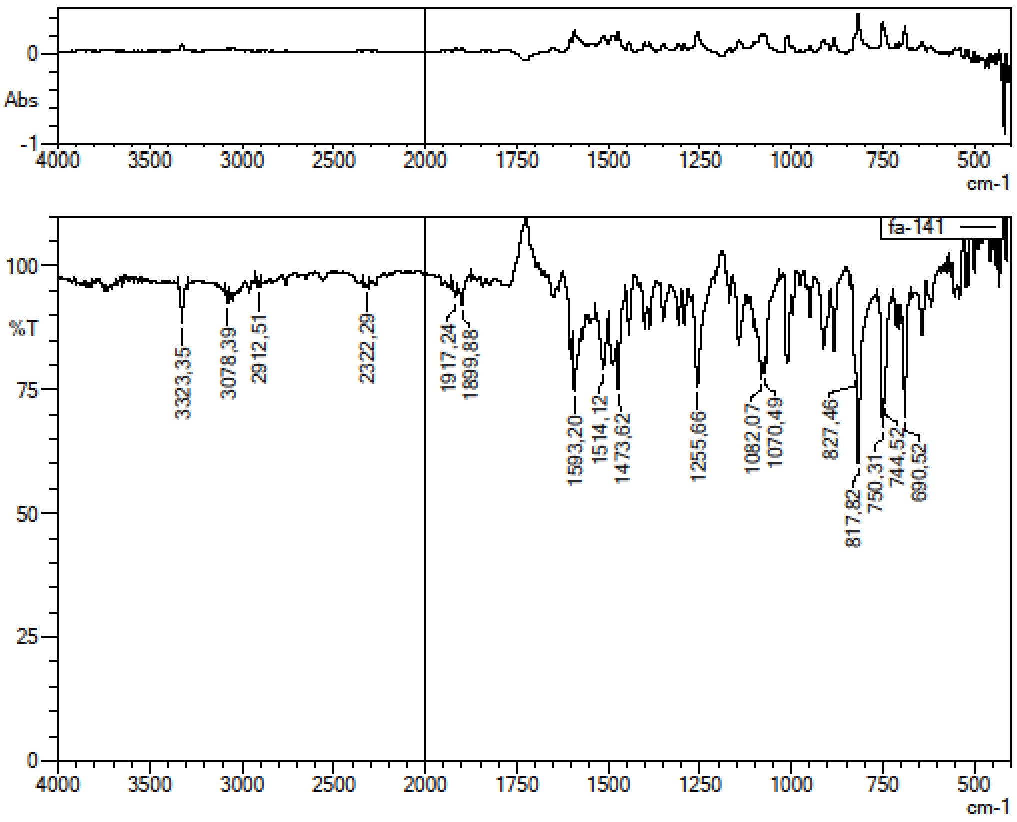

Spectra 41. IR spectra of the compound 2k

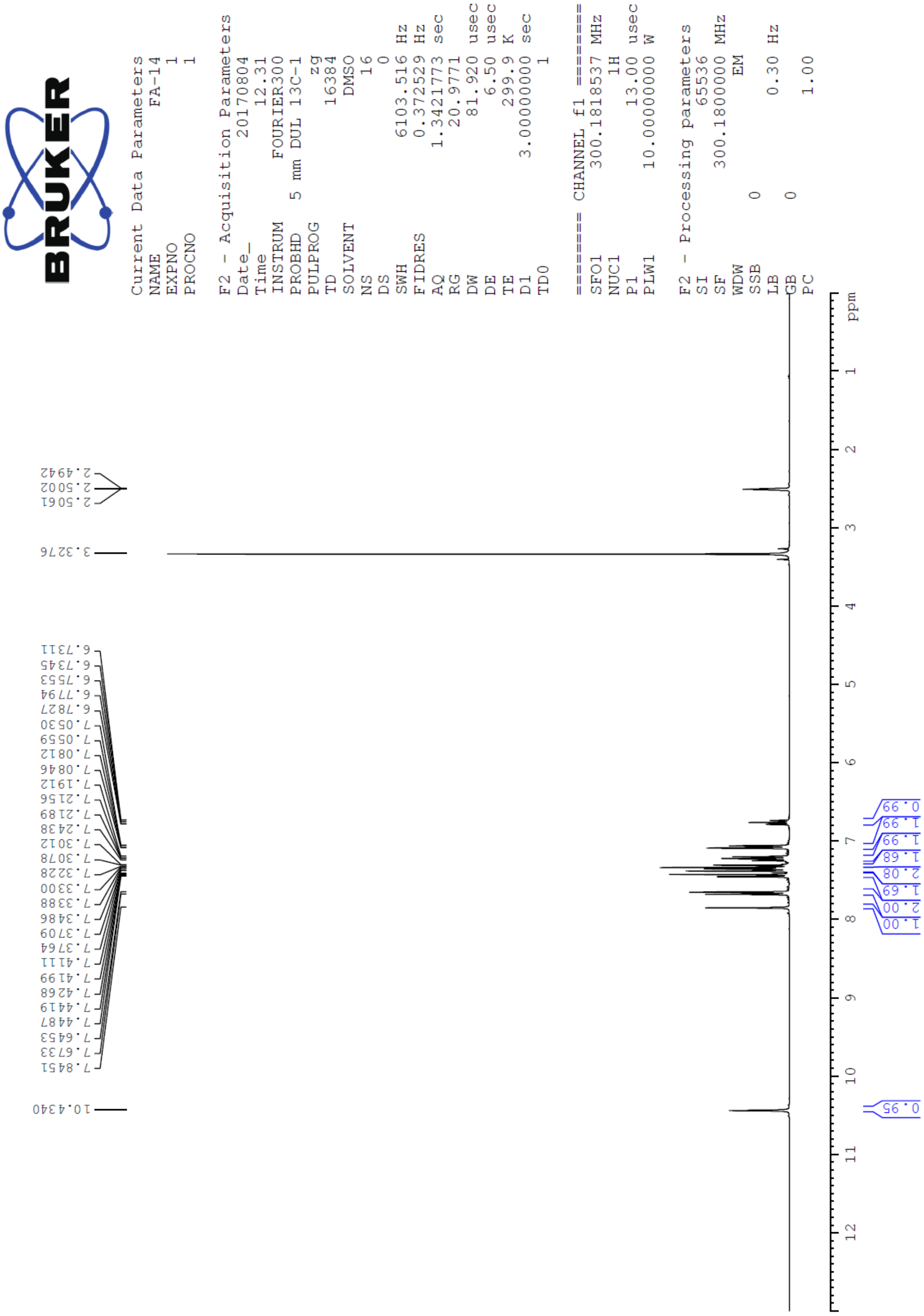

Spectra 42. ^{1}H NMR spectra of the compound 2k

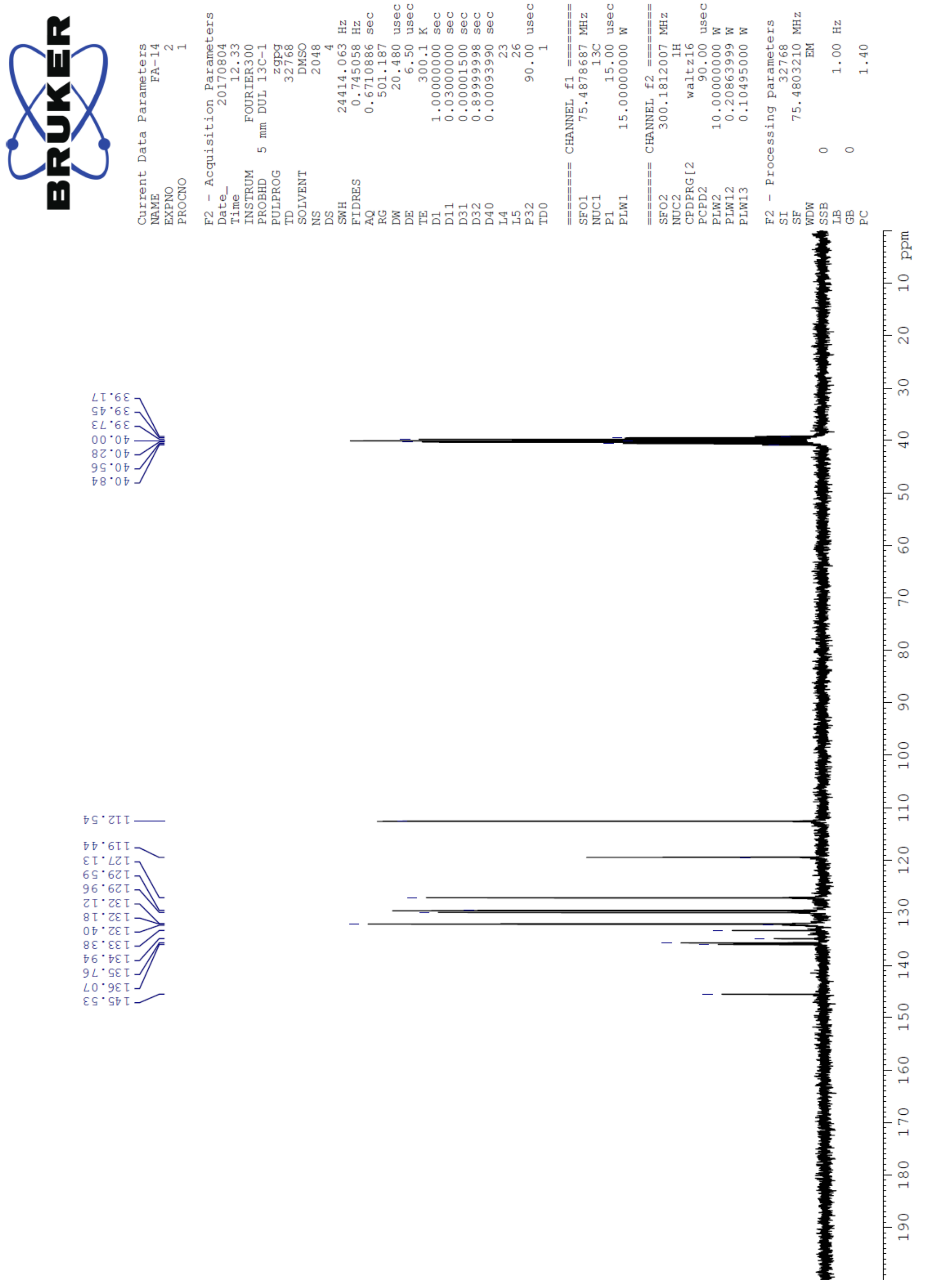

Spectra 43. ^{13}C NMR spectra of the compound 2k

LCMSMS ANALYSIS REPORT

Sample Name	: FA-14		
Sample ID	:		
Data Filename	: FA-14_004.lcd		
Method Filename	: geneltarama.lcm		
Batch Filename	: batch.lcb		
Vial #	: 1-79		
Injection Volume	: 1 uL	Sample Type	: Unknown
Date Acquired	: 04.08.2017 14:52:32	Acquired by	: System Administrator
Date Processed	: 04.08.2017 14:54:35	Processed by	: System Administrator

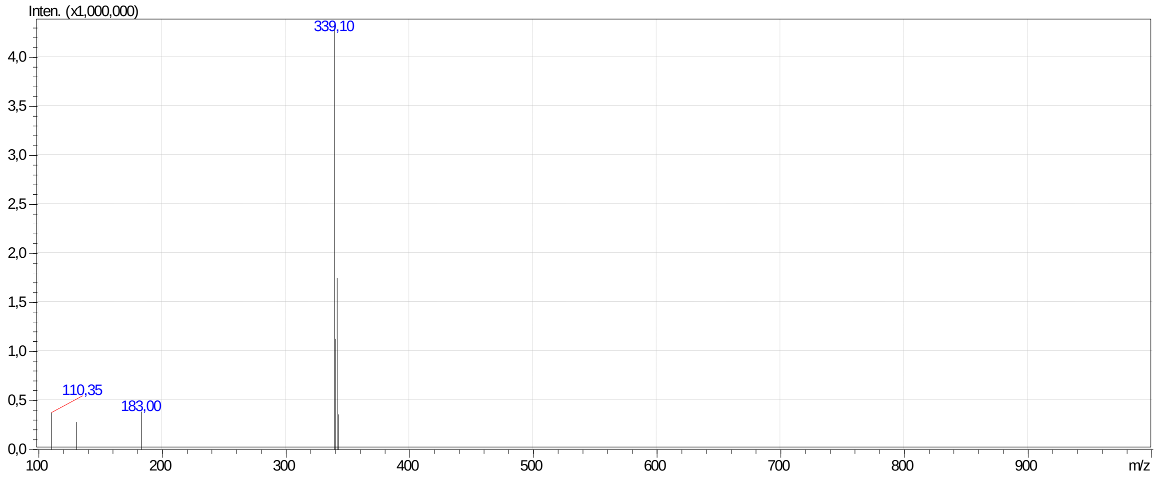

[MS Spectrum]		
# of Peaks	7	
Raw Spectrum	[1,590],(scan:[95])	
Background	[0,068->0,203],(scan:[5->13])	
Base Peak	m/z 339,10 (Inten : 4.335.027)	
m/z	Absolute Intensity	Relative Intensity
110,35	377521	8,71
130,70	279346	6,44
183,00	385160	8,88
339,10	4335027	100,00
340,10	1128654	26,04
341,20	1751529	40,40
342,15	357781	8,25
Event	1	

Spectra 44. LCMSMS spectra of the compound 2k

DOPNALAB

	Item	Value
	Acquired Date&Time	7.08.2017 10:36:28
	Acquired by	System Administrator
	Filename	C:\Users\dopnalab\Desktop\derya\fa\fa-131.ispd
	Spectrum name	fa-131
	Sample name	fa-13
	Sample ID	
	Option	
	Comment	
	No. of Scans	10
	Resolution	4 [cm-1]
	Apodization	Happ-Genzel

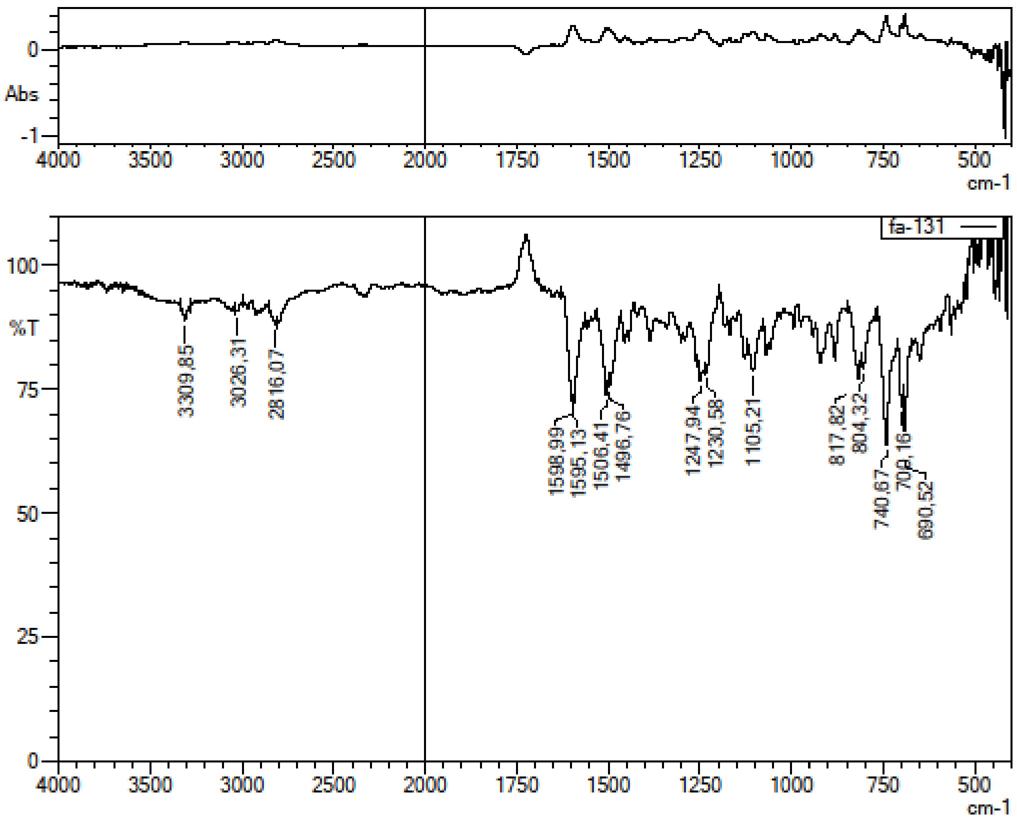

Spectra 45. IR spectra of the compound 2l

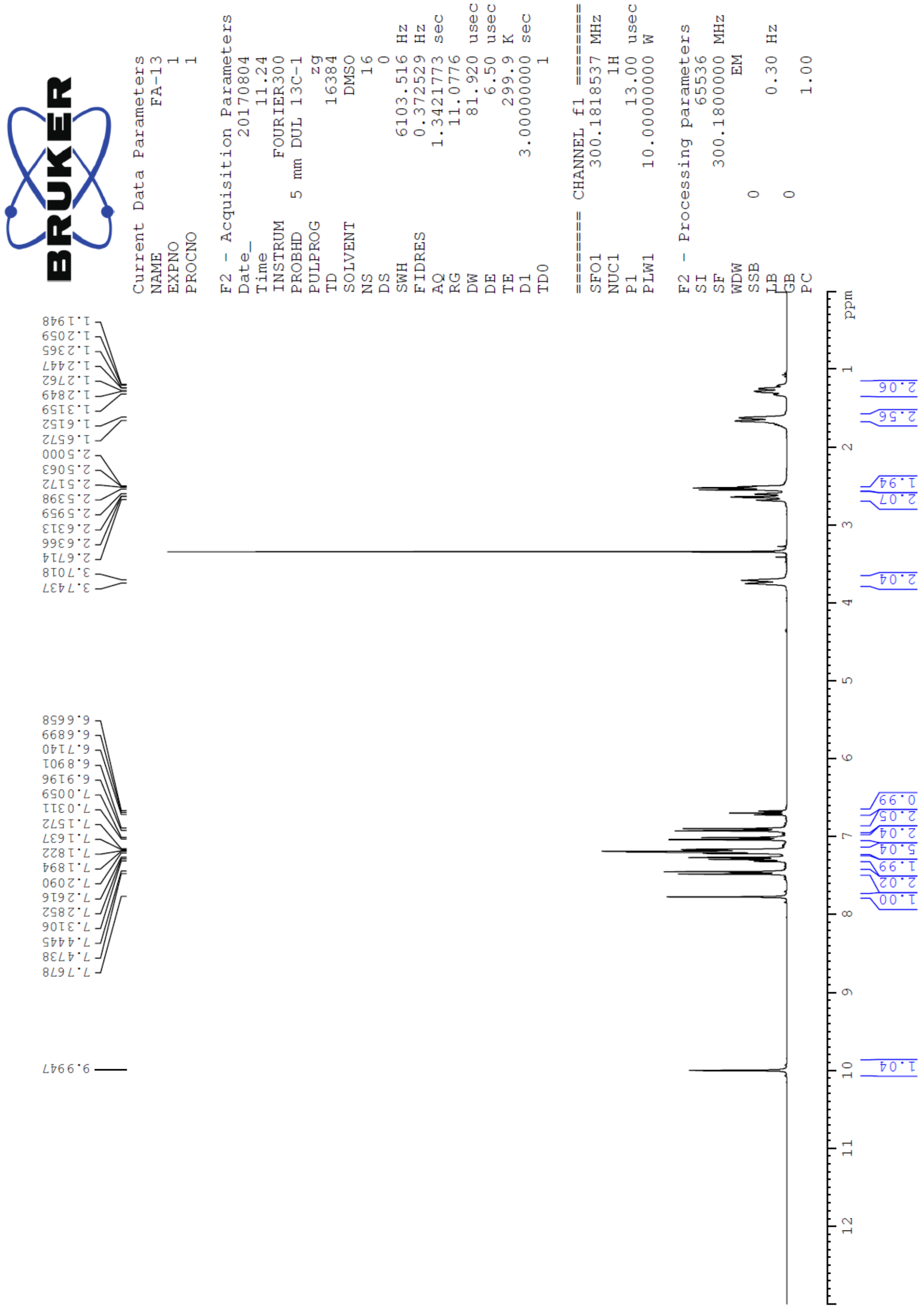

Spectra 46. ^{1}H NMR spectra of the compound 2l

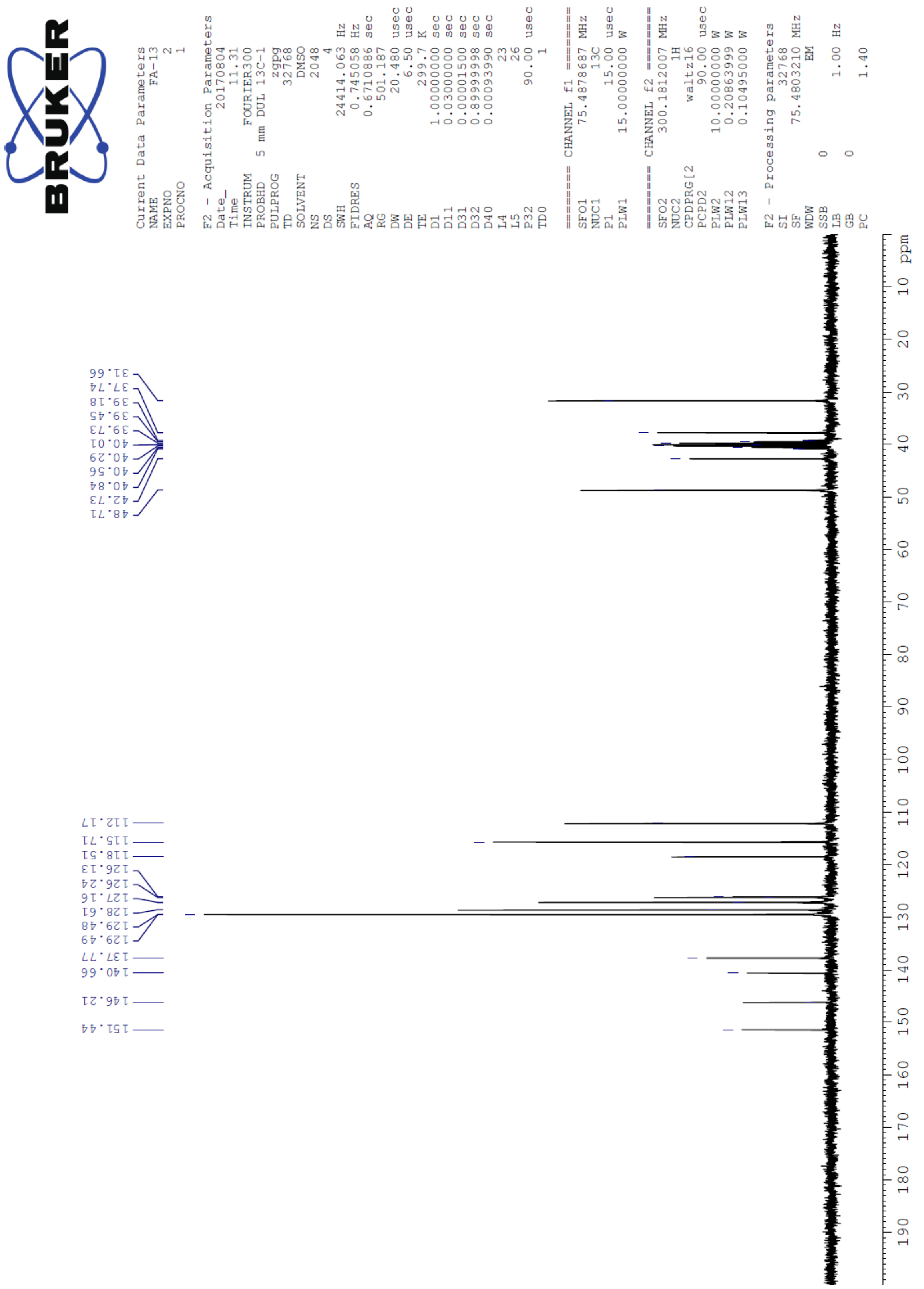

Spectra 47. ^{13}C NMR spectra of the compound 2l

LCMSMS ANALYSIS REPORT

Sample Name	: FA-13		
Sample ID	:		
Data Filename	: FA-13_003.lcd		
Method Filename	: geneltarama.lcm		
Batch Filename	: batch.lcb		
Vial #	: 1-78		
Injection Volume	: 1 uL	Sample Type	: Unknown
Date Acquired	: 04.08.2017 14:49:53	Acquired by	: System Administrator
Date Processed	: 04.08.2017 14:51:56	Processed by	: System Administrator

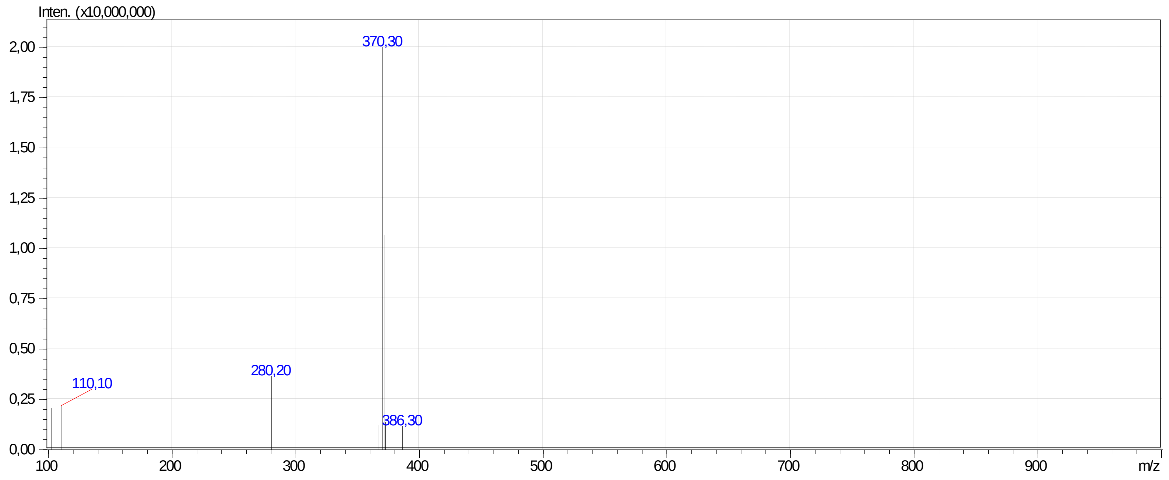

[MS Spectrum]		
# of Peaks	8	
Raw Spectrum	[1,590],(scan:[95])	
Background	No Background Spectrum	
Base Peak	m/z 370,30 (Inten : 20.000.000)	
m/z	Absolute Intensity	Relative Intensity
102,15	2086434	10,43
110,10	2194096	10,97
280,20	3659206	18,30
366,35	1214546	6,07
370,30	20000000	100,00
371,30	10672056	53,36
372,40	1362345	6,81
386,30	1181229	5,91
Event	1	

Spectra 48. LCMSMS spectra of the compound 2l

DOPNALAB

	Item	Value
	Acquired Date&Time	7.08.2017 10:28:53
	Acquired by	System Administrator
	Filename	C:\Users\dopnalab\Desktop\derya\fa\fa-111.ispd
	Spectrum name	fa-111
	Sample name	fa-11
	Sample ID	
	Option	
	Comment	
	No. of Scans	10
	Resolution	4 [cm-1]
	Apodization	Happ-Genzel

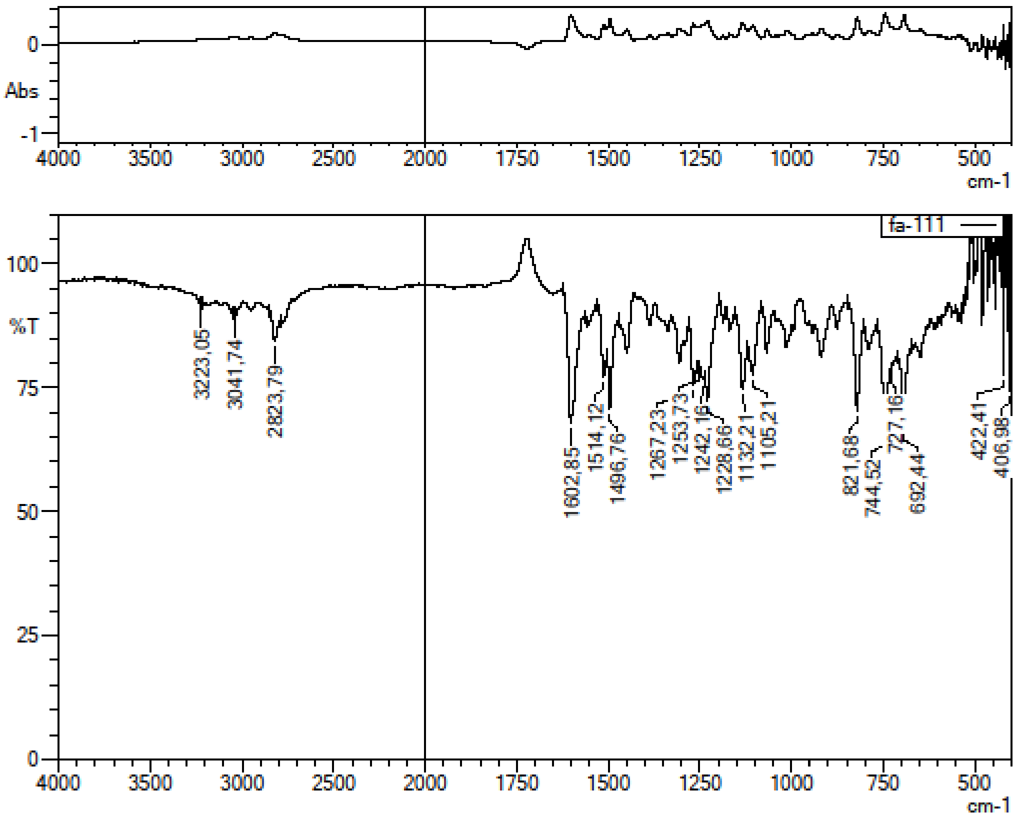

Spectra 49. IR spectra of the compound 2m

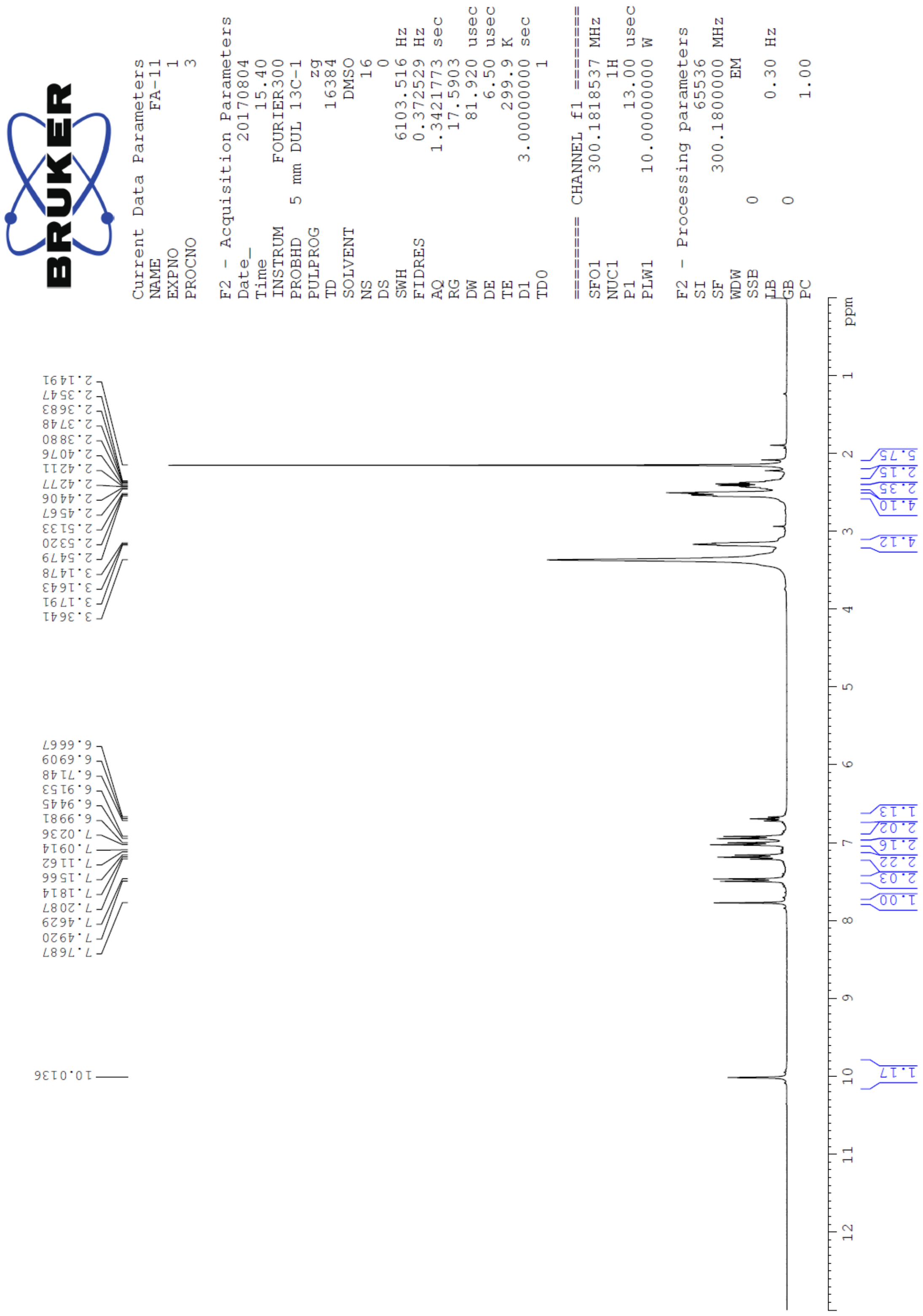

Spectra 50. ^{1}H NMR spectra of the compound 2m

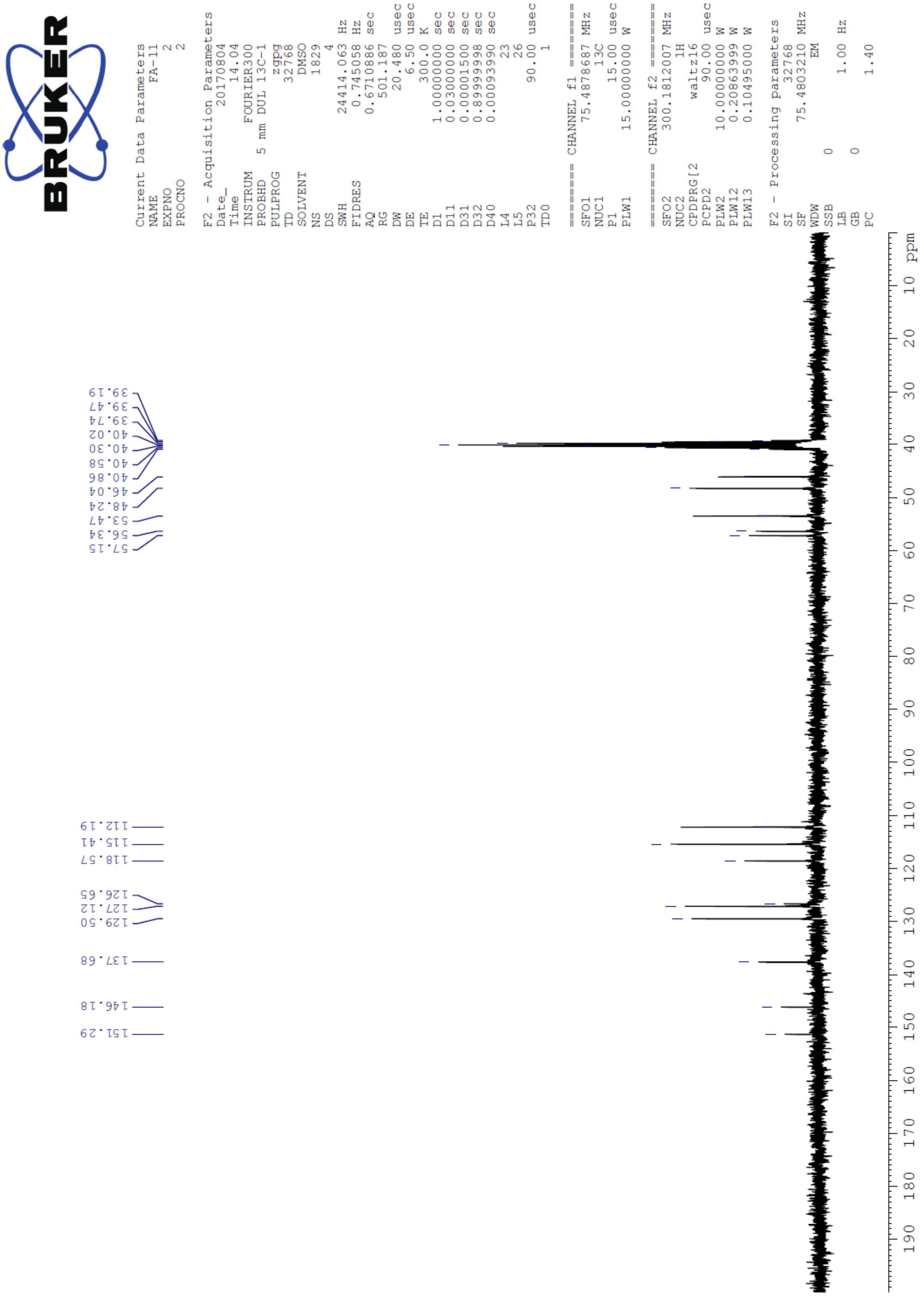

Spectra 51. ^{13}C NMR spectra of the compound 2m

LCMSMS ANALYSIS REPORT

Sample Name	: FA-11		
Sample ID	:		
Data Filename	: FA-11_001.lcd		
Method Filename	: geneltarama.lcm		
Batch Filename	: batch.lcb		
Vial #	: 1-76		
Injection Volume	: 1 uL	Sample Type	: Unknown
Date Acquired	: 04.08.2017 14:44:33	Acquired by	: System Administrator
Date Processed	: 04.08.2017 14:46:36	Processed by	: System Administrator

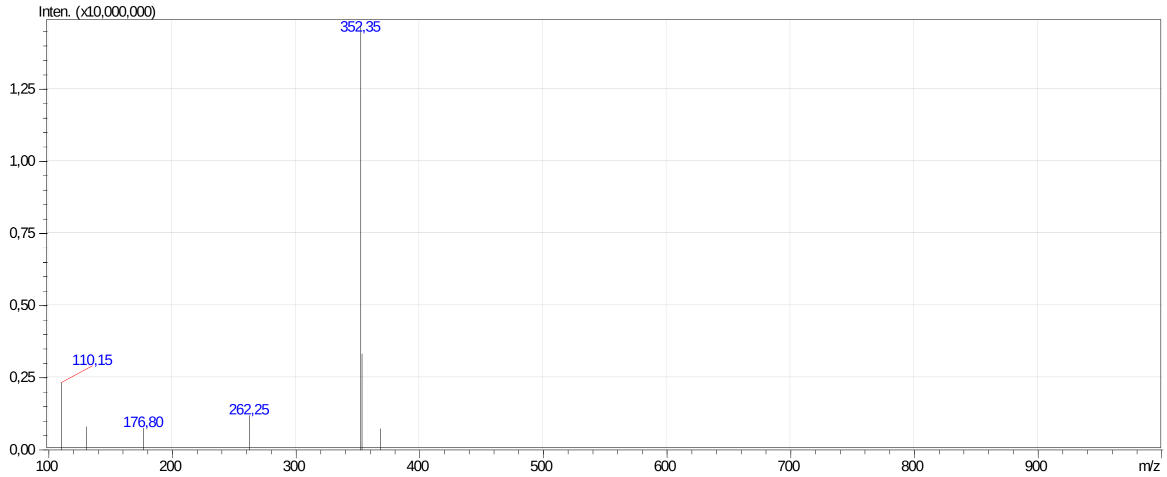

[MS Spectrum]		
# of Peaks	7	
Raw Spectrum	[1,624->1,759],(scan:[97->105])	
Background	[0,101->0,271],(scan:[7->17])	
Base Peak	m/z 352,35 (Inten : 14.730.228)	
m/z	Absolute Intensity	Relative Intensity
110,15	2341968	15,90
130,70	807890	5,48
176,80	761119	5,17
262,25	1201700	8,16
352,35	14730228	100,00
353,40	3336602	22,65
368,30	745511	5,06
Event	1	

Spectra 52. LCMSMS spectra of the compound 2m

DOPNALAB

	Item	Value
	Acquired Date&Time	7.08.2017 10:33:21
	Acquired by	System Administrator
	Filename	C:\Users\dopnalab\Desktop\derya\fa\fa-121.ispd
	Spectrum name	fa-121
	Sample name	fa-12
	Sample ID	
	Option	
	Comment	
	No. of Scans	10
	Resolution	4 [cm-1]
	Apodization	Happ-Genzel

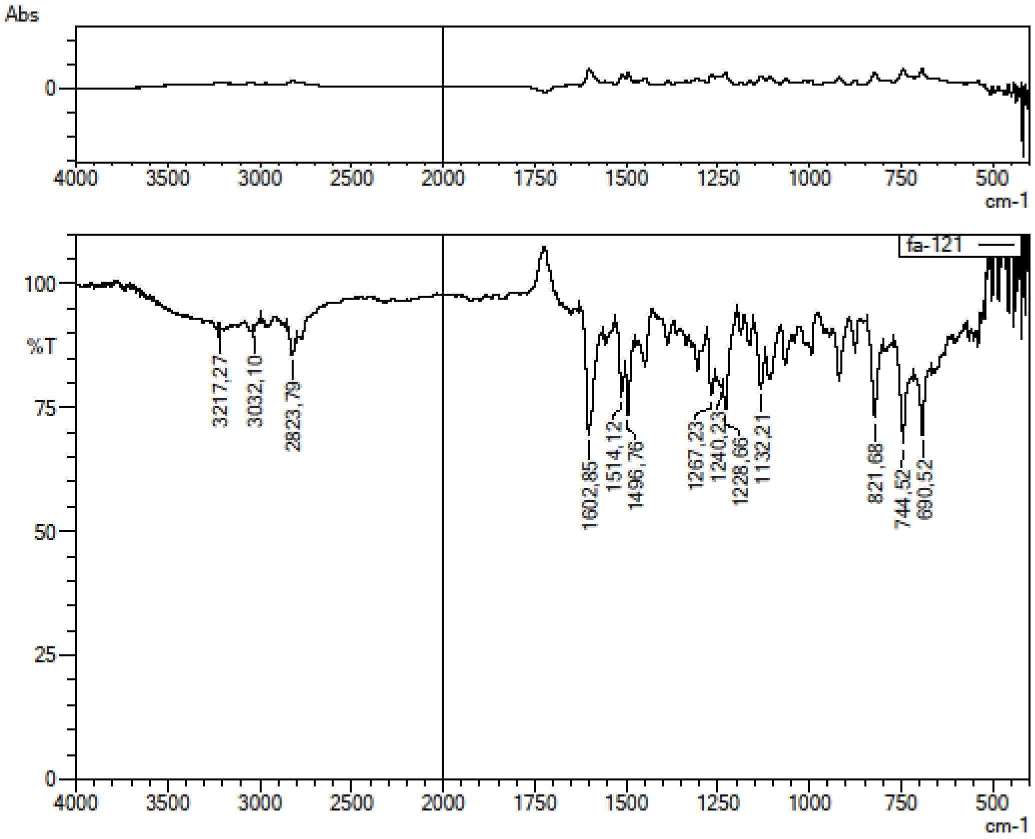

Spectra 53. IR spectra of the compound 2n

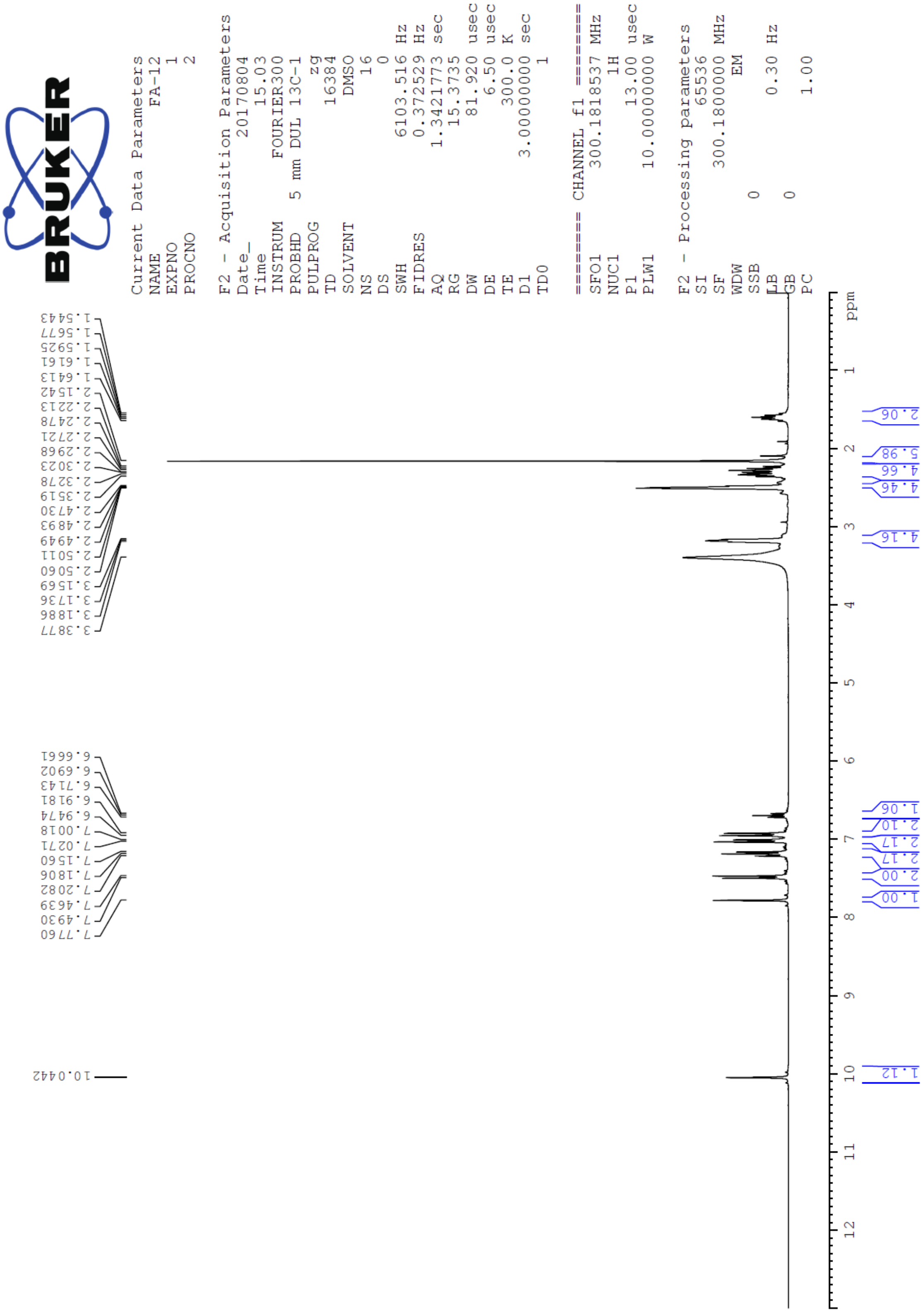

Spectra 54. ^{1}H NMR spectra of the compound 2n

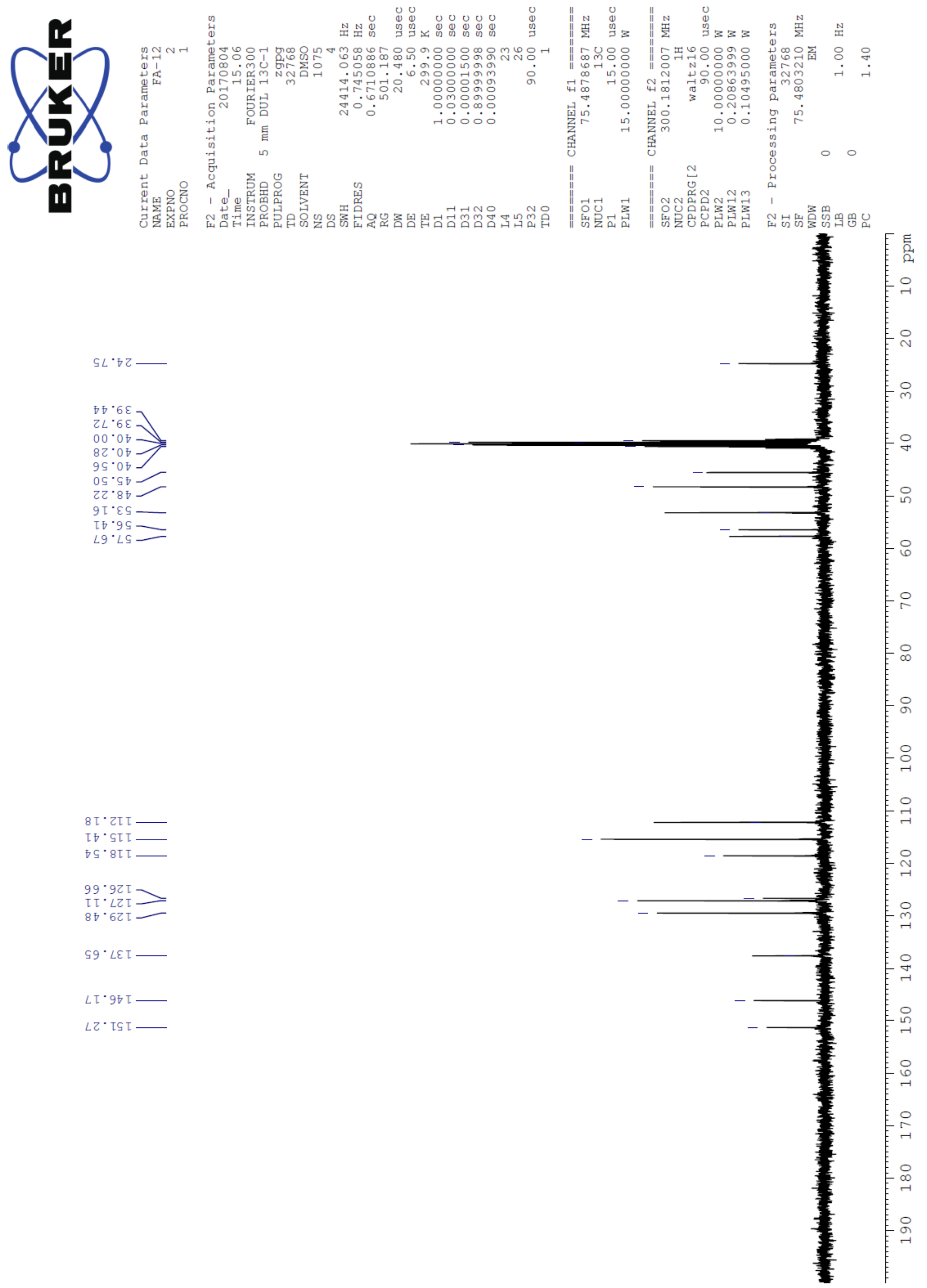

Spectra 55. ^{13}C NMR spectra of the compound 2n

LCMSMS ANALYSIS REPORT

Sample Name	: FA-12		
Sample ID	:		
Data Filename	: FA-12_002.lcd		
Method Filename	: geneltarama.lcm		
Batch Filename	: batch.lcb		
Vial #	: 1-77		
Injection Volume	: 1 uL	Sample Type	: Unknown
Date Acquired	: 04.08.2017 14:47:13	Acquired by	: System Administrator
Date Processed	: 04.08.2017 14:49:17	Processed by	: System Administrator

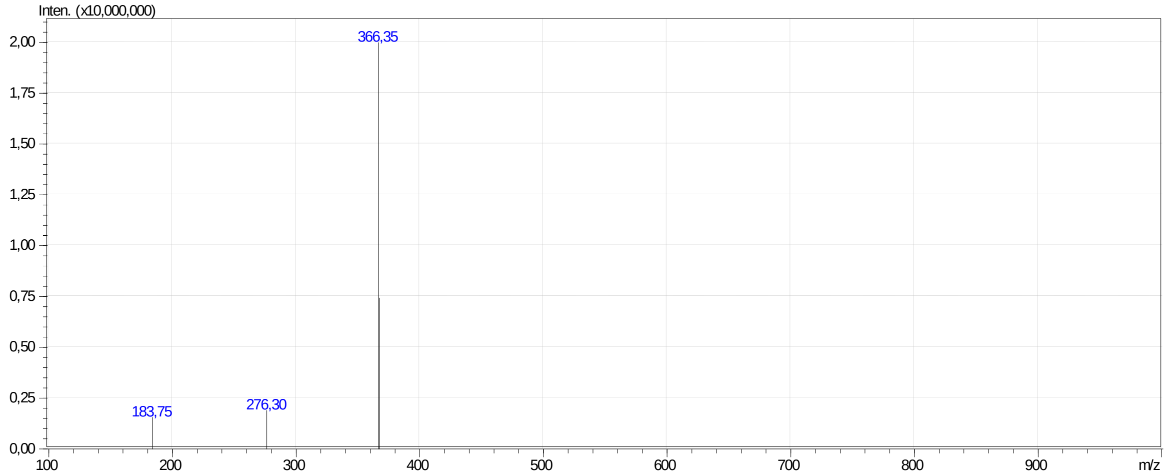

[MS Spectrum]

# of Peaks	4	
Raw Spectrum	[1,590],(scan:[95])	
Background	[0,034->0,135],(scan:[3->9])	
Base Peak	m/z 366,35 (Inten : 20.000.000)	
m/z	Absolute Intensity	Relative Intensity
183,75	1572286	7,86
276,30	1907242	9,54
366,35	20000000	100,00
367,40	7431947	37,16
Event	1	

Spectra 56. LCMSMS spectra of the compound 2n
